# Recent Advances in the Synthesis of Selenophenes and Their Derivatives

**DOI:** 10.3390/molecules25245907

**Published:** 2020-12-13

**Authors:** Paola S. Hellwig, Thiago J. Peglow, Filipe Penteado, Luana Bagnoli, Gelson Perin, Eder J. Lenardão

**Affiliations:** 1 Laboratório de Síntese Orgânica Limpa-LASOL-CCQFA, Universidade Federal de Pelotas-UFPel, P.O. Box 354, 96010-900 Pelotas, RS, Brazil; psh.1996@hotmail.com (P.S.H.); thiago_lasol@yahoo.com.br (T.J.P.); penteado.filipe@gmail.com (F.P.); 2 Group of Catalysis, Synthesis and Organic Green Chemistry, Department of Pharmaceutical Sciences, University of Perugia, Via del Liceo 1, 06123 Perugia, Italy; luana.bagnoli@unipg.it

**Keywords:** organochalcogen, selenophene, benzoselenophene, cyclization, organoselenium

## Abstract

The selenophene derivatives are an important class of selenium-based heterocyclics. These compounds play an important role in prospecting new drugs, as well as in the development of new light-emitting materials. During the last years, several methods have been emerging to access the selenophene scaffold, employing a diversity of cyclization-based synthetic strategies, involving specific reaction partners and particularities. This review presents a comprehensive discussion on the recent advances in the synthesis of selenophene-based compounds, starting from different precursors, highlighting the main differences, the advantages, and limitations among them.

## 1. Introduction

The importance of organoselenium compounds is linked to their synthetic applications as catalysts [1,2,3,4] and building blocks [5,6,7,8,9], allowing for several chemo-, regio- and stereoselective transformations [10,11,12,13,14,15]. Although organoselenium compounds have not yet reached the pharmacy shelves, studies in vitro and in vivo have demonstrated their effectiveness against several disorders [16,17,18,19]. Among the successful examples, those molecules presenting an organoselenium portion embedded in an aromatic or non-aromatic cycle—i.e., the selenium-containing heterocycles—have demonstrated powerful biological activities [20,21].

**Figure 1:**
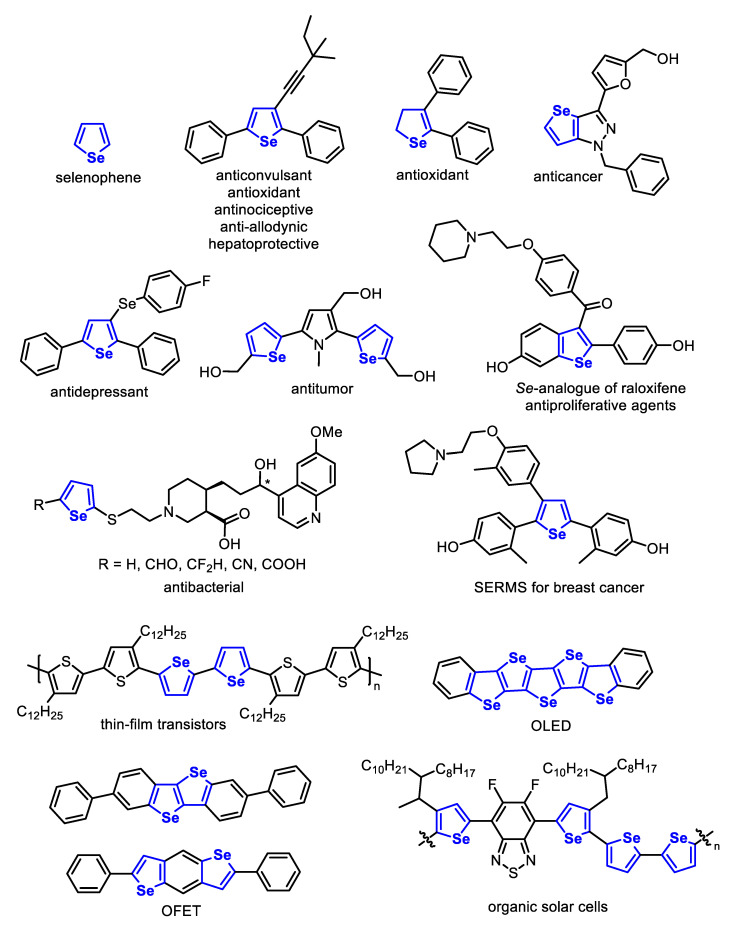
Selected examples of biologically active and technologically interesting selenophenes.

In this context, selenophenes and their derivatives have been widely studied due to their intrinsic biological activity [22], such as antidepressant [23,24,25,26,27,28], antioxidant [29,30,31,32,33,34,35], anticonvulsant [36,37], antibacterial [38], antitumor [39,40,41,42,43,44,45,46,47], antinociceptive [48], hepatoprotective [49,50] and antiapoptotic agents [51]. Besides, they have also played an important role in the materials science field, being used to build organic light emitting diodes (OLEDs) [52,53,54,55,56,57,58], organic field effect transistors (OFETs) [59,60,61,62,63,64,65,66,67,68,69], organic solar cells (OSC) [70,71,72,73,74,75,76,77,78,79,80,81,82] and thin-film transistors [83,84,85,86,87,88,89,90]. The structures of several biologically active and technologically interesting selenophenes are presented in Figure 1.

Selenophene derivatives have also been used as ligands in coordination chemistry [91,92,93,94,95,96,97]. After a pre-activation with a halide or an organometallic (B, Li, Mg, Sn or Zn), these compounds can be employed as reagents in the formation of new C-C [98,99,100,101,102,103,104,105,106,107], C-N [108,109,110] and C-S [111] bonds under Pd or Cu catalysis, through Heck, Stille, Negish, Kumada, Suzuki and Sonogashira coupling reactions. In recent years, unactivated selenophenes have been used as reagents in several synthetic transformations by palladium-catalyzed direct C-H bond activation [112,113,114,115,116].

**Scheme 1:**
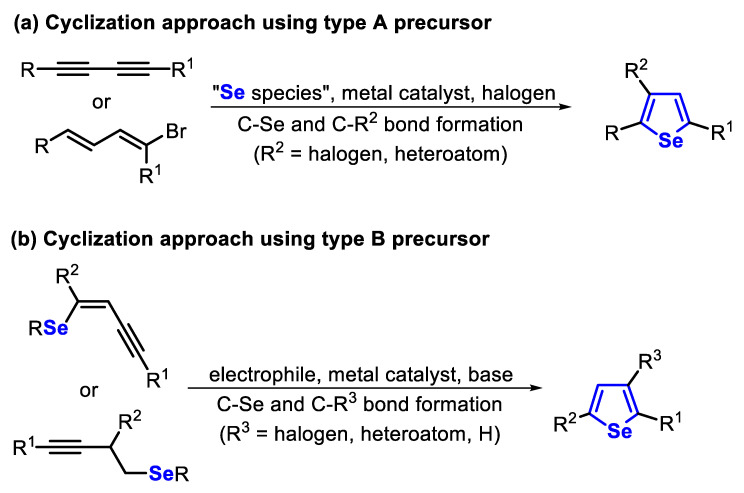
Two common protocols to the synthesis of selenophenes.

The traditional methods for the synthesis of selenophenes involve the addition of a selenium-based nucleophile or electrophile to an appropriate acyclic precursor containing a π-system, followed by an intramolecular cyclization (type A precursor). Alternatively, a previously prepared organoselenium precursor can easily undergo an intramolecular cyclization toward selenophenes (type B precursor). In general, these reactions are mediated by TM-based catalysts or electrophilic species (Scheme 1).

**Scheme 2:**
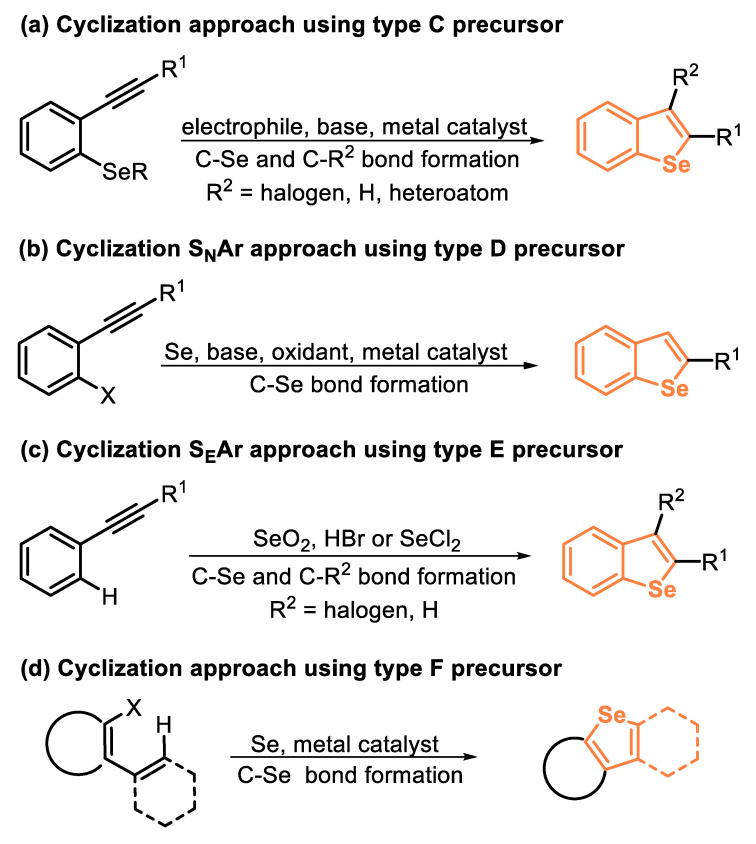
Common protocols used to prepare benzoselenophenes.

Another useful strategy to access benzoselenophene derivatives is through the intramolecular cyclization of selenium-functionalized arenes bearing *ortho*-alkynyl groups. These reactions can be mediated by TM-based catalysts and by electrophilic species (type C precursor). On the other hand, if alkynyl arenes bearing *ortho*-halogen groups are used in the presence of elemental selenium, under appropriate conditions, benzoselenophenes can be accessed through a S_N_Ar reaction, followed by an intramolecular annulation (type D precursor). By replacing the *ortho*-halogen group by a hydrogen atom, a selane Friedel–Crafts cyclization affords the respective benzoselenophene (type E precursor). Finally, benzoselenophene derivatives can be satisfactorily accessed through a tandem cyclization of *ortho*-haloareneheterocycles, in the presence of a TM catalyst and elemental selenium (type F precursor) (Scheme 2).

Thus, considering the importance of the selenophene core, some reviews have been published during the last few decades that describe several strategies to access these compounds [117,118,119,120,121,122,123]. Among them, a short review was recently published [124] covering synthetic methods to access several selenophene derivatives, and the synthetic versatility of selenophenes in the formation of new C-C bonds.

Herein, we present a comprehensive review covering the recent synthetic methods to prepare selenophenes, benzoselenophenes and other heterocycles-fused derivates. For a better discussion and understanding, the range of synthetic methodologies is divided into three major groups: (i) selenophenes, (ii) benzoselenophenes and (iii) fused selenophenes and benzoselenophenes. In each section, methods employing the precursors described in the Scheme 1 and Scheme 2 are discussed first, followed by other important approaches.

## 2. Synthesis of Selenophenes

### 2.1. Starting from Type A Precursors

**Scheme 3:**
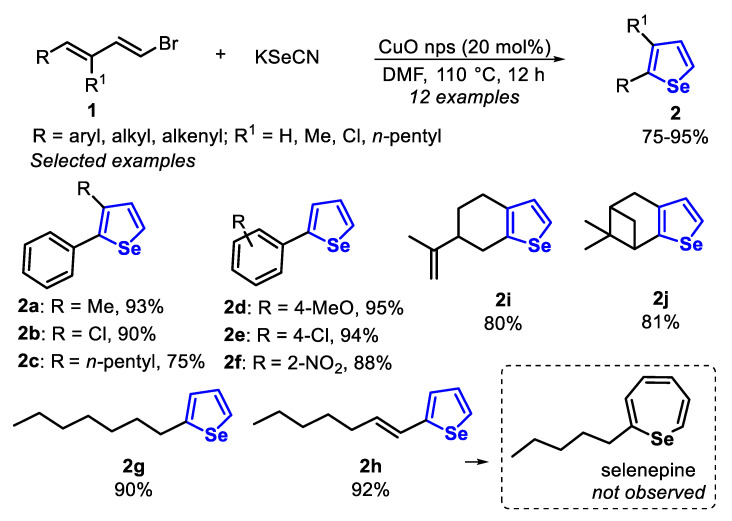
Scope of Cu-catalyzed one-pot synthesis of selenophenes **2** via nucleophilic cyclization.

**Scheme 4:**
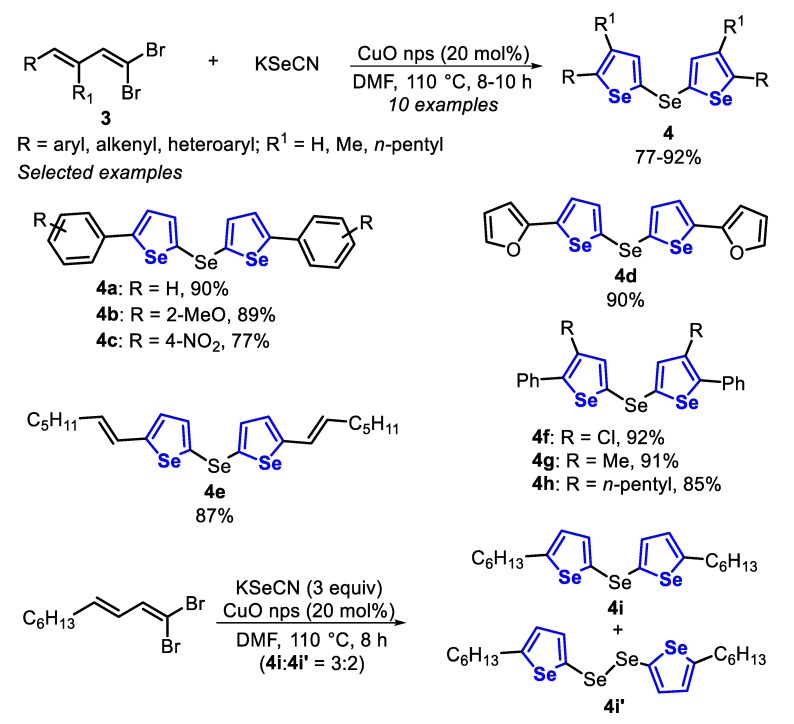
Scope of Cu-catalyzed one-pot synthesis of selanyl selenophenes **4** via nucleophilic cyclization.

The first protocols for the synthesis of selenophenes used as precursors hexane-2,5-dione and phosphorous pentaselenide (Paal, 1885) [125], metallic selenium and acetylene (Briscoe and Peel, 1928) [126], acetylenes and a mixture of bauxite and aluminum selenide (McMahon and co-workers, 1933) [127], acetylene with dimethyl selenide or diselenide (Voronkov and co-workers, 1987) [128], 2,4-alkadienic esters by oxidation reaction with SeO_2_ (Takeda and co-workers, 1986) [129], among others [117], under high temperatures (180–600 °C). Since then, new protocols were developed for milder reaction conditions and that would afford selenophene derivatives in high yields. With this propose, in 2014, the one-pot synthesis of selenophenes **2** and selanyl selenophenes **4** by an intramolecular cyclization of 1,3-dienyl bromides **1** and 1,3-dienyl-*gem*-dibromides **3**, in the presence of KSeCN and CuO nanoparticles (nps) was described [130]. The reactions were carried out in DMF, at 110 °C for 8–12 h (Scheme 3 and Scheme 4). Under this condition, aryl-1,3-dienyl bromides **1** bearing alkyl and electron-withdrawing substituents (R^1^ = Me, Cl and *n*-pentyl) reacted smoothly to produce the 2-phenyl-3-alkyl selenophenes **2a**–**c** in good yields. The protocol was also able to employ 1,3-dienyl bromides **1,** bearing activated and unactivated aryl groups (R = 4-MeO, 4-Cl and 2-NO_2_), affording the respective products **2d**–**f** in excellent yields. Interestingly, 1,3,5-trienyl bromide **3** was able to furnish the 2-vinyl selenophene **2h** in 92% yield, without the formation of selenepine, and with the C-C double bond remaining on the C2 position. Additionally, this protocol was also effective to access the tetrahydro-benzo[*b*]selenophenes **2i** and **2j** in good yields (Scheme 3).

In contrast, when aryl-substituted 1,3-dienyl-*gem*-dibromides **3** were submitted to the standard reaction condition, the corresponding selanyl selenophenes **4a**–**c** were obtained in good yields. In addition, the 2-furyl-1,3-dienyl-*gem*-dibromide and 1,3,5-trienyl-*gem*-dibromide **3** furnished the selanyl selenophenes **4d** and **4e** in good yields. Aryl-1,3-dienyl-*gem*-bromides **3** bearing chloro- or alkyl groups (R^1^ = Cl, Me and *n-*pentyl), afforded the 2-phenyl-3-alkylselanylselenophenes **4f**–**h** in very good to excellent yields. On the other hand, limitations were faced when 1,3-dienyl-*gem*-dibromides **3** (R = *n*-hexyl) were employed, producing a mixture of selanylselenophene **4i** and diselanylselenophene **4i’** in different ratios (Scheme 4).

**Scheme 5:**
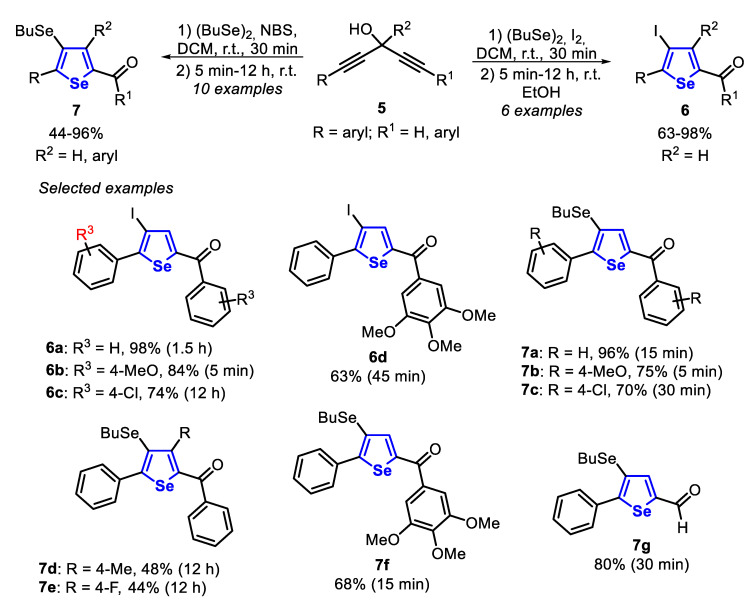
Synthesis of selenophenes via cyclization of diynols using Bu_2_Se_2_ and a halogen source.

In 2015, was reported the synthesis of selenophenes through the cyclization of diynols **5**, promoted by dibutyl diselenide and a halogen source [131]. The reaction optimization studies showed the best reaction condition as being the stirring of a solution of the diynol **5** in DCM, at room temperature, in the presence of dibutyl diselenide (1.5 equiv) and I_2_ or NBS (1.5 equiv), affording the desired products **6** and **7** in moderate to excellent yields. It is worth mentioning that the halogen source (I_2_ or NBS) directly affects the reaction regioselectivity. Thus, employing the symmetrical diynols **5** bearing neutral and electron-donating groups (R^3^ = H and 4-MeO) in the aromatic ring connected to C(*sp*), the products **6a** and **6b** were obtained in 98% and 84% yields, respectively, in short reaction times. However, the presence of an electron-withdrawing substituent (R^3^ = 4-Cl) reduced the reactivity, needing 12 h to access the 4-iodoselenophene **6c** in 74% yield. A noteworthy result was obtained using an unsymmetrical diynol **5**, which efficiently reacted to give the respective selenophene **6d** in 63% yield after 45 min regioselectively. This result suggests that a sterically bulky group, bearing electron-donating groups, positively influences the regioselectivity, by stabilizing the seleniranium reaction intermediate. In contrast, 4-butylselanyl-selenophenes **7** were obtained when NBS was used instead of I_2_ in the reaction of diynol **5** with Bu_2_Se_2_ under the same conditions. In this case, symmetrical diynols **5** bearing neutral, activated and unactivated phenyl groups (R^3^ = H, MeO and Cl) reacted satisfactorily to give the respective selenophenes **7a**–**c** in good to excellent yields, in short reaction times (5–30 min). Additionally, when tertiary diynols **5** were employed as substrates, the 4-butylselanyl-selenophenes **7d** and **7e** were obtained in 48% and 44% yields, respectively, after 12 h. Besides, the use of unsymmetrical diynols **5** led to the exclusive formation of the 4-butylselanylselenophenes **7f** and **7g** in 68% and 80% yields, respectively (Scheme 5).

**Scheme 6:**
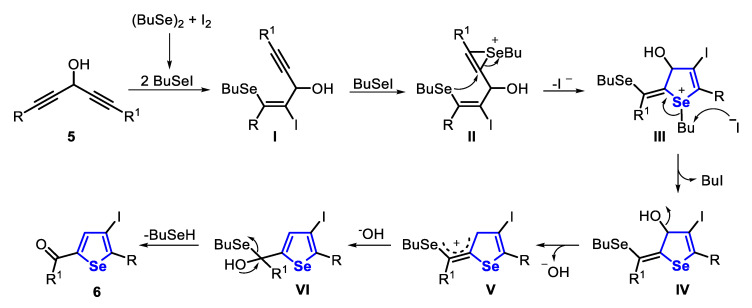
Proposed mechanism of the synthesis of 3-iodoselenophene **6**.

The proposed mechanism of the transformation of the diynols **5** into 4-iodo-selenophenes **6** initiates by the in situ I_2_-promoted oxidation of dibutyl diselenide to give the electrophilic intermediate BuSeI, which is added to the C-C triple bond, affording the *E*-vinyl selenide **I**. In the sequence, another portion of BuSeI reacts with the second C-C triple bond, affording the seleniranium intermediate **II**, which undergoes an intramolecular 5-*exo-dig* cyclization, according to Baldwin’s rules [132], giving the cyclic selenonium intermediate **III**. In the sequence, the intermediate **III** is converted to the dihydroselenophene **IV**, regenerating the electrons to the selenium atom. Thus, the intermediate **IV** undergoes a 1,3-OH-migration, followed by the elimination of BuSeH, generating the desired selenophene **6** (Scheme 6).

**Scheme 7:**
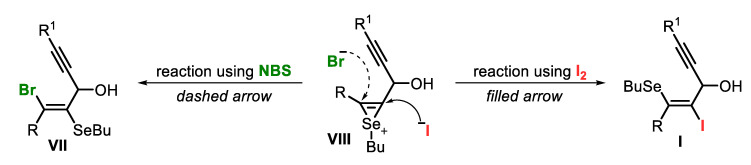
Key intermediates in the synthesis of 4-iodoselenophenes **6** and 4-butylselanylselenophenes **7**.

While the formation of the 4-iodo-selenophenes **6** may involve the formation of vinylic selenide **I** as the key reaction intermediate, instead, the synthesis of 4-butylselanyl-selenophenes **7** passes by the vinylic selenide **VII** (Scheme 7). After the initial formation of the seleniranium derivative **VIII**, the subsequent *anti*-addition of the halogen anion may follow two different ways: (i) the iodide, which is the larger anion, attacks the less hindered carbon, while (ii) bromide, the smaller one, is not affected by the bulky R group, and attacks the more reactive carbon (Scheme 7).

**Scheme 8:**
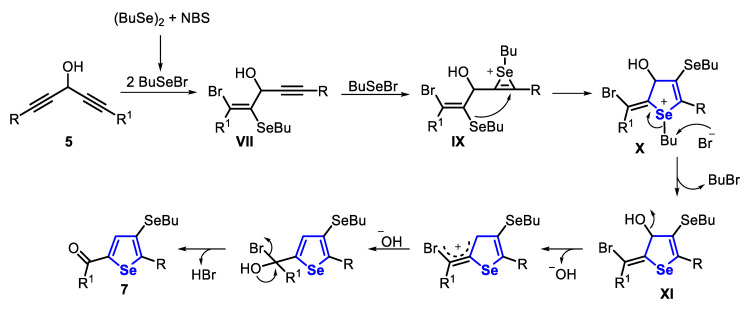
Proposed mechanism of the synthesis of 4-butylselanylselenophenes **7**.

Once the intermediate **VII** is formed, it reacts with BuSeBr, generated in situ, to form the seleniranium **IX**, which, after an intramolecular annulation, affords the cyclic selenonium **X**. In the sequence, the intermediate **XI** is formed by the elimination of BuBr. Finally, a 1,3-migration of the OH group is followed by the elimination of HBr, giving the 4-butylselanyl-selenophene **7** (Scheme 8).

**Scheme 9:**
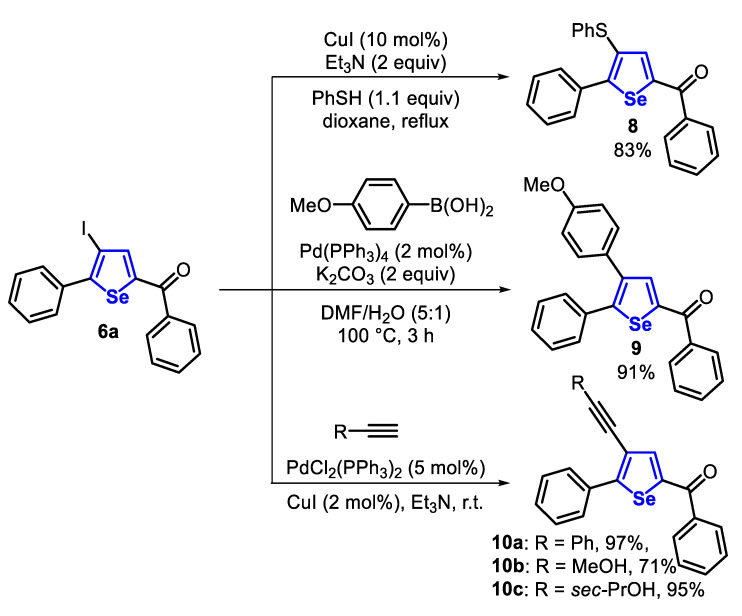
Cross-coupling reactions of 3-iodoselenophene **6a**.

The reactivity of the carbon-halogen bond of 4-iodo-selenophene **6** was explored in Pd-catalyzed cross-coupling reactions (Scheme 9). In this sense, 4-iodo-selenophene **6a** reacted with benzenethiol, under the Ullmann conditions, to access the 4-phenylthio-selenophene **8** in 83% yield. Additionally, **6a** was a suitable substrate in the Suzuki cross-coupling with arylboronic acid, affording 4-methoxyphenyl-selenophene **9** in 91% yield, after 3 h. Finally, selenophene **6a** was subjected to the Sonogashira cross-coupling with terminal acetylenes, giving the respective alkynyl selenophenes **10a**-**c** in good to excellent yields (Scheme 9).

**Scheme 10:**
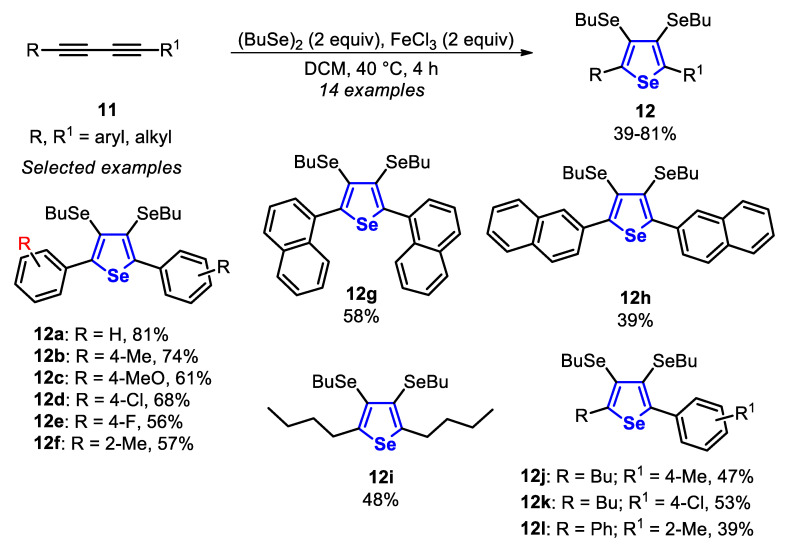
Reaction scope of the synthesis of 3,4-bis(butylselanyl)selenophenes **12**.

In 2015, the synthesis of 3,4-bis(butylselanyl)selenophenes **12** through the cyclization reaction between dibutyl diselenide and 1,3-diynes **11**, in the presence of equimolar amounts of FeCl_3_ in DCM, at 40 °C for 4 h, under Ar atmosphere was described (Scheme 10) [133]. In order to achieve better results, a 1:2 ratio of 1,3-diynes **11** and dibutyl diselenide was required, once 2 equivalents of dibutyl diselenide give 4 equivalents of the reactive species BuSe^−^. Among them, 3 equivalents are incorporated in the final product, while 1 equivalent acts as a nucleophile in an S_N_2 step, to remove the butyl group directly bonded to the selenium atom. The optimal conditions were applied to a wide range of electron-rich and electron-poor symmetrical 1,3-diynes **11**, affording the 3,4-bis(butylselanyl)selenophenes **12a**–**e** in moderate to good yields. The symmetrical 1,3-diynes **11**, substituted with *ortho*-methylaryl and naphthyl groups, reacted satisfactorily to afford the selenophenes **12f**–**h** in moderate yields. When the reaction was carried out using dodeca-5,7-diyne, the alkyl-substituted selenophene **12i** was obtained in 48% yield. In addition, the reactivity of unsymmetrical 1,3-diynes **11** was also evaluated, and the respective selenophenes **12j**–**l** were obtained in 39–53% yields (Scheme 10).

**Scheme 11:**
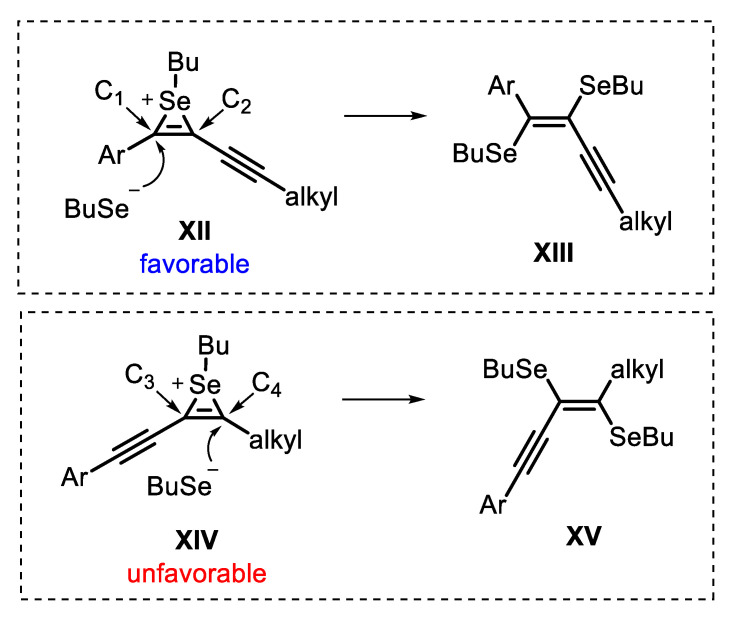
Key intermediates in the cyclization of unsymmetrical 1,3-diynes.

Regarding the regioselectivity of the reaction, the cyclization of unsymmetrical 1,3-diynes **11** can be led to two possible regioisomers. Thus, a detailed analysis of the key intermediates could be helpful to determine the exact position of each selenium atom, as well as which one performs the nucleophilic attack in the annulation step (Scheme 11). The first possibility is the nucleophilic *anti*-attack of BuSe^-^ at C1 in the seleniranium ion **XII**, giving the selenoenyne **XIII**. On the other hand, an *anti*-attack at C4 in the seleniranium ion **XIV**, affords selenoenyne **XV**. Once the aromatic π-system is richer in electrons than the C-C triple bond, the aromatic ring stabilizes the seleniranium ring, favoring the formation of the intermediate **XII** over **XIV**. Due to this effect, the reaction regioselectivity is achieved, passing by the selenoenyne **XIII**, which is converted into the respective selenophene **12** (Scheme 11).

**Scheme 12:**
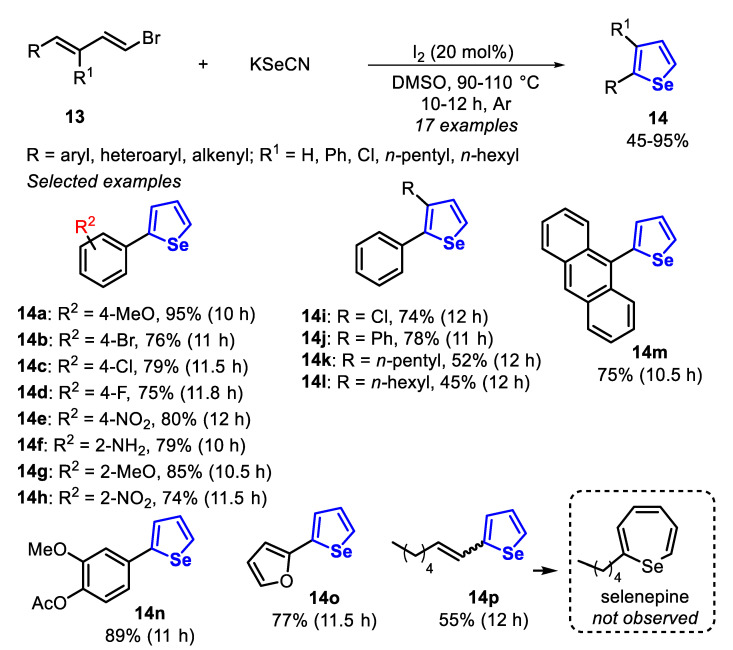
Scope of the iodine-catalyzed reaction of 1,3-dienyl bromides with KSeCN.

In 2017, the synthesis of selenophenes **14** by the reaction between 1,3-dienyl bromides **13** and potassium selenocyanate in the presence of I_2_ (20 mol%) as a benign, cheap and easily available catalyst was reported (Scheme 12) [134]. The reactions were carried out in DMSO, for 10–12 h under heating (90–100 °C) and argon atmosphere. The optimal conditions were extended to 1,3-dienyl bromides **13** bearing electron-donating (R^2^ = 4-MeO, 2-MeO) and electron-withdrawing groups (R^2^ = 4-F, 4-Cl, 4-Br, 4-NO_2_, 4-NH_2_, 2-NO_2_) in the aromatic ring, reaching the desired 2-arylselenophenes **14a**–**h** in 74% to 95% yield. Reactions between KSeCN and aryl-1,3-dienyl bromides **13** bearing different groups pendant to the vinyl carbon (R^1^ = Cl, Ph, *n-*pentyl and *n-*hexyl) reacted smoothly to afford the products **14i**–**l** in moderate to good yields. Additionally, anthracenyl- and furyl-1,3-dienyl bromide **13** derivatives were suitable substrates for the reaction, and the products **14m** and **14o** were obtained in 75% and 77% yields, respectively. The 4-[(1*E*,3*E*)-4-Bromobuta-1,3-dien-1-yl]-2-methoxyphenyl acetate **13** efficiently reacted with KSeCN to produce the selenophene **14n** in 89% yield. Finally, it is worth mentioning that the 1,3,5-trienyl bromide **13** furnished the 2-vinylselenophene **14p** at 55% yield, without the formation of selenepine (Scheme 12).

**Scheme 13:**
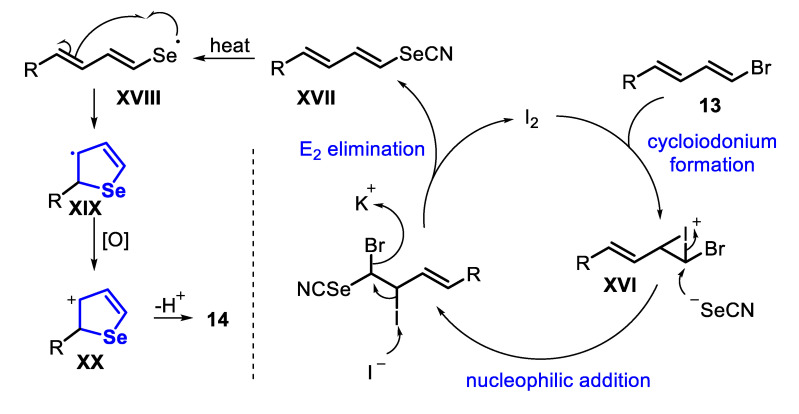
Proposed reaction pathway to prepare the selenophene **14**.

Control experiments suggested that the reaction mechanism involves the formation of the 1,3-dienylselenocyanate **XVII** as the key intermediate. Initially, 1,3-dienyl bromide **13** reacts with I_2_ to give a cycloiodonium **XVI**, which undergoes a nucleophilic addition of NCSe^-^, followed by an elimination step to give the 1,3-dienylselenocyanate **XVII** and regenerating I_2_. Thus, the intermediate **XVII** undergoes a homolytic cleavage of the Se-CN bond to give the Se-centered radical species **XVIII**. In the sequence, an intramolecular cyclization occurs by the reaction of the styryl C-C double bond, to give the five-membered cyclic intermediate **XIX**. The radical intermediate **XIX** is then oxidized to the carbocation **XX**, which is finally converted into the desired selenophene **14** through a proton elimination step (Scheme 13).

**Scheme 14:**
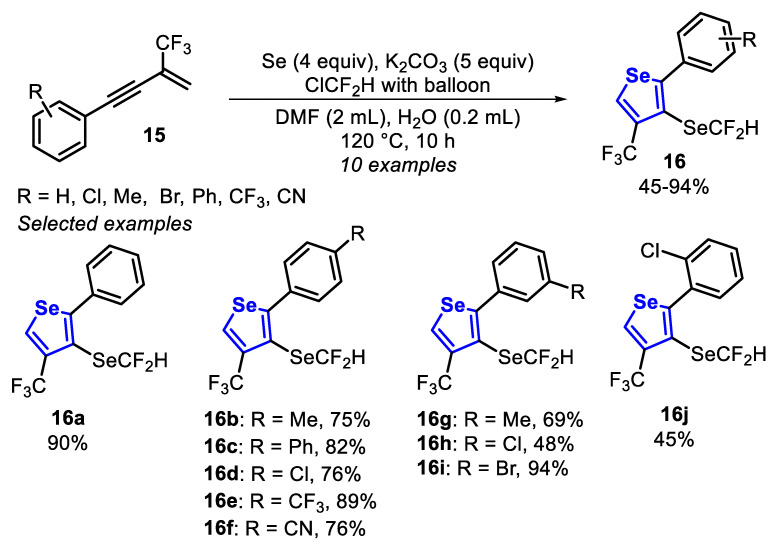
Scope of the synthesis of selenophenes from CF_3_-containing 1,3-enynes.

Recently, a divergent strategy was developed for the construction of 3-SeCF_2_H-4-CF_3_-selenophenes **16** from readily available CF_3_-containing 1,3-enynes **15** and elemental Se through a tandem selenophene construction/selective C3-selenation/difluoromethylselenation, under a ClCF_2_H atmosphere (in a balloon) in the presence of K_2_CO_3_ and DMF at 120 °C for 10 h (Scheme 14) [135]. In general, the reaction to access the products **16a**–**i** was efficient and robust once the substituents of the enyne **15** did not influence the reactivity. It is worth mentioning that steric effects were faced when the *ortho*-chlorophenyl pendant to the triple bond was employed, affording the expected product **16j** in 45% yield (Scheme 14).

### 2.2. Starting from Type B Precursors

**Scheme 15:**
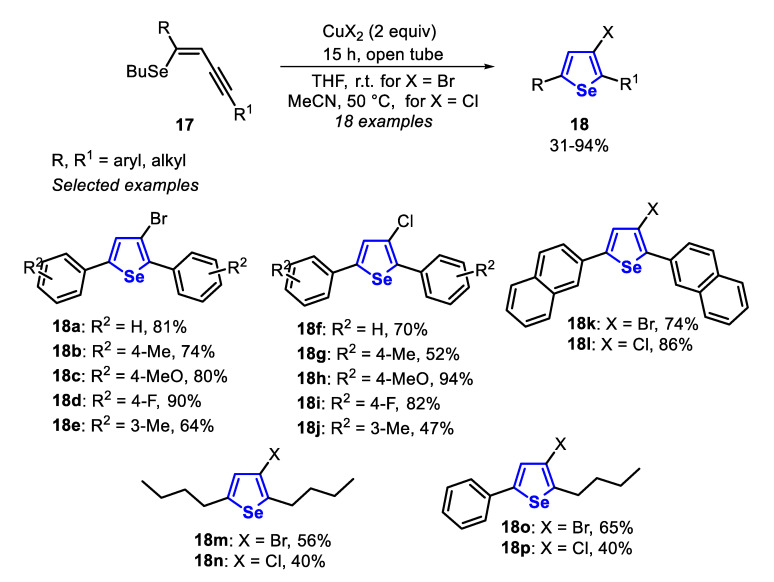
Cu-promoted cyclization of chalcogenoenynes to form 3-halo-chalcogenophenes.

In 2011, the intramolecular 5-*endo-dig* cyclization of (*Z*)-selenoenynes **17** mediated by copper(II) salts (CuBr_2_ at room temperature in THF, or CuCl_2_ at 50 °C in MeCN) to afford the corresponding 3-halochalcogenophenes **18** was reported (Scheme 15) [136]. Under these conditions, (*Z*)-selenoenynes **17,** substituted with neutral phenyl, electron-rich (R^2^ = 4-Me, 4-MeO, 3-Me) and electron-deficient (R^2^ = 4-F) aryl groups afforded the respective 3-haloselenophenes **18a**–**j** in moderate to good yields after 15 h. When bulky (*Z*)-selenoenyne was employed, the products **18k** and **18l** were obtained in 74% and 86% yields, respectively. The reaction was also tolerant to alkyl groups directly bonded to the alkyne, producing the desired 4-haloselenophenes **18m**–**p** in moderate yields (Scheme 15).

**Scheme 16:**
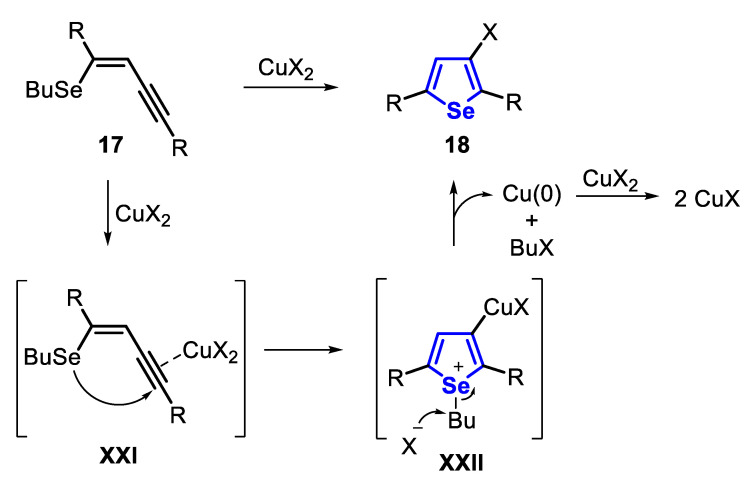
Mechanism of the Cu(II)-mediated intramolecular cyclization of (*Z*)-selenoenynes.

The proposed mechanism starts with the coordination of the C-C triple bond to the copper salt, generating the intermediate **XXI**. In the sequence, an intramolecular *anti*-attack of the selenium atom to the activated triple bond affords the selenonium salt **XXII**. After this, an S_N_2 displacement of the butyl group delivers the 3-haloselenophene derivative **18**, Cu(0) and 1-halobutane as a co-product. The formed Cu(0) can be oxidized by CuX_2_ to CuX, which explains the need for two equivalents of CuX_2_ in this reaction (Scheme 16).

**Scheme 17:**
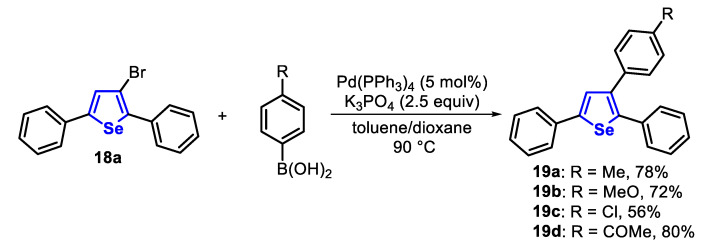
Synthesis of 2,3,5-triarylselenophenes by Pd-catalyzed Suzuki cross-coupling reaction.

In order to demonstrate the applicability of the synthesized compounds, 3-bromo-selenophene **18a** was subjected to the Pd-catalyzed Suzuki cross-coupling reaction with arylboronic acids. The reaction between the selenophene **18a** and electron-rich (R = Me, MeO) and deficient (R = Cl, COMe) arylboronic acids afforded the corresponding products **19a**–**d** in 56–80% yields (Scheme 17).

**Scheme 18:**
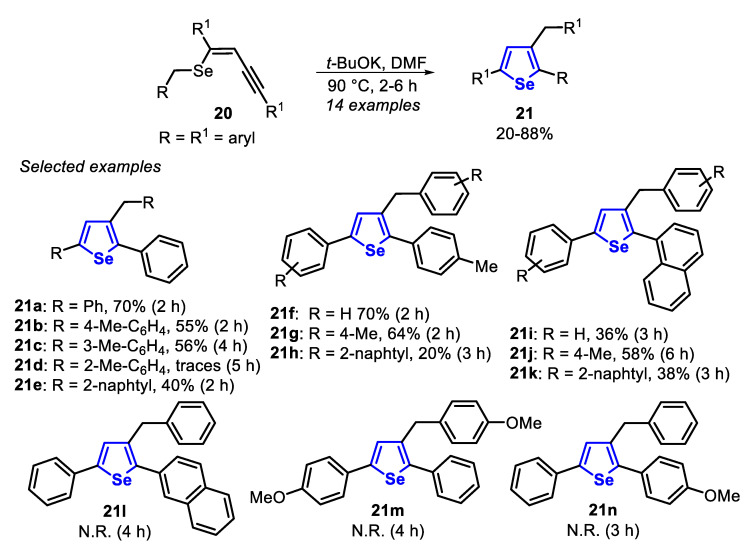
Synthesis of 3-benzyl-2,5-diarylselenophenes **21** via carbocyclization of (*Z*)-benzylselenoenynes **20**.

In the same year, the carbocyclization of (*Z*)-benzylselenoenynes **20**, using *t-*BuOK in DMF, at 90 °C for 2 to 6 h, to prepare the 3-benzyl-2,5-diarylselenophenes **21** (Scheme 18) was described [137]. Firstly, in order to evaluate the influence of the substituent at the pendant aromatic ring of the C-C triple bond (R^1^), the authors fixed the vinyl selenobenzyl group. The efficiency of the cyclization was significantly influenced by steric effects of the aromatic ring, affording the *ortho*-substituted selenophenes **21d** and **21e** in lower yields than the respective neutral, *para*- and *meta*-substituted ones, **21a**, **21b** and **21c**. In addition, when the (*Z*)-benzylselenoenynes **20,** bearing different substituents at the BnSe-vinyl groups, were used, the corresponding selenophenes **21f**-**l** were obtained in low to good yields. Limitations were found when the electron-donor MeO group was attached at the *para*-position of the pendant aromatic ring of the C-C triple bond (R^1^) or of the BnSe-vinyl group. In these cases, the desired selenophenes **21m** and **21n** were not formed (Scheme 18).

**Scheme 19:**
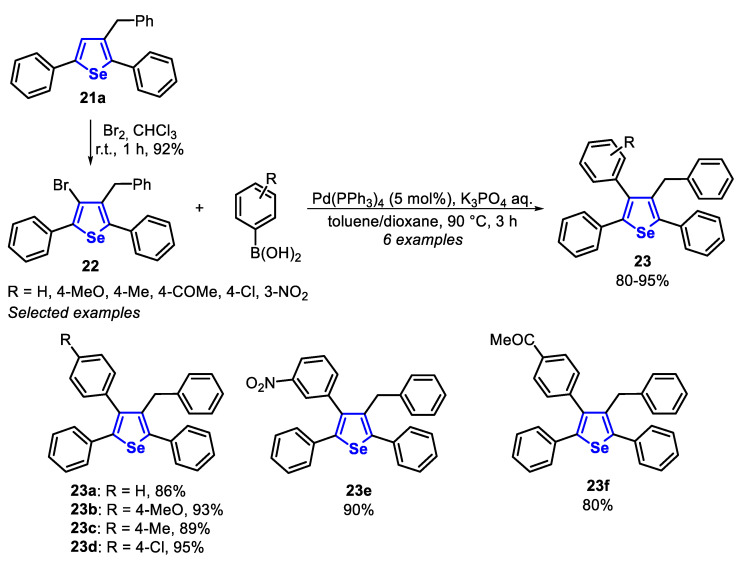
Palladium-catalyzed Suzuki cross-coupling reaction of selenophene **22**.

The synthetic versatility of the synthesized compounds was demonstrated in the Pd-catalyzed Suzuki reaction using different arylboronic acids. Initially, the selenophene **21a** was functionalized by the reaction with Br_2_ (2.5 equiv) in CHCl_3_, at room temperature, to afford the 4-Br substituted selenophene **22** in 92% yield. The functionalized selenophene **22** was then submitted to the reaction with different electron-rich and electron-deficient arylboronic acids in the presence of Pd(PPh_3_)_4_ (5 mol%) and an aqueous solution of K_3_PO_4_ (1 mmol), using a 1:1 mixture of dioxane/toluene as solvent, at 90 °C for 3 h. The reaction was not sensitive to electronic effect, and electron-rich and electron-poor arylboronic acids gave equally good yields of the respective selenophenes **23a**–**f** (Scheme 19).

**Scheme 20:**
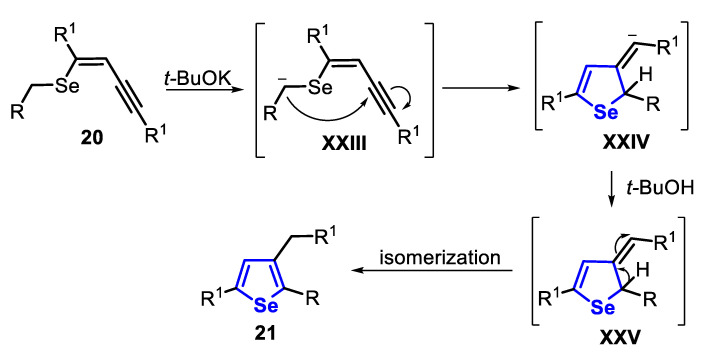
Mechanism of the carbocyclization of the (*Z*)-benzylselenoenynes **20**.

The proposed mechanism for the synthesis of the product **21** initiates with the deprotonation of the (*Z*)-benzylselenoenyne **20** by *t-*BuOK to generate the α-Se-stabilized carbanion **XXIII**, which undergoes an intramolecular cyclization to produce the vinyl carbanion intermediate **XXIV**. Thus, the intermediate **XXIV** is protonated, being converted to the intermediate **XXV**, which finally undergoes an isomerization reaction to afford the desired selenophene **21** (Scheme 20).

**Scheme 21:**
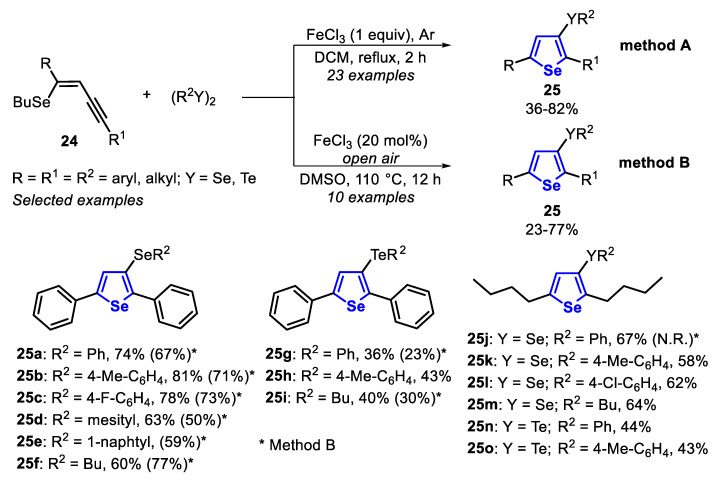
Scope of the synthesis of 3-organochalcogen selenophenes.

In 2012, a FeCl_3_/dichalcogenide-mediated intramolecular cyclization of (*Z*)-selenoenynes **24** for the synthesis of 2,5-disubstituted-3-(organoseleno)-selenophenes **25** [24] was reported through two different methods: using one equivalent or a catalytic amount (20 mol%) of FeCl_3_. In method **A**, using a stoichiometric amount of FeCl_3_, the (*Z*)-selenoenynes **24** reacted with diorganyl dichalcogenide (0.55 equiv), in DCM at 40 °C, under argon atmosphere, while in method **B** (20 mol% of FeCl_3_) the reaction was conducted in DMSO at 110 °C in an open flask condition (Scheme 21). The protocol has proved to be robust and general, affording 33 different derivatives of the 2,5-disubstituted-3-(organoseleno)-selenophenes **25**. No significant differences were found between electron-rich and electron-deficient diorganyl dichalcogenides, and the selenophenes **25a**–**c** and **25g**–**h** were accessed in moderate to good yields. However, the reaction efficiency was hampered when diorganyl diselenides bearing sterically hindered groups (R^2^ = mesityl or 1-naphthyl) were employed, giving the selenophenes **25d** and **25e** in moderate yields, using both methods. Regarding the dichalcogenide reaction partner, the results suggest that electronic effect did not influence the reaction directly; however, the transformation is sensitive to steric effects. Additionally, dialkyl diselenides and ditellurides were satisfactorily employed as a substrate, giving the products **25f** and **25i** in acceptable yields. It is worth mentioning that, in general, the results reached with diorganyl diselenides are consistently better than those obtained with ditellurides. This trend is intricately linked to the fact that tellurides are more susceptible to undergoing a telluroxide elimination reaction than the corresponding selenides. The protocols were successfully applied to alkyl selenoenynes **24**, in the presence of a wide range of functionalized diorganyl dichalcogenides, giving the respective selenophenes **25j**–**o** in moderate to good yields (Scheme 21).

**Scheme 22:**
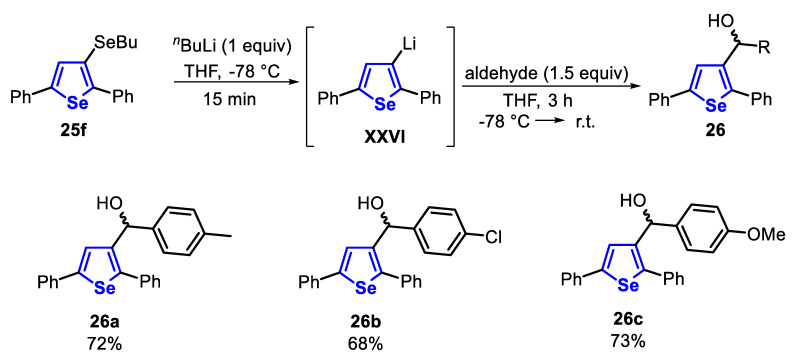
Reactions of 3-lithio-selenophene with aldehydes.

The reactivity of the 2,5-disubstituted-3-(organoseleno)-selenophenes **25** was explored in the transmetalation Li/Se. Thus, the selenophene **25j** reacted with butyllithium (1 equiv) in THF at −78 °C, affording the Li-containing species **XXVI**. After this, the species **XXVI** was trapped with aldehydes (1.5 equiv) at −78 °C and the system was allowed to reach the room temperature. The secondary alcohols **26a**-**c** were satisfactorily obtained in 68–73% yields after 3 h (Scheme 22).

**Scheme 23:**
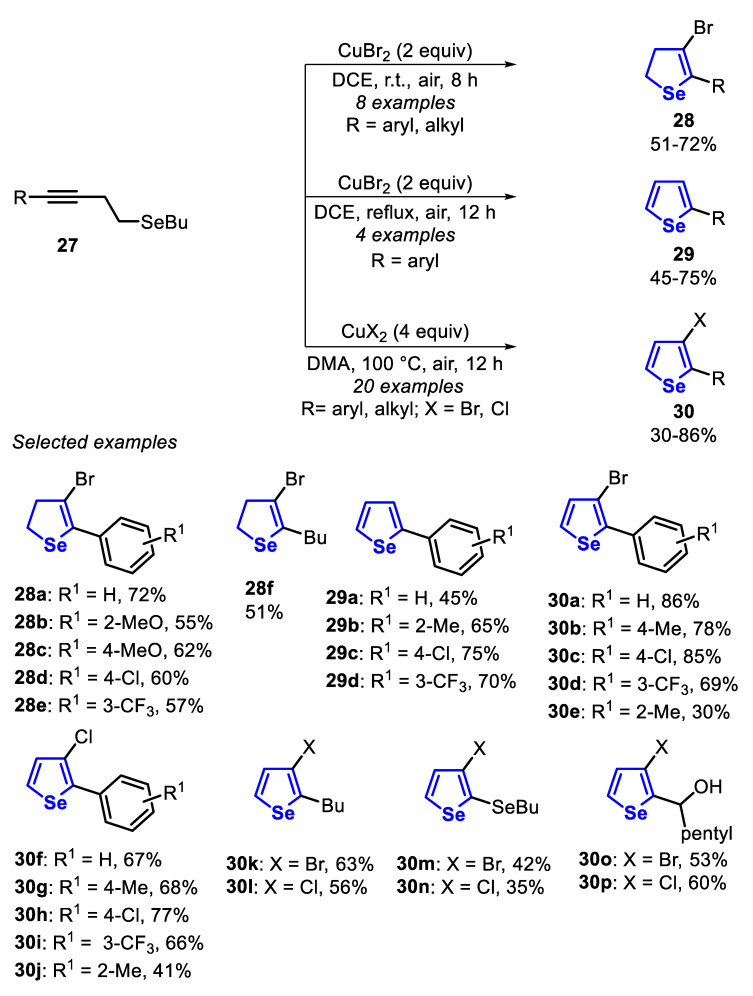
Scope of the Cu(II)/halide-mediated cyclization of homopropargyl selenides.

In 2013, the Cu(II)/halide-mediated cyclization of homopropargyl selenides **27**, to give selectively 2,3-dihydroselenophenes **28**, 2-arylselenophenes **29** and 3-haloselenophenes **30** was reported (Scheme 23) [138]. The selectivity of the reaction was achieved by controlling the solvent and the reaction temperature. Thus, using CuBr_2_ (2 equiv) and 1,2-dichloroethane, at room temperature under air atmosphere, the 4-bromodihydroselenophenes **28** were obtained after 8 h. Electron-rich and electron-deficient homopropargyl selenides **27** reacted smoothly to produce the 3-bromodihydroselenophenes **28a**–**e** in moderate yields. Intriguingly, when the *ortho*-alkynylanisole selenide **27** was used, the dihydroselenophene **28b** was exclusively obtained, and the benzo[*b*]furan derivative, a possible by-product, was not detected. This remarkable selectivity may be attributed to the electronic effect (relative nucleophilicity of the Se atom and the cationic nature of the intermediate), as well as to the resistance of the methoxyl group to undergo a demethylation, followed by a ring closure. The dihydroselenophene **28f**, derived from the homopropargyl selenide bearing alkyl group (R = Bu), was obtained at a lower yield of 51%. When the reaction was conducted at a reflux instead of at room temperature, 2-arylselenophenes **29** were obtained. Neutral- and electron-rich homopropargyl selenides **27** (R^1^ = H and 2-Me) afforded the products **29a** and **29b** in 45% and 65% yield, respectively, while electron-deficient substrates (R^1^ = 4-Cl and 3-CF_3_) were more efficient, delivering the products **29c** and **29d** in 75% and 70% yield, respectively (Scheme 23).

When the reaction was performed using dimethylacetamide (DMA) as a solvent at 100 °C, the reaction selectivity was changed and 3-halo-selenophenes **30** were exclusively obtained. It is worth mentioning that this protocol is not influenced by the electronic effect and the selenophenes **30a**–**d**, **f**–**i** were obtained in moderate to very good yields. However, the steric hindrance affected the reactivity, and a remarkable decrease in the efficiency was observed when the *ortho*-Me substituent was present, and the products **30e** and **30j** were obtained in 30% and 41% yields, respectively. This optimized condition was suitable for differently substituted homopropargyl selenides **27** (R = Bu, BuSe and propargyl alcohol), affording the respective 3-bromo- and 4-chloroselenophenes **30k**-**p** in poor to moderate yields (Scheme 23).

**Scheme 24:**
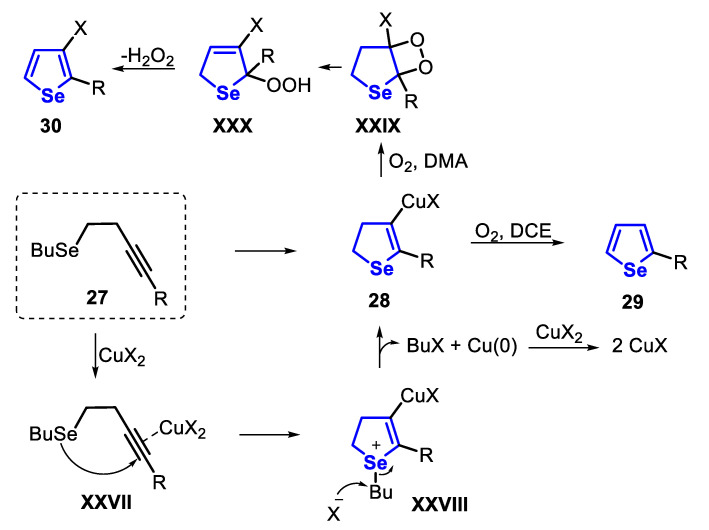
Mechanism of the Cu(II) halide-mediated cyclization of homopropargyl selenides **27**.

The proposed mechanism of the cyclization reaction of the homopropargyl selenides **27** starts with the coordination between the Cu catalyst and the C-C triple bond generates the intermediate **XXVII**, which undergoes an *anti*-attack of the Se to the activated triple bond, to produce the selenonium intermediate **XXVIII**. Finally, a reductive elimination yields the 3-halodihydroselenophene derivative **28**, which, in the presence of atmospheric oxygen, can be oxidized to the selenophene **29**. The Cu(0) species reacts with CuX_2_ spontaneously to produce two equivalents of CuX. On other hand, the synthesis of the 3-haloselenophenes **30** is performed in the presence of DMA at 100 °C under air atmosphere via an oxygen-promoted oxidation reaction of the dihydroselenophene **28**, through hydroperoxide intermediates **XXIX** and **XXX** (Scheme 24).

**Scheme 25:**
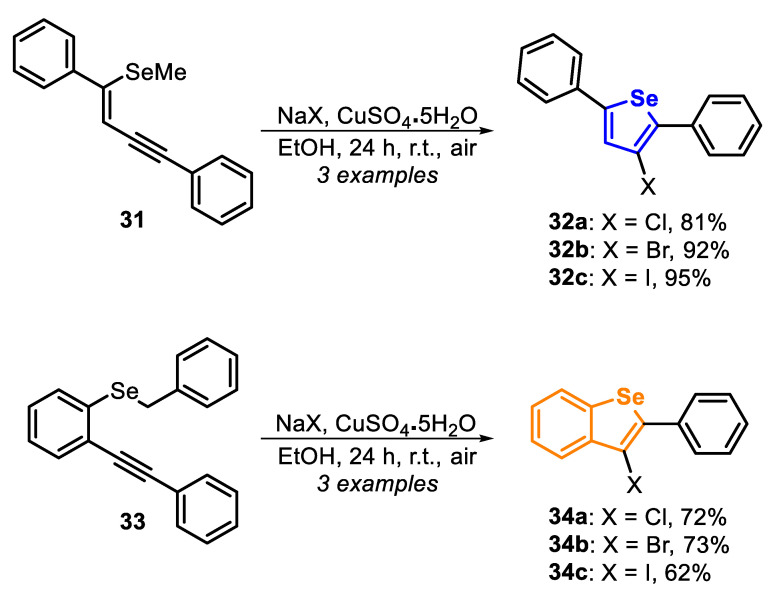
Reaction scope of the synthesis of halogenated selenophenes **32** and benzo[*b*]selenophenes **34**.

In 2017, the green synthesis of halogenated selenophenes **32** and benzo[*b*]selenophenes **34** through the electrophilic cyclization of selenoenyne **31** and selenoalkyne **33**, using sodium halides as electrophilic reaction partner, in EtOH, at room temperature and under air atmosphere was described [139]. When the enyne **31** was submitted to the optimized condition, the desired chloro-, bromo-, and iodoselenophenes **32a**–**c** were satisfactorily accessed in 81%, 92% and 95% yields, respectively. The authors claimed that this was the first Cl-promoted cyclization of the 2-alkynylmethylselenobenzene **33**, affording the respective 3-chlorobenzo[*b*]selenophene **34a** in 72% yield. In addition, this protocol was extended to the synthesis of the 3-bromobenzo[*b*]selenophene **34b** and 3-iodobenzo[*b*]selenophene **34c** in 73% and 62% yield, by using NaBr and NaI, respectively (Scheme 25).

**Scheme 26:**
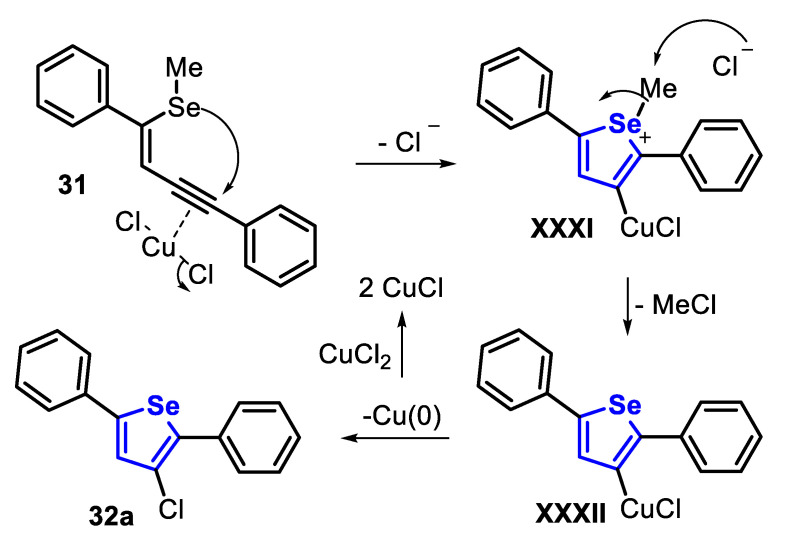
Proposed mechanism of the synthesis of chloroselenophene **32a**.

The first step in the synthesis of chloroselenophene **32a** is the in situ generation of CuCl_2_, by the reaction between Cu_2_SO_4_·5H_2_O and NaCl. Then, the resulting CuCl_2_ weakly coordinates to the C-C triple bond of selenoenyne **31**, which undergoes an intramolecular *anti*-attack from the nearby Se nucleophile, to afford the selenonium intermediate **XXXI**. The methyl group can be subsequently removed by an S_N_2 displacement, promoted by the chloride anion, to form the intermediate **XXXII**. Finally, a reductive elimination leads to the desired product **32a**. The resulting Cu(0) is easily oxidized by CuCl_2_ to produce CuCl (Scheme 26). Similarly, Cu_2_SO_4_·5H_2_O in the presence of NaI or NaBr results in the formation of CuI_2_ or CuBr_2_, respectively, which are converted in situ to I_2_ and Br_2_. The mechanism of the cyclization involving I_2_ and Br_2_ electrophiles is well established [140].

**Scheme 27:**
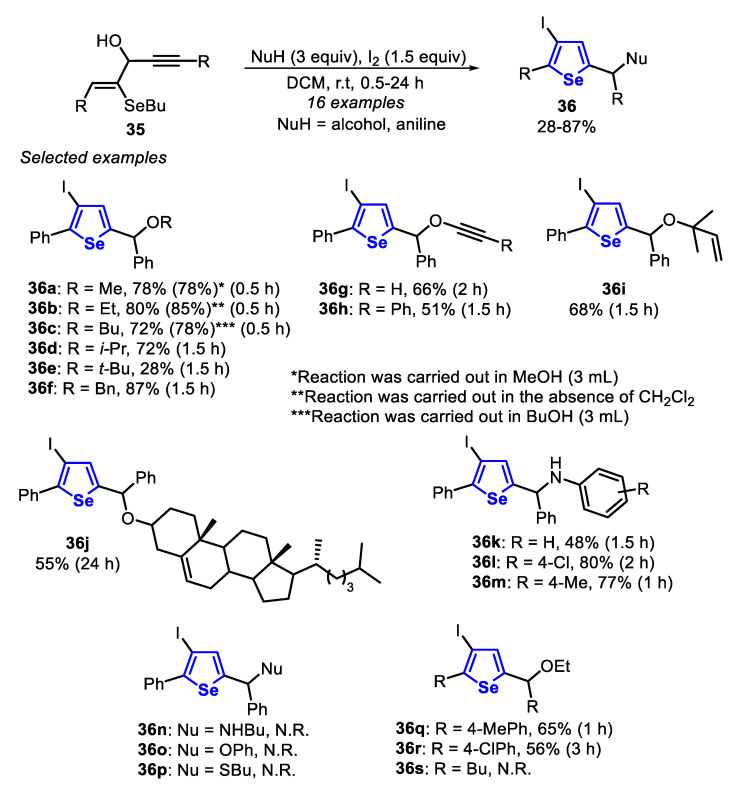
Scope and limitations of the synthesis of 3-iodoselenophenes **36**.

**Scheme 28:**
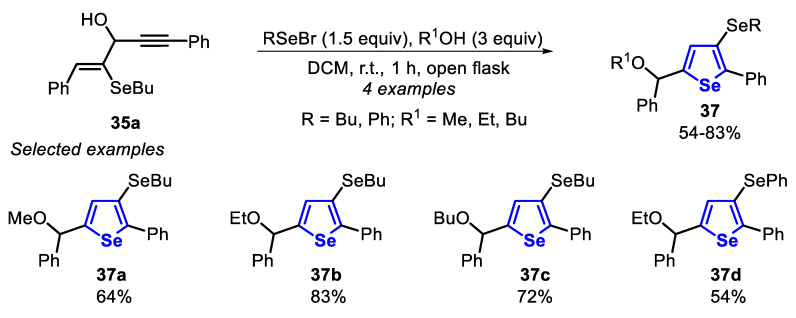
Scope of synthesis of 3-organoselanyl-selenophenes **37**.

In 2017, the I_2_-promoted electrophilic cyclization of selenoenynes **35**, in the presence of an appropriate nucleophile, to give the 3-iodo-selenophenes **36** and the 3-organoselanyl-selenophenes **37** was described (Scheme 27 and Scheme 28) [141]. The optimized condition to prepare the 3-iodo-selenophenes **36** involves the addition of I_2_ (1.5 equiv) and a nucleophile (3 equiv) to a solution of selenoenynes **35** in DCM. The resulting mixture was stirred at room temperature and in an open flask for 0.5 to 24 h. The behavior of different nucleophiles was evaluated, causing the selenoenyne **35** to react with a range of alcohols and amines. Thus, when primary alcohols (NuH = MeOH, EtOH and BuOH) were employed, the respective selenophenes **36a**-**c** were obtained in 78–85% yields, after just a half hour. In contrast, the use of *i*-PrOH and *t-*BuOH resulted in the formation of products **36d** and **36e** in 72% and 28% yield, respectively, after 1.5 h. Satisfactorily, benzyl alcohol, alkynols and allyl alcohols were successful employed as nucleophiles, giving the corresponding selenophenes **36f**-**i** in 51–87% yields, after 1.5 to 2 h. Cholesterol was also a suitable nucleophile, giving the respective selenophene **36j** in 55% yield after 24 h. Aniline derivatives were suitable substrates in the reaction with selenoenyne **35**, with *p*-chloro and *p*-toluidine giving the respective selenophenes **36l** and **36m** in 80% and 77% yields, respectively. In contrast, unsubstituted aniline afforded the respective selenophene **36k** in just 48% yield. Additionally, the effect of the presence of electron-donor and electron-withdrawing groups in the pendant phenyl of the selenoenynes **35** was evaluated in the reaction with EtOH, and the selenophenes **36q** and **36r** were obtained in 65% and 56% yields, respectively. No reaction was observed using alkyl amine, phenol and thiols as nucleophiles, or alkyl-substituted selenoenyne (R = Bu) as substrates. The authors claimed that, in these cases, the selenoenynes were totally consumed, producing a complex mixture of products (Scheme 27).

In the same work, 3-organoselanyl-selenophenes **37** were accessed through the reaction between selenoenyne **35a**, alcohols (3 equiv) and organoselenyl bromide (1.5 equiv), generated in situ. For instance, the product **37a** was obtained in 64% yield by the addition of BuSeBr, freshly prepared in situ by the reaction of *N*-bromosuccinimide (1.5 equiv) with BuSeSeBu (1.5 equiv), to a solution of selenoenyne **35a** in MeOH. EtOH and BuOH were also satisfactorily employed as nucleophiles, giving the selenophenes **37b** and **37c** in 83% and 72% yields, respectively. The less reactive electrophile PhSeBr was a suitable substrate, promoting the cyclization of the selenoenyne **35a** to the selenophene **37d** in 54% yield (Scheme 28).

**Scheme 29:**
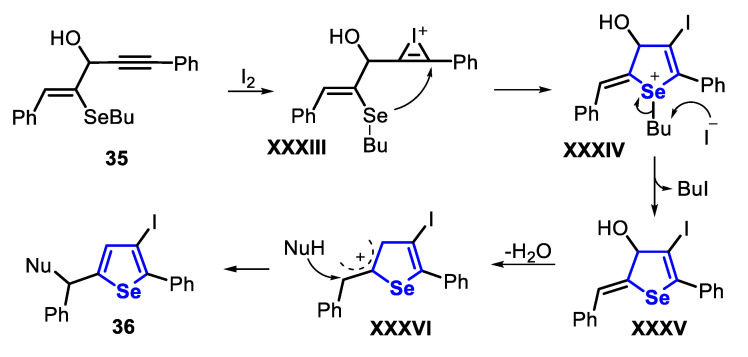
Proposed mechanism of the synthesis of 3-iodoselenophenes **36** from **35**.

The proposed mechanism for the synthesis of 3-iodoselenophenes **36** involves the initial formation of an iodonium intermediate **XXXIII**, followed by a regioselective 5-*endo*-*dig* intramolecular nucleophilic attack of the selenium atom to the C-C triple bond, giving the selenonium intermediate **XXXIV**. The removal of the butyl group via an S_N_2 displacement, promoted by the iodide anion, affords the dihydroselenophene **XXXV** and BuI as a byproduct. The aromatization of dihydroselenophene **XXXV** leads to allylic cation **XXXVI**, which is trapped by a nitrogen- or/and oxygen-based nucleophile, producing the desired selenophene **36** (Scheme 29).

**Scheme 30:**
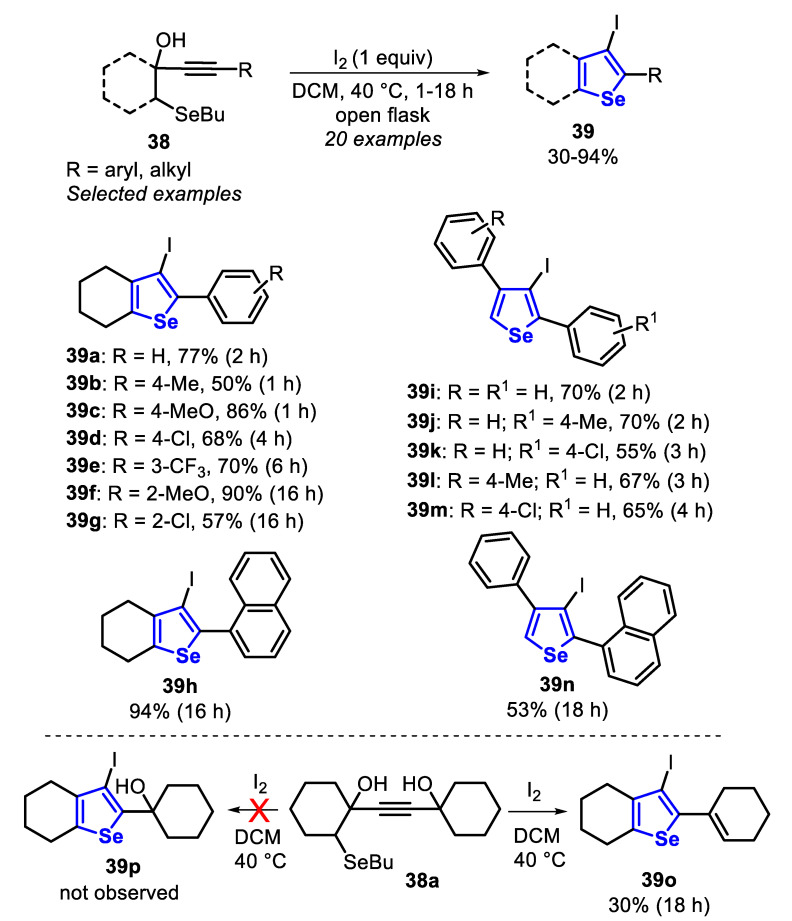
Scope of the synthesis of 3-iodoselenophenes **39**.

**Scheme 31:**
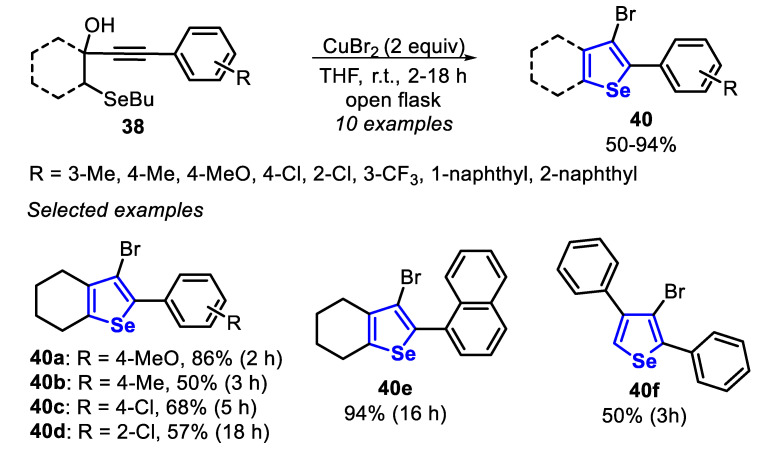
Scope of the synthesis of 3-bromo-selenophenes **40**.

**Scheme 32:**
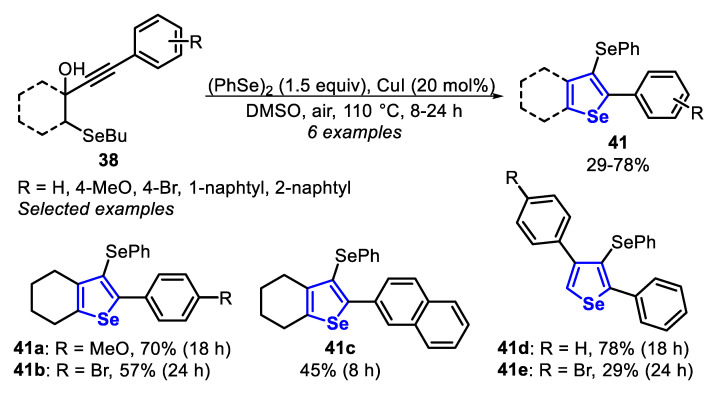
Scope of the synthesis of 3-(phenylselanyl)selenophenes **41**.

In 2018, the I_2_-promoted electrophilic cyclization of butylselanyl propargyl alcohols **38**, for the synthesis of 3-substituted selenophenes **39**–**41** was described (Scheme 30, Scheme 31 and Scheme 32) [142]. The 3-iodoselenophenes **39** were obtained by the reaction between butylselanyl propargyl alcohols **38** and I_2_ (1 equiv) in DCM under air atmosphere at 40 °C for 1 to 18 h (Scheme 30). The effect of different substituents in the phenyl group directly attached to the C-C triple bond of the butylselanyl propargyl alcohols **38** was examined. The presence of electron-donor groups at the *para*-position positively influenced the reaction, and selenophene **39b** (R = 4-MePh) and **39c** (R = 4-MeOPh) were obtained in 50% and 86% yield after 1 h. The electron-withdrawing groups, however, caused a decrease in the reactivity, and the compounds **39d** (R = 4-ClPh) and **39e** (R = 3-CF_3_Ph) were obtained in 68% and 70% yield after 4 and 6 h of reaction, respectively. Sterically hindered butylselanyl propargyl alcohols **38** (R = 2-MeOPh, 2-ClPh and 1-naphthyl) were able to give the desired selenophenes **39f**-**h** in moderate to good yields after longer reaction times (16 h), suggesting that the steric effects are more prominent than the electronic effect. Substrates bearing electron-rich and electron-deficient aryl substituents at the propargyl position provided the corresponding selenophenes **39i**-**m** in 55% to 70% yields, after 2 to 4 h. The sterically hindered selenophene **39n** was obtained in 53% yield after 18 h of reaction. The reaction of the alkynyl diol **38a** under the optimal conditions afforded the elimination product cyclohexenylselenophene **39o** instead the expected alcohol **39p**. This event is promoted by iodine, which, besides acting to promote the selenophene core aromatization, also promotes the C-C double bond formation in cyclohexane, via an eliminative dehydration process (Scheme 30).

Regarding the formation of the 3-bromo-selenophenes **40**, Br_2_ and NBS were not efficient in promoting the cyclization of the substrate **38** in an efficient way. However, CuBr_2_ (2 equiv) smoothly promoted the formation of the selenophenes **40a**-**f** in 50% to 94% yields, after 2 to 18 h, in the presence THF at room temperature and under air atmosphere. Similar to what was previously observed in the reaction with iodine (Scheme 30), electron-rich and electron-deficient butylselanyl propargyl alcohols **38** afforded the 3-bromo-selenophenes **40a**-**c** in moderate to very good yields, after 2–5 h of reaction. Additionally, sterically hindered butylselanyl propargyl alcohols **38** (R = 2-Cl and 1-naphthyl) reacted satisfactorily, affording the products **40d** and **40e** in 57% and 94% yields, after 18 and 16 h (Scheme 31).

Finally, the 3-(phenylselanyl)selenophenes **41** were obtained through the CuI-catalyzed reaction between butylselanyl propargyl alcohols **38** and diphenyl diselenide (1.5 equiv), in DMSO as solvent at 110 °C and under an air atmosphere (Scheme 32). Under these conditions, six 3-(phenylselanyl)selenophenes were prepared in poor to acceptable yields, after 8–24 h of reaction. When the aryl group attached to the C-C triple bond was substituted with a methoxyl group (R = 4-MeO) a better yield was obtained in comparison to that substituted with a bromine group (R = 4-Br) (**41a** vs. **41b**). This difference in the reactivity is due to the electron density increasing, caused by the mesomeric effect (R = 4-MeO), favoring the formation of a Cu-π-complex intermediate. In contrast, when the reaction was carried out with butylselanyl propargyl alcohol **38,** bearing a naphthyl group (R = 2-naphthyl), the respective selenophene **41c** was obtained in only 45% yield after 8 h, due to the steric hinderance around the C-C triple bond. When a neutral phenyl group was employed, the corresponding selenophene **41d** was obtained in good yield, while the presence of the *para*-substituted aryl group at the propargyl position negatively affected the reaction (Scheme 32).

**Scheme 33:**
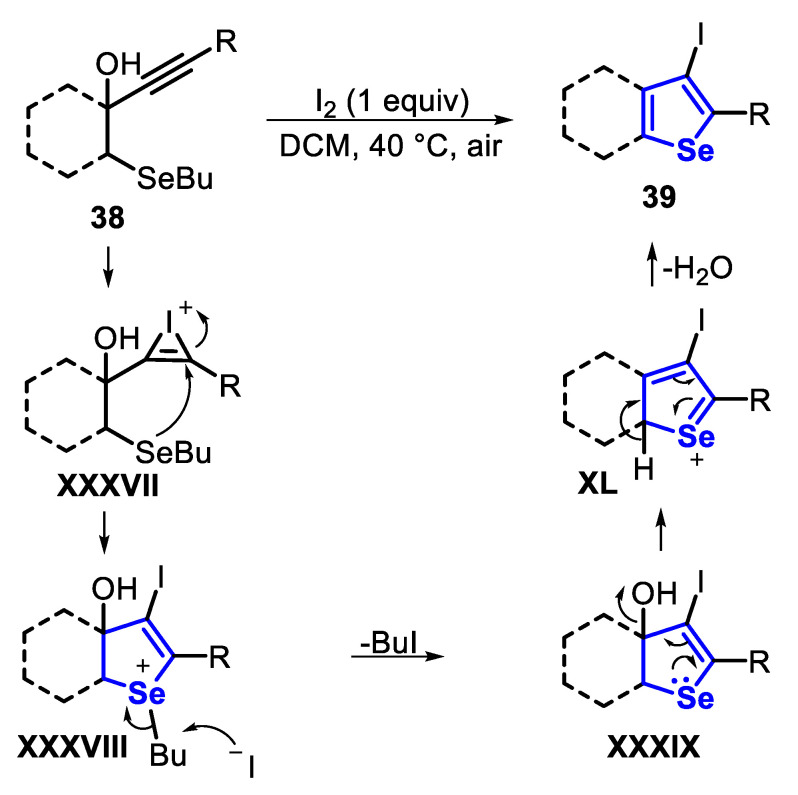
Mechanism of the synthesis of 3-iodoselenophenes **39**.

In order to collect data on the identity of the intermediates and then proposing a mechanism, the reaction for the synthesis of 3-iodo-selenophene **39** was monitored by ^1^H NMR and mass spectrometry. Based on these data, it was suggested that the cyclization and removing of the butyl group from the selenonium species occur before the aromatization step. Thus, initially, I_2_ adds to the C-C triple bond to give the iodonium intermediate **XXXVII**. The intramolecular nucleophilic *anti*-attack of Se to C1 produces the 2,3-dihydroselenophene selenonium salt **XXXVIII**. Thus, the iodide anion promotes butyl group removal, through an S_N_2 displacement, giving the 2,3-dihydroselenophene **XXXIX**, which undergoes an aromatization step to afford the 3-iodo-selenophene **39** (Scheme 33).

**Scheme 34:**
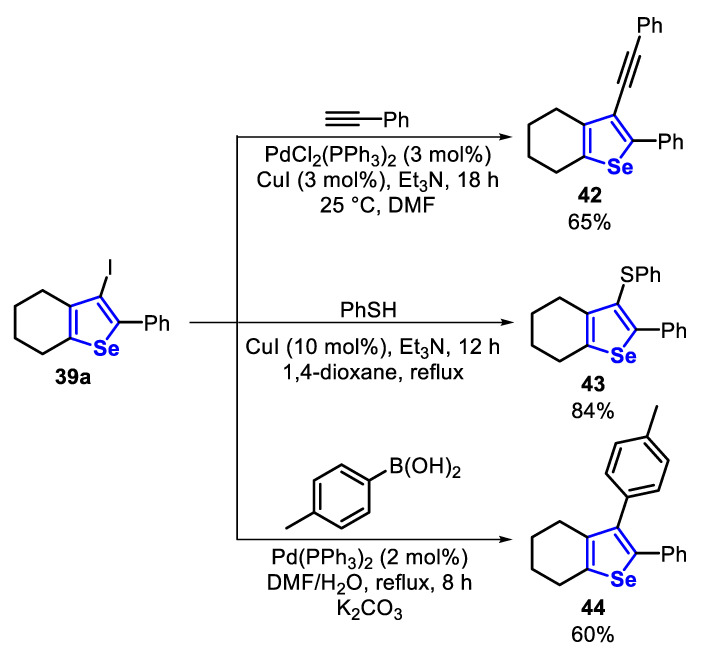
Cross-coupling reactions using 3-iodoselenophene **39a**.

The prepared 3-iodoselenophenes **39** were used as starting materials for the synthesis of multifunctional selenophenes, through Cu- and Pd-catalyzed Sonogashira, Ullmann and Suzuki cross-coupling reactions (Scheme 34). Thus, under the Sonogashira condition, 3-iodoselenophe **39a** reacted with phenylacetylene, at room temperature, in the presence of PdCl_2_(PPh_3_)_2_ (3 mol%), CuI (3 mol%), Et_3_N and DMF to give the 3-alkynylselenophene **42** in 65% yield after 12 h. Additionally, under the Ullmann protocol, **39a** reacted with benzenethiol in the presence of CuI (10 mol%), Et_3_N and 1,4-dioxane under reflux to afford the 3-thiophenylselenophene **43** in 84% yield, after 18 h. Finally, under Suzuki conditions, 2-arylselenophene **44** was obtained in 60% yield, through the reaction between **39a** and 4-tolylboronic acid, in the presence of Pd(PPh)_3_ (2 mol%) as the catalyst, K_2_CO_3_ as the base and a mixture of DMF/H_2_O as the solvent under reflux (Scheme 34).

**Scheme 35:**
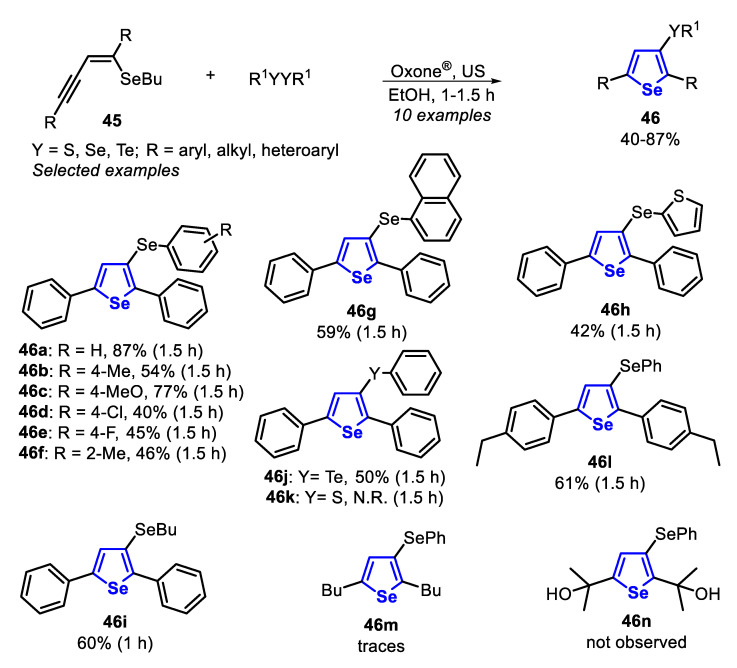
Scope and limitations of the synthesis of 3-selanylselenophenes **46**.

Very recently, some of us [143] reported the electrophilic cyclization of (*Z*)-selenoenynes **45** under ultrasound irradiation (US) conditions, promoted by the system R^1^YYR^1^/Oxone^®^/ethanol (Y = Se, Te), to access 3-selanyl/telanylselenophenes **46** (Scheme 35). This green alternative was carried out using Oxone^®^ as an inexpensive and non-toxic oxidant agent to promote the oxidative cleavage of Se-Se and Te-Te bonds. In order to evaluate the generality and limitations of this protocol, the (*Z*)-butyl(1,4-diphenylbut-1-en-3-yn-1-yl)selane **45a** (R = Ph) was reacted with several diorganyl diselenides. Electron-rich and electron-deficient diaryl diselenides were suitable reagents, affording the corresponding selenophenes **46a**-**f** in moderate to good yields, after 1.5 h. In these cases, the presence of substituents at the *para*-position of the aryl ring reduced the reactivity of the diselenide (**46a** vs. **46b**-**e**). This decreasing in the reactivity was less pronounced in the case of electron-rich diselenides, and the selenophenes **46b** (R = 4-Me) and **46c** (R = 4-MeO) were obtained in 54% and 77% yields, respectively. The electron-deficient (R = 4-Cl and 4-F) and *ortho*-tolyl (R = 2-Me) diselenides were less reactive, giving the respective selenophenes **46d**-**f** in 40%, 45% and 46% yields, respectively. This protocol was also compatible with aromatic bulky, heteroaromatic and aliphatic diselenides, affording the selenophenes **46g**-**i** in 42–60% yields. In addition, when diphenyl ditelluride was submitted to the optimal conditions, the corresponding *Te*-functionalized selenophene **46j** was obtained in 50% yield. Regarding the enyne counterpart, good result was obtained in the cyclization of the aryl substituted (*Z*)-enyne **45b** (R = 4-EtPh) using PhSeSePh, which was converted to the respective selenophene **46l**, in 61% yield. The reaction failed when diphenyl disulfide was the chalcogen source (**46k**), as well as starting from the (*Z*)-selenoenynes bearing diol (**46n**) and aliphatic groups (**46m**) as substrates (Scheme 35).

**Scheme 36:**
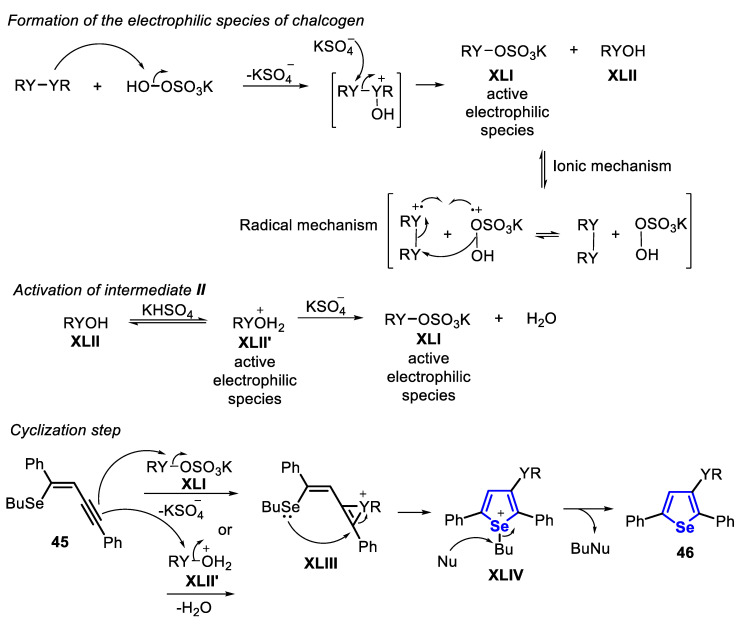
Proposed mechanism for the synthesis of 3-selanylselenophenes **46**.

The proposed mechanism of the reaction starts with the US-promoted oxidative cleavage of the Y-Y bond of the diorganyl dichalcogenide in the presence peroxymonosulfate (KHSO_5_), the active component of Oxone^®^, to give the reactive intermediates **XLI** and **XLII**. Once formed, intermediate **XLII** is activated by the acidic medium to form the strongest electrophile **XLII’**. Then, the selenoenyne **45** reacts with **XLI** or **XLII’** to form the seleniranium intermediate **XLIII**, that after an intramolecular attack by the electron pair of Se, gives the selenonium intermediate **XLIV**. Finally, **XLIV** undergoes a nucleophilic attack by one of the nucleophilic species present in the medium (SO_4_^2−^, HSO_4_^−^) to form the desired selenophene **46** (Scheme 36).

## 3. Synthesis of Benzoselenophenes

### 3.1. Starting from Type C Precursors

There are several protocols to obtain benzoselenophenes through intra- or intermolecular reactions between functionalized arenes and alkynes. Among them, electrophilic and radical cyclizations of type C precursors are the most common approaches, with modifications in the electrophilic source and the reaction conditions.

**Scheme 37:**
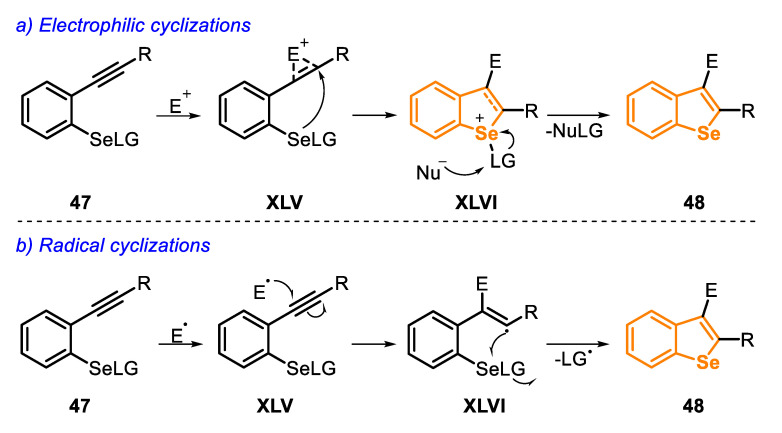
Electrophilic (**a**) and radical (**b**) cyclization processes starting from type C precursors.

In general, these reactions involve the addition of an electrophile to a C(*sp*) bond of *Se*-functionalized arenes, bearing an *ortho*-alkynyl group, such as compound **47** (type C precursor). In the electrophilic cyclization, there is initially a coordination of the electrophile (E^+^) to the C-C triple bond, generating the three-membered cyclic intermediate **XLV** (activation step). The selenium *anti*-attack takes place on the intermediate **XLV** (via a 5-*endo*-*dig* cyclization) to produce the intermediate **XLVI**. Finally, the leaving group attached to the *Se* atom is removed by a nucleophile (Nu^−^), present in the reaction mixture, via an S_N_2 path, affording the cyclized heterocyclic product **48** (Scheme 37a). On the other hand, in the radical cyclization reactions, the first step is the generation of the radical species, followed by an attack on the C(*sp*) bond in the type C precursor. Finally, the vinyl radical **XLVI** undergoes an intramolecular cyclization (a 5-*exo*-*trig* cyclization), followed by the elimination of the LG radical, to generate the desired benzoselenophene **48** (Scheme 37b).

**Scheme 38:**
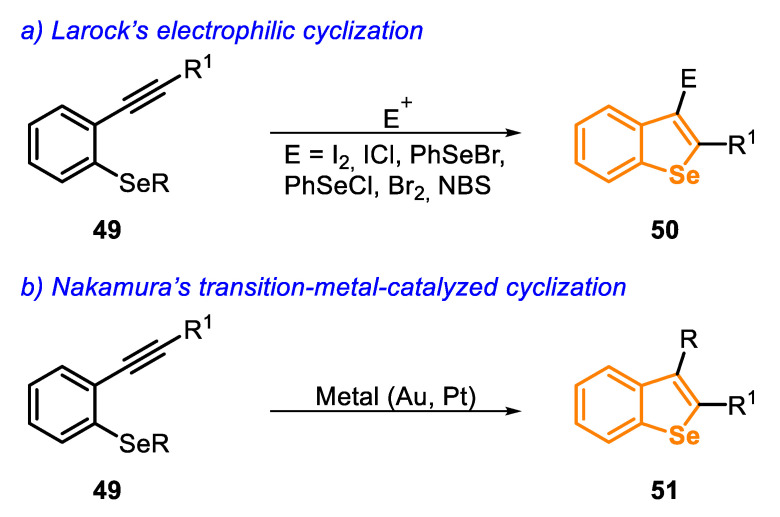
Larock’s (**a**) and Nakamura’s (**b**) protocols to prepare benzoselenophenes.

In the early 2000s, Larock’s and Nakamura’s groups [144,145,146] described valuable protocols for the synthesis of benzo[*b*]selenophenes, starting from type C precursors, using different electrophilic sources or transition-metal-catalyzed strategies (Scheme 38).

**Scheme 39:**
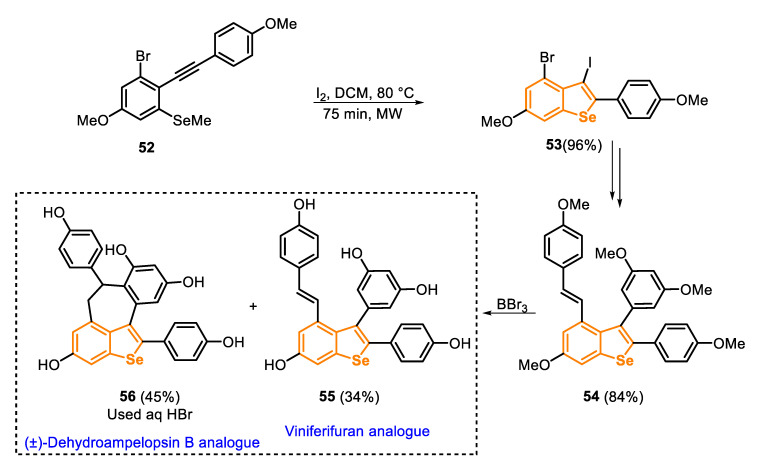
Use of type C precursor in the synthesis of viniferifuran and (±)-dehydroampelopsin B *Se*-analogues.

Since then, these protocols have paved the way for the development of several transformations in organic synthesis. For instance, this was the key step in the recently-described synthesis of benzo[*b*]selenophene-based analogues of the resveratrol dimers viniferifuran and (±)-dehydroampelopsin B (Scheme 39) [147]. The 5-*endo*-*dig* cyclization was carried out under microwave irradiation, in just 75 min, using I_2_ as an electrophile and DCM as a solvent, affording the 3-iodo-selenophene **53** in 96% yield. This benzo[*b*]selenophene was the precursor of the intermediate **54**, obtained after two consecutive Suzuki–Miyaura coupling reactions. Finally, the demethylation of compound **54** was performed with BBr_3_ (6 equiv) in DCM, giving the *Se*-analogue of viniferifuran **55** (34% yield) and the demethylation/cyclization product (±)-dehydroampelopsin B *Se*-analogue (14% yield), after 3 days of reaction. The authors reported that the cyclization product **56** was favored (45% yield) by adding BBr_3_ (15 equiv) and aqueous HBr.

**Scheme 40:**
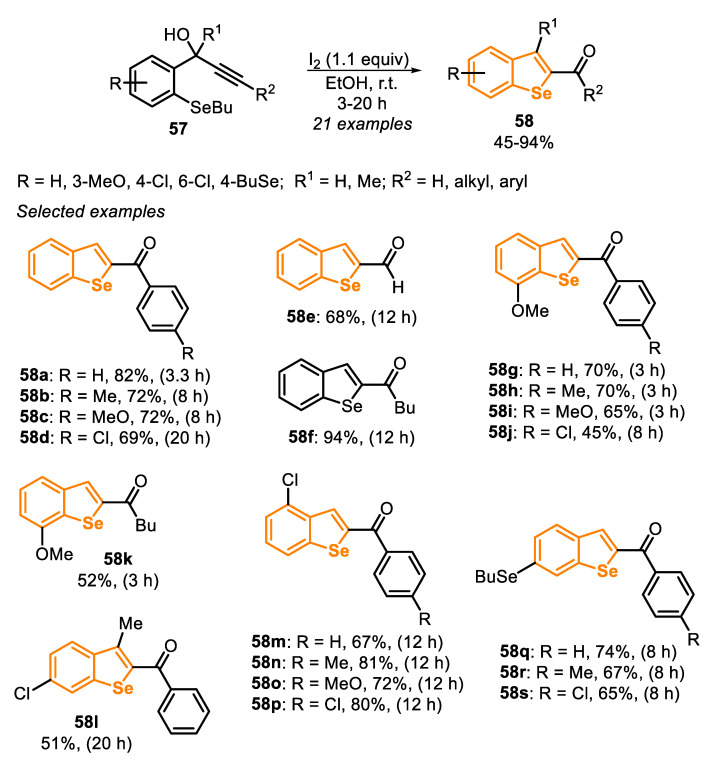
Scope of the synthesis of 2-acylbenzo[*b*]selenophenes **58**.

A close-related protocol was described in 2015 [148], through the intramolecular cyclization of [2-(butylselanyl)phenyl]propynols **57** (related to type C precursor), using I_2_ (1.1 equiv) at room temperature and under aerobic conditions (Scheme 40). Under these conditions, a study on the reaction scope was carried out, using several electron-rich and electron-deficient substrates, allowing for the synthesis of twenty-one 2-acylbenzo[*b*]selenophenes **58** analogues in moderate to good yields (45–94%). The protocol was general and efficient for a variety of [2-(butylselanyl)phenyl]propynols **57** containing aryl or alkyl groups directly bonded to the triple bond. A good result was also obtained, starting from terminal alkyne, giving the product **58e** at 68% yield after 12 h. Different electron-donating and electron-withdrawing substituents directly connected to the central aromatic ring (fused to selenophene) were explored in the reaction, and no significant electronic effect was observed.

**Scheme 41:**
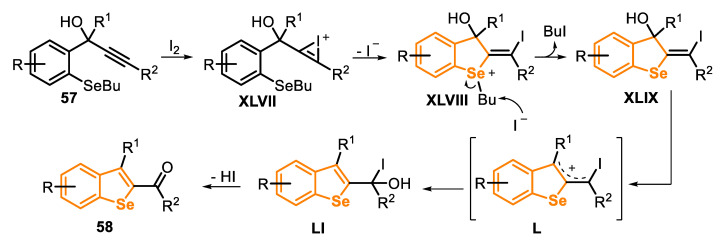
Proposed mechanism for the synthesis of 2-acylbenzo[*b*]selenophenes **58**.

The reaction mechanism involves initially the formation of the iodonium ion **XLVII**, which is formed by the addition of I_2_ to the C-C triple bond (Scheme 41). The *anti*-nucleophilic attack of the selenium atom at the activated iodonium intermediate produces the salt **XLVIII**, via a 5-*exo-dig* cyclization. The removal of the alkyl group via S_N_2 displacement by the iodide anion, present in the reaction mixture, generates the vinylic iodide **XLIX** and butyl iodide. Then, a 1,3-migration of hydroxy, via the formation of the allylic cation **L**, gives the alcohol **LI**, which affords the 2-acylbenzo[*b*]selenophenes **58** after the elimination of HI (Scheme 41).

**Scheme 42:**
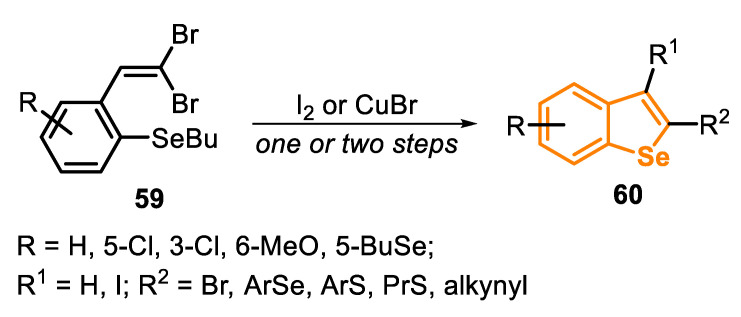
1,1-Dibromoalkenes as precursors of benzo[*b*]selenophenes.

In recent years, the use of *ortho*-butylselanyl-substituted 1,1-dibromoalkenes **59** as precursors to access benzo[*b*]selenophenes **60** has shown great advances, through the application of two different strategies: the use of I_2_ and of TM-based catalysis (Scheme 42).

**Scheme 43:**
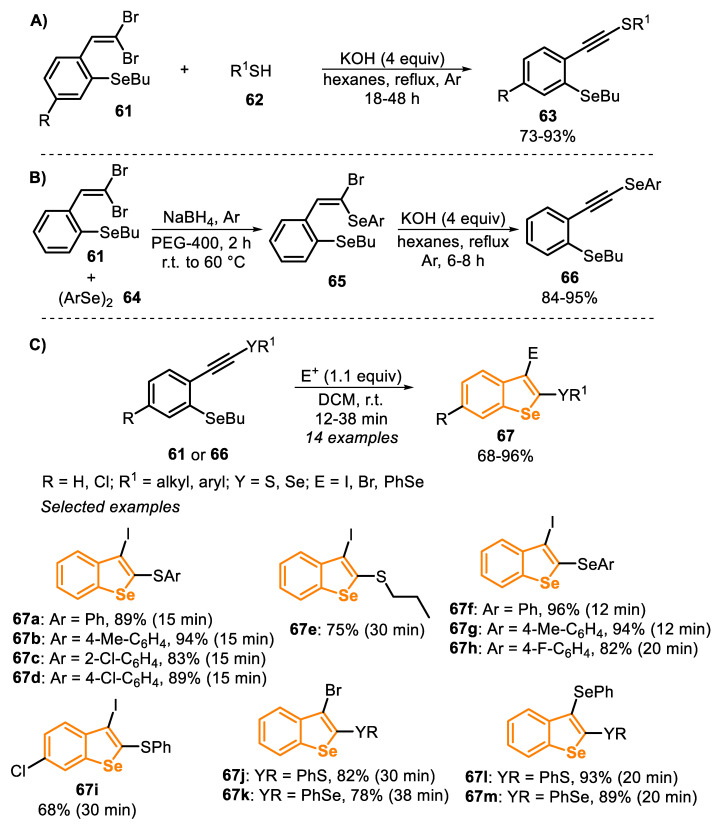
Protocols for obtaining type C precursors and benzo[*b*]selenophenes **67**.

In 2017, some of us [149] described a protocol that is close-related to the one previously addressed using I_2_ to promote the cyclization of type C precursors, which are obtained in situ from *ortho*-butylselanyl-substituted 1,1-dibromoalkenes **61**. The versatility of the compound **61** was explored in the synthesis of the intermediate thioalkynes **63**, by the reaction with aryl or alkyl thiols (1.2 equiv) in the presence of KOH (4 equiv.) and hexanes (degassed) as a solvent, under reflux temperature in argon atmosphere, by 18 to 48 h (Scheme 43A). In order to obtain the selenoalkynes **66**, a two-step protocol was performed: initially, the *o*-butylselanyl-substituted 1,1-dibromoalkene **61** was submitted to the reaction with diaryl diselenides **64**, in the presence of NaBH_4_ and PEG-400 as the solvent. After 2 h, at 60 °C under argon atmosphere, the respective (*E*)-1-bromo-1-arylselenoalkenes **65** were obtained. In the second reaction, the crude product was submitted to a dehydrobromination using KOH (1 equiv) in refluxing hexanes for 6 to 8 h (Scheme 43B).

The type C precursors **61** and **66** were reacted in the presence of I_2_ (1.1 equiv) and using DCM as a solvent to prepare a broad scope of benzo[*b*]selenophenes **67** (Scheme 43C). The reactions were carried out in short reaction times (12–38 min), and the desired products **67a**-**i** were obtained in good to excellent yields (68–96%). Other electrophilic species, such as Br_2_ and PhSeBr, were successfully used in substitution to iodine (compounds **67j**-**m**).

**Scheme 44:**
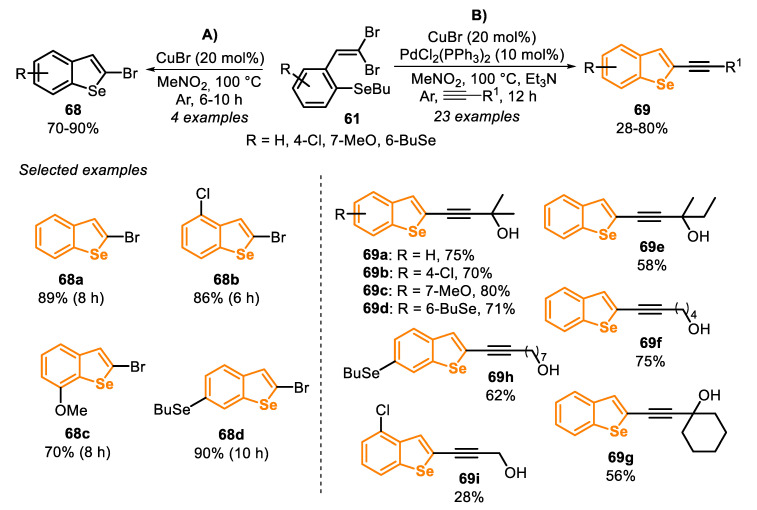
Synthesis of 2-substituted benzo[*b*]selenophenes.

In the same year, the synthesis of benzo[*b*]selenophenes derivatives **68** and **69**, through the Cu(I)-catalyzed annulation of *ortho*-butylselanyl-substituted 1,1-dibromoalkenes **61** was described [150]. The reaction was carried out in the presence of CuBr (20 mol%) in MeNO_2_ as a solvent at 100 °C, giving four unprecedented 2-bromobenzo[*b*]selenophenes **68** in 70% to 90% yields (Scheme 44A). Due to the efficiency of this protocol, the authors extended their studies to the synthesis of 2-alkynylbenzo[*b*]selenophenes **69**, starting from *o*-substituted 1,1-dibromoalkenes **61**, through a sequential cyclization/Sonogashira cross-coupling strategy (Scheme 44B). In this one-pot procedure, the 2-bromoselenophenes **68** were generated in situ, as above, and then reacted with propargyl alcohols in the presence of PdCl_2_(PPh_3_)_2_ (10 mol%) and Et_3_N. A total of twenty-three 2-alkynylbenzo[*b*]selenophenes **69e-g** were prepared in 28–80% yields after 12 h of reaction. The presence of bulky groups on the alkyne caused a slight decrease in the reaction yield, affording the products **69** moderate yields. The method was not sensitive to the presence of electron-donor and electron-withdrawing groups in the pendant phenyl of the 1,1-dibromoalkenes **61**, and the respective products **69** were obtained in moderate to good yields, except the compound **69i**, derived from 2-propyn-1-ol, which was obtained in only 28% yield. This protocol did not work with other alkynes, (different from propargyl alcohols), such as phenylacetylene and 1-pentyne, affording, in these cases, the products **69** together an inseparable mixture of homocoupling byproducts.

**Scheme 45:**
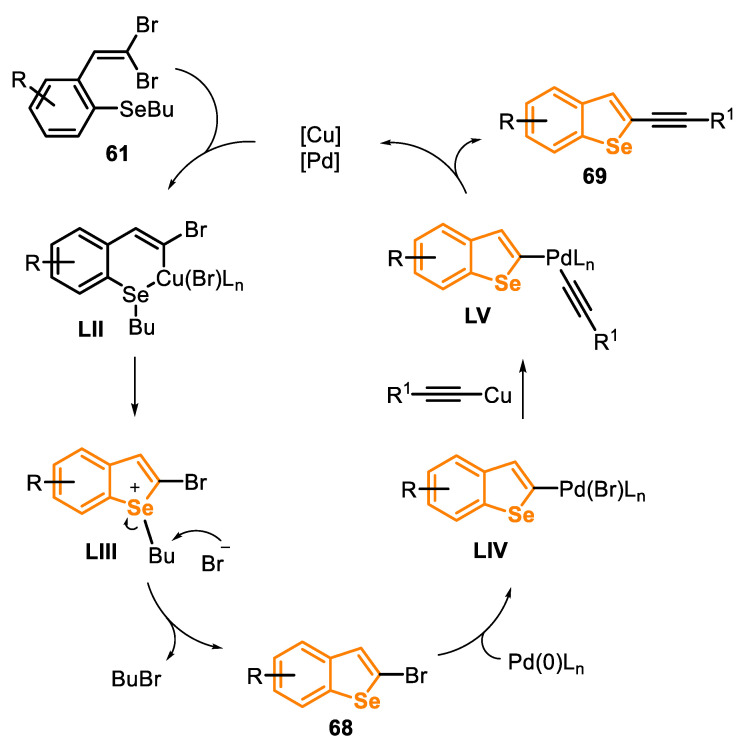
Proposed mechanism of the synthesis of 2-substituted benzo[*b*]selenophenes **68** and **69**.

The proposed mechanism of the reaction starts with the oxidative addition of copper to the *ortho*-substituted 1,1-dibromoalkene **61** to give the 2-bromobenzo[*b*]selenophene **68** via an intramolecular Ullmann reaction process, followed by nucleophilic substitution on the selenium atom. The oxidative addition of 2-bromobenzo[*b*]selenophene **68** to the Pd(0) species affords the Pd(II) intermediate **LIV**, which reacts with the copper-activated alkyne to give the intermediate **LV**. Then, the species **LV** undergoes a reductive elimination, generating the product **69** and releasing the catalysts for a new cycle (Scheme 45).

**Scheme 46:**
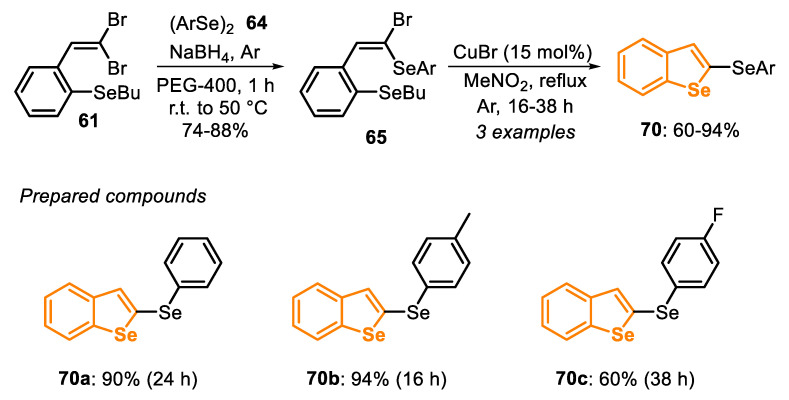
Synthesis of 2-arylselanyl-benzo[*b*]selenophenes **70**.

In 2019, Cu(I)-catalyzed annulation of *ortho*-SeBu-substituted (*E*)-1-bromo-1-arylselenoalkenes **65** (obtained as described in Scheme 43B) was described in [151]. The best condition for this annulation was obtained using CuBr (15 mol%) as the catalyst in MeNO_2_ as the solvent under argon atmosphere at reflux temperature for 16–38 h (Scheme 46). Using this condition, three 2-arylselanyl-benzo[*b*]selenophenes **70** were obtained in 60% to 94% yields. The presence of electron-withdrawing groups linked to the aromatic ring of the diselenide **64** (R = 4-F) negatively affected the reaction efficiency, giving the product **70c** in 60% yield after 38 h, while the electron-rich *p*-tolyl derivative (R = 4-Me) was more reactive, affording the selenophene **70b** in 94% yield after 16 h (Scheme 46).

**Scheme 47:**
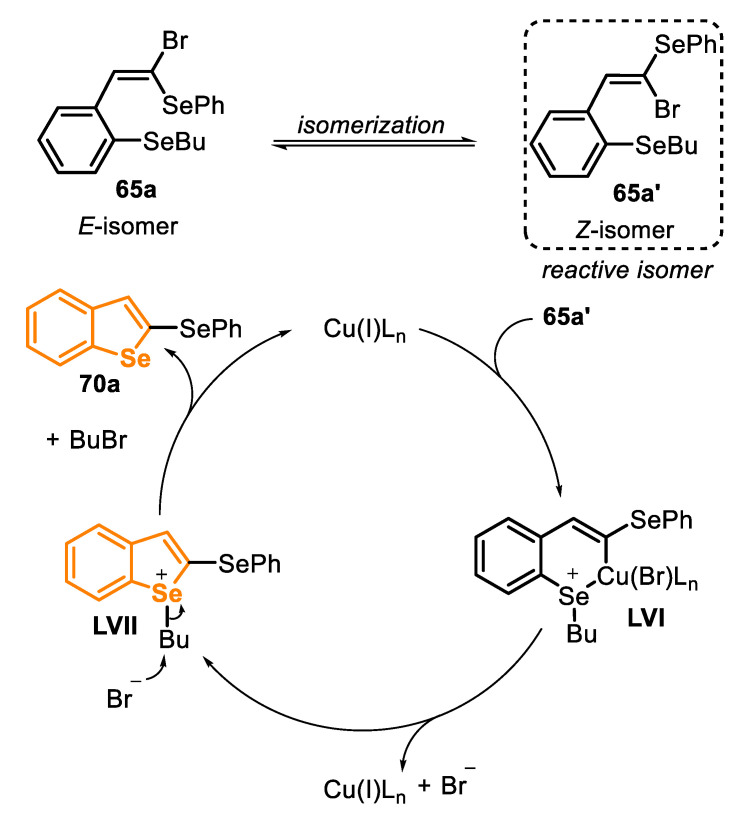
Mechanism of the synthesis of 2-arylselanyl-benzo[*b*]selenophenes **70**.

The key reaction step in intramolecular cyclization is the conversion of the *E*-isomer **65a** to the *Z*-isomer **65a’**, which has a more suitable configuration to undergo an oxidative addition to the coordination sphere of the Cu(I) species. This hypothesis was confirmed by carrying out a control experiment using pure (*E*)-1-bromo-1-(phenylselanyl)-2-phenylethene under the standard reaction conditions. In this case, after 24 h, 45% of the *E*-isomer was isomerized to the *Z*-one (determined by GC/MS analysis). After the coordination of the Cu(I) species, which gives the intermediate **LVI**, an Ullmann-type coupling generates the intermediate **LVII**, releasing to the reaction medium, the copper catalyst and bromide. Finally, the bromide acts as a nucleophile, attacking the butyl group through an S_N_2 mechanism, giving the product **70a** and eliminating 1-bromobutane as a byproduct (Scheme 47).

Another strategy to access benzo[*b*]selenophenes was described in 2019 [152]. In this protocol, the Oxone^®^ was used as an alternative oxidant for the generation of *Se*-based electrophile in situ, through an oxidative cleavage of the Se-Se bond of diorganyl diselenides.

**Scheme 48:**
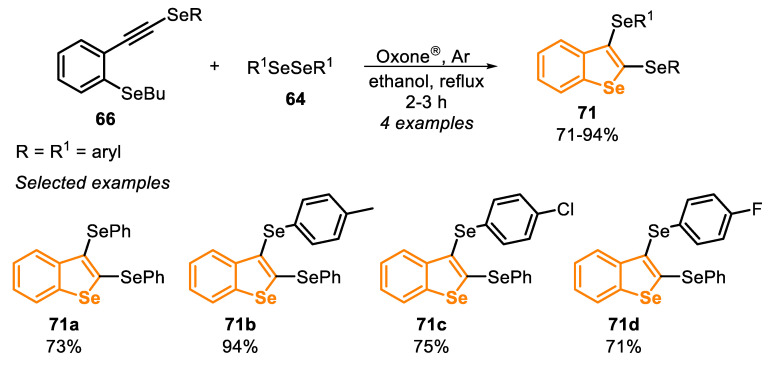
Scope of the synthesis of 2,3-bis-organoselanyl-benzo[*b*]selenophenes **71** using Oxone^®^.

The electrophilic cyclization of *ortho*-SeBu selenoalkynes **66** (type C precursors) was promoted by the system Oxone^®^/diorganyl diselenide **64**, affording the respective 2,3-bis-selanyl-benzo[*b*]selenonophenes **71** in good to excellent yields (71–94%) with short reaction times (2–3 h) (Scheme 48). The scope was extended to different *ortho*-functionalized chalcogenoalkynes, accessing eleven benzo[*b*]thiophenes and seven benzo[*b*]furans derivatives.

**Scheme 49:**
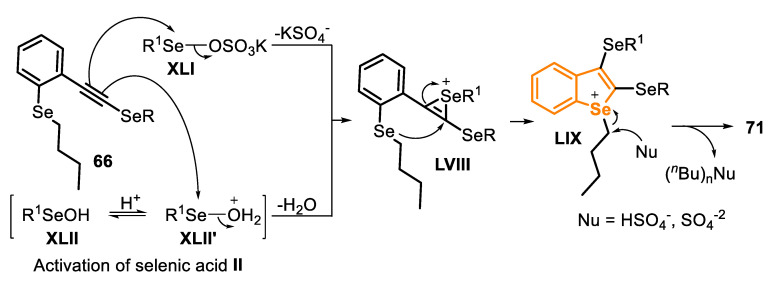
The reaction mechanism of the synthesis of **71**.

The mechanism, which involves the formation of the Se-based electrophilic species from the system Oxone^®^/diorganyl diselenide, was already exposed in the previous session (Scheme 36). In the cyclization step, the C(*sp*) of the type C precursor reacts with the active species **XLI** and **XLII’**, generated in situ, to give the intermediate seleniranium **LVIII** (activation step). Then, the intermediate **LVIII** undergoes an intramolecular *anti*-attack by the *Se* atom (a 5-*endo-dig* cyclization), producing the intermediate **LIX**. Finally, the butyl group attached to the selenium atom is removed via an S_N_2 reaction, by a nucleophile, to generate the product **71** (Scheme 49).

**Scheme 50:**
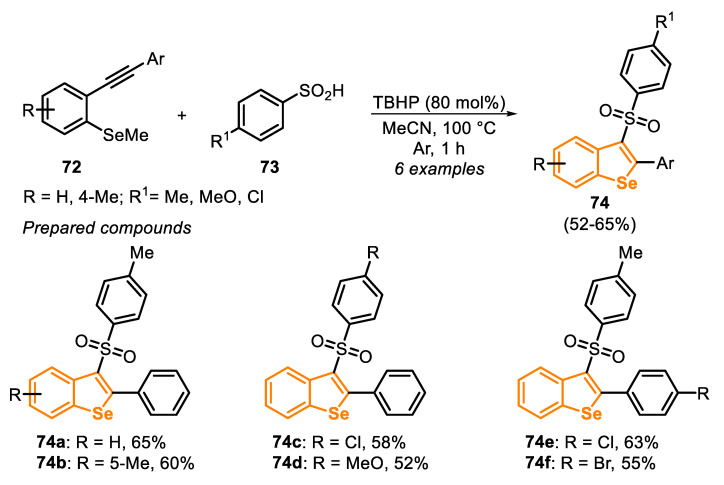
Scope of the formation of 3-(arylsulfonyl)benzoselenophenes **74** using sulfinic acid.

Recently, two important protocols using type C precursors in radical reactions to prepare benzoselenophenes were developed [153,154]. The first method was described in 2017, through a TBHP-promoted radical cyclization between *ortho*-alkynylaryl selenides **72** (a type C precursor) and sulfinic acids **73** (Scheme 50) [153]. The protocol required a high load of TBHP (80 mol%) and MeCN as a solvent, being the resulting mixture stirred at 100 °C for 1 h. By this procedure, six different 3-(arylsulfonyl)benzoselenophenes **74** were prepared in moderate yields (52–65%). The method was shown to be general and was not sensible to the presence of electron-withdrawing and electron-donor groups attached to the aromatic rings of both the *ortho*-alkynyl arene and the sulfinic acid portions (Scheme 50).

**Scheme 51:**
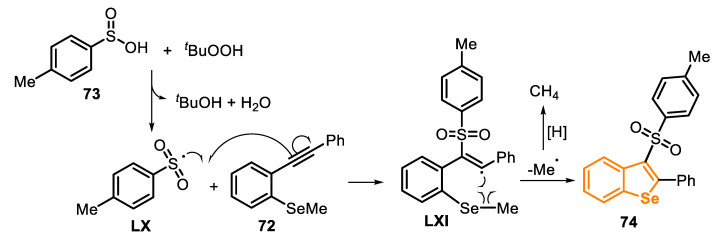
Proposed Mechanism of the formation of **74** using TBHP.

The mechanism of the synthesis of 3-(arylsulfonyl)benzoselenophene derivatives **74** involves an initial combination of TBHP and sulfinic acid **73**, giving the sulfinyl radical **LX**, through a single electron transfer (SET) pathway, which selectively attacks the C(*sp*) bond of **72** to afford the vinyl radical **LXI**. Subsequently, the vinyl radical **LXI** reacts with the SeMe moiety, in a 5-*exo*-*trig* cyclization mode, leading to the product **74** and the elimination of methyl radical. Furthermore, the methyl radical is trapped by a hydrogen in the reaction medium (Scheme 51).

**Scheme 52:**
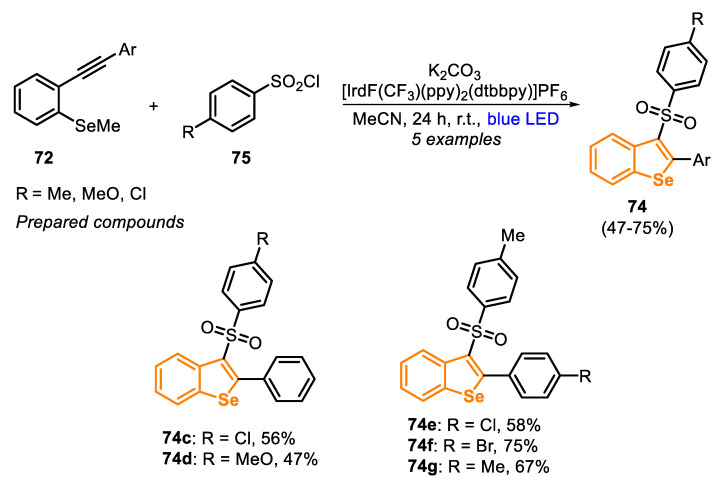
Scope of the synthesis of benzoselenophenes **74** using sulfuryl chloride.

In 2018, the same group [154] described an alternative procedure to prepare the 3-(arylsulfonyl)benzoselenophene derivatives **74**, through the visible light photoredox-catalyzed cascade annulation of *ortho*-alkynylaryl selenides **72** (type C precursor) with sulfonyl chlorides **75** (Scheme 52). The reaction was performed in the presence of an Ir-based photocatalyst (2 mol%) and K_2_CO_3_ (2 equiv), in MeCN at room temperature and under blue LED (5 W) irradiation, affording five different functionalized selenophenes **74** in 47–75% yield.

**Scheme 53:**
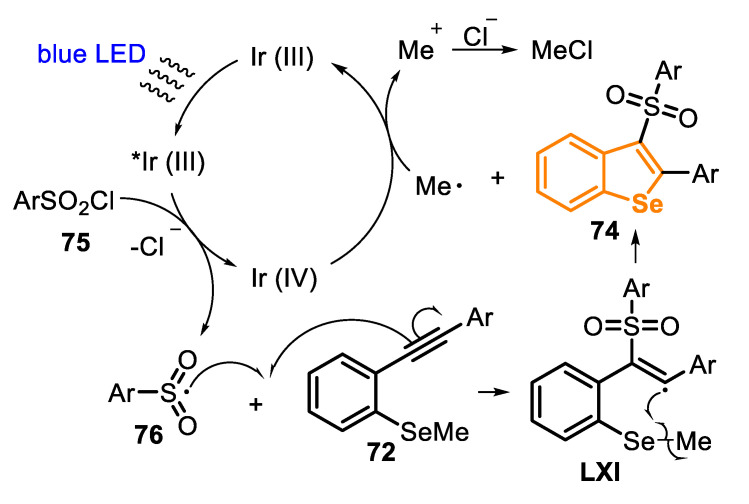
The reaction mechanism of the photocatalyzed synthesis of **74**.

The reaction mechanism is very similar to the annulation previously described in Scheme 51, except in the radical formation step. According to the authors, under the visible light irradiation, the Ir(III)-photocatalyst is converted to the excited state *Ir(III), through a metal-to-ligand charge-transfer (MLCT) phenomena. In the sequence, the excited species *Ir(III) undergoes an oxidative quenching in the presence of sulfonyl chloride **75**, through an SET process, releasing the sulfonyl radical **76** into the reaction medium and affording the oxidized Ir(IV) species. Then, an addition of the sulfonyl radical **76** to the alkynyl moiety on the molecule **72** takes place to give the vinyl radical **LXI**, which performs a radical intramolecular attack in the SeMe moiety (a 5-*exo*-*trig* cyclization), affording the product **74** and releasing a methyl radical species. Finally, the methyl radical (Me**^•^**) reduces the Ir(IV), regenerating the Ir(III) photocatalyst and releasing a methyl cation (Me^+^), which is converted to MeCl (Scheme 53).

**Scheme 54:**
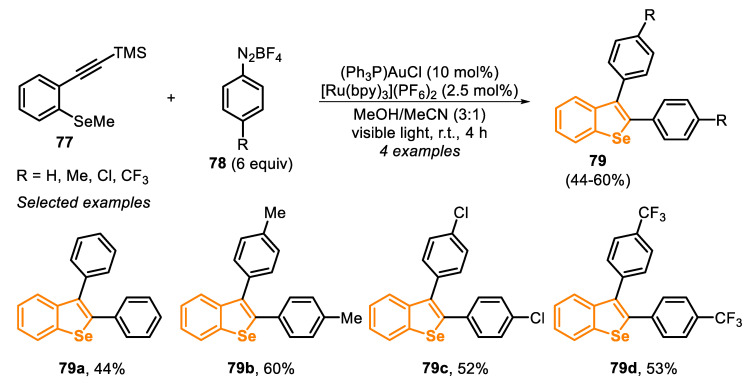
Gold-photoredox catalysis in the synthesis of 2,3-diarylbenzo[*b*]selenophenes.

Another visible light-mediated electrophilic cyclization to prepare benzoselenophenes was developed in 2017 [155]. In this protocol, the authors employed the type C precursors, generated in situ by a gold-catalyzed *sila*-Sonogashira-type coupling, which can be further trapped by an *Se*-based nucleophile, with a concomitant second arylation, through a domino reaction sequence. In this protocol, a mixture of TMS-(ethynyl) arene **77**, arenediazonium salt **78**, an Au-based catalyst and a Ru-based photocatalyst in MeOH/MeCN (3:1) as the solvent, was irradiated by visible light (21 W fluorescent light bulb) for 4 h, yielding the respective products **79** in moderate yields (Scheme 54).

**Scheme 55:**
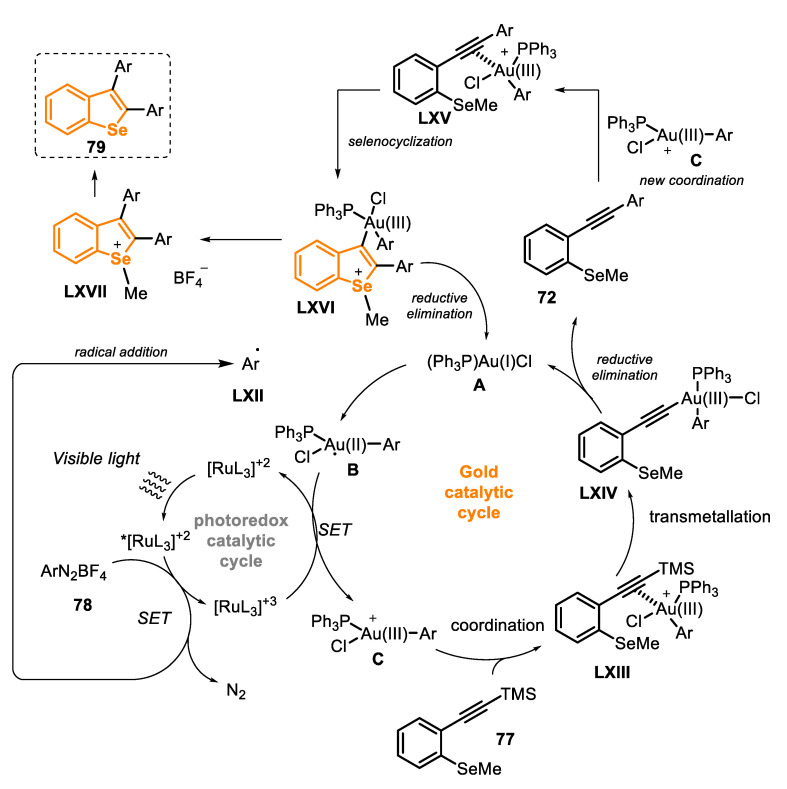
Gold-photoredox catalysis mechanism in the synthesis of selenophene **79**.

The reaction mechanism involves two simultaneous catalytic cycles, the Ru(II)-photoredox and the Au(I)-catalysis (Scheme 55). Initially, the Ru(II) complex is irradiated by the visible light, affording the excited long-lived Ru(II)* species, which, in the presence of the diazonium salt **78**, undergoes an oxidative quenching, being converted to the Ru(III) species, releasing into the reaction medium N_2_ and one equivalent of the aryl radical **LXII** (the photoredox catalytic cycle). The aryl radical **LXII** reacts with the Au(I) precatalyst **A** to be converted to the unstable radical organogold(II) intermediate **B**. The Au-centered radical **B** is able to reduce Ru(III) to the Ru(II) photocatalyst, through an SET process, being converted to the cationic organogold(III) derivative **C**, a strong electrophile. Then, the intermediate **C**, in the presence of TMS-(ethynyl) arene **77**, is converted to the intermediate **LXIII**, through the alkyne coordination with the Au complex, which undergoes a transmetallation of the C(*sp*)-Si bond, giving the Au(III) intermediate **LXIV**. Finally, **LXIV** undergoes a reductive elimination, releasing the precursor **77** and regenerating the Au(I) catalyst **A** to restart a new reaction cycle (the gold catalytic cycle). Then, the compound **72** generated in situ, coordinates with the highly electrophilic Au(III) species **C**, affording the intermediate **LXV**, which undergoes an intramolecular seleno-cyclization, through a 5-*endo*-*dig* reaction, to access the cyclic intermediate **LXVI**. Finally, a reductive elimination regenerates the Au(I) catalyst and delivers the cationic intermediate **LXVII**, which is converted to the desired product **79**.

**Scheme 56:**
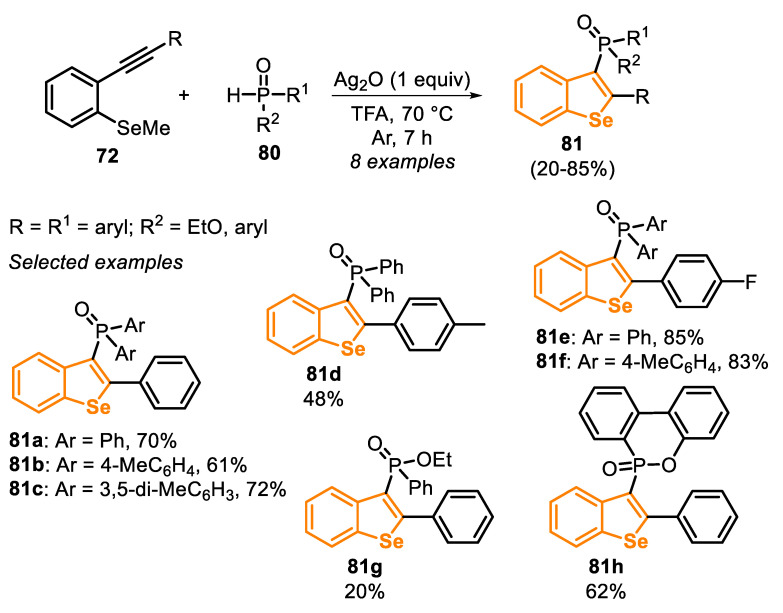
Scope of the synthesis of 3-phosphinoylbenzo[*b*]selenophenes **81**.

In 2019, a new protocol for the construction of benzo[*b*]selenophenes through an Ag-promoted radical cyclization between the type C precursor **72** and secondary phosphine oxides **80** was developed (Scheme 56) [156]. This protocol allowed us to extend the synthesis to a wide scope of benzoselenophene derivatives **81a**-**c** using phosphines bearing aryl groups (Ph, 4-MeC_6_H_4_, 3,5-di-MeC_6_H_3_) in good yields (61–72%). The influence of electron-donor (R = 4-MeC_6_H_4_) and electron-withdrawing substituents (R = 4-FC_6_H_4_) in the pendant aromatic ring of the arylalkynes (type C precursors) was evaluated. The electron-rich system was less reactive than the electron-deficient and the neutral ones, and the product **81d** was obtained in 48% yield, while **81e** and **81f** were isolated in 85% and 83% yields, respectively. Moreover, the reactivity of differently substituted phosphine oxides **80** was accessed, with ethyl phenylphosphinate and 9,10-dihydro-9-oxa-10-phosphaphenanthrene 10-oxide (DOPO) derivatives affording the respective benzoselenophenes **81g** and **81h** in 20% and 62% yields.

**Scheme 57:**
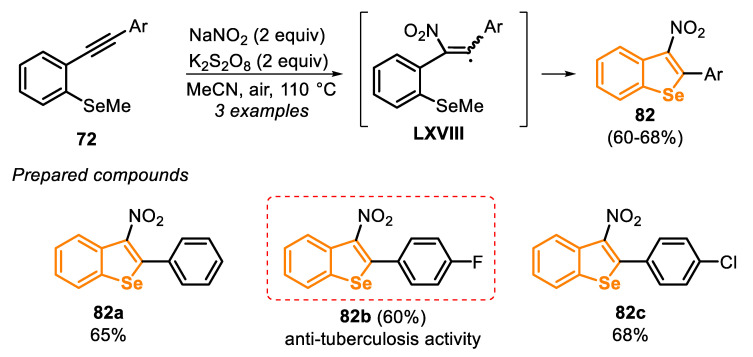
Scope of the synthesis of 3-nitrobenzo[*b*]selenophenes **82**.

In this year (2020), a similar radical protocol was described via a 5-*exo-trig* cyclization of type C precursors **72**, being the reaction promoted by K_2_S_2_O_8_ (2 equiv) and NaNO_2_ (2 equiv) as oxidants, in the presence of MeCN as a solvent [157]. The method was mainly used to prepare benzo[*b*]thiophenes (19 examples, 46–90% yields) and was extended to prepare selenium analogues (only three examples). After stirring the reaction mixture for 12 h at 110 °C under open air, 3-nitrobenzo[*b*]selenophenes **82a-c** were obtained in moderate yields (60–68%). The protocol was applied to neutral (Ar = phenyl) and electron-deficient (Ar = 4-FC_6_H_4_ and 4-ClC_6_H_4_) aryl groups, and no electronic effect was observed (Scheme 57).

This was the first example of using highly unstable 2-nitrovinyl radicals in the formation of C-S and C-Se bonds, circumventing the problems usually faced in classical methods, including the application of harsh reaction conditions, by employing strong acids, low yields and poor regioselectivity. However, the reaction of the thio-analogue type C precursors with the TMS-ethynyl and alkylethynyl moieties did not afford the desired products, evidencing a limitation of the method. The reaction mechanism was proposed after functional density theory (DFT) calculations and control experiments, confirming the presence of the vinyl radical intermediate **LXVIII**. In addition, the products were tested for their anti-tuberculosis activity in vitro, and the fluoro-containing 3-nitrobenzoselenophene **82b** showed a potent anti-tuberculosis activity against drug-resistant *Mtb* strains, with an MIC_90_ of 2 μg mL^−1^.

**Scheme 58:**
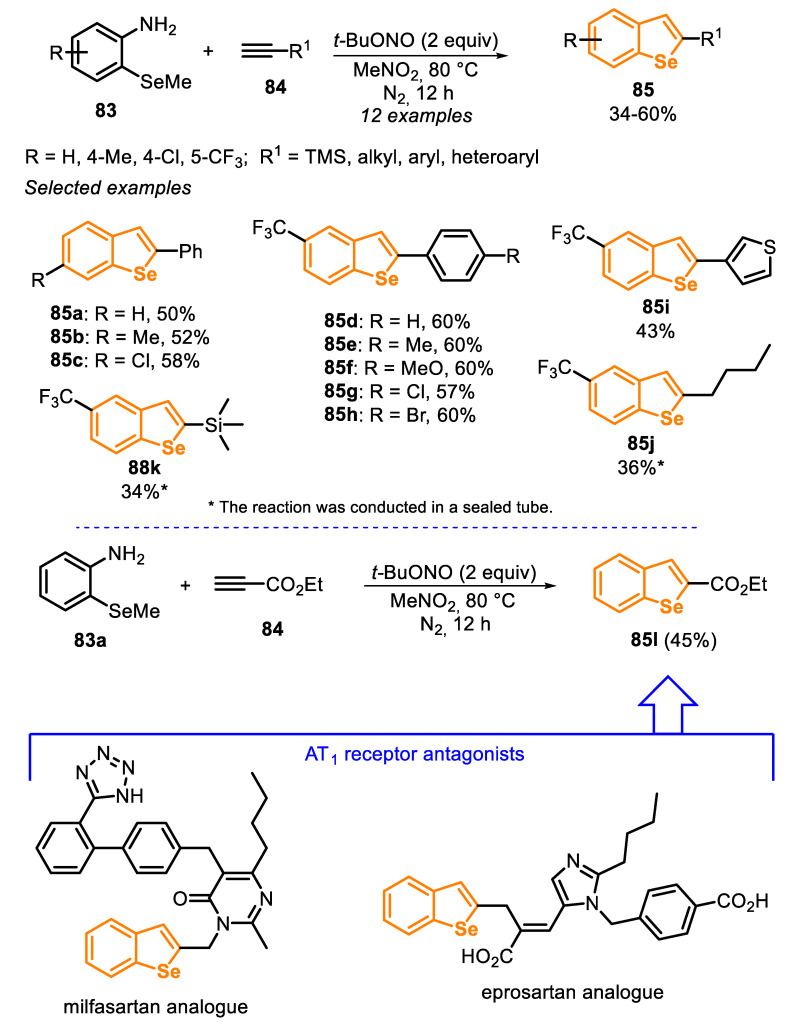
Synthesis of 2-substituted benzo[*b*]selenophenes and AT_1_ receptors.

Closing our discussion on the reactivity of type C precursors in the synthesis of benzoselenophenes, a new strategy to access benzo[*b*]selenophenes **85** from *ortho*-methylselanyl-arylamines **83** and alkynes **84** is presented (Scheme 58) [158]. The protocol presented the advantage of generating aryl radical intermediates from arylamines **83** and *tert*-butyl nitrite (*t-*BuONO), avoiding the use of the difficult to prepare and unstable diazonium salts. *t-*BuONO acts in the diazotization reaction of *ortho*-methylselanyl-arylamines **83**, affording the reactive radical species in situ. The optimal procedure involves the reaction between *ortho*-methylselanyl-arylamines **83** and alkyne **84** (3 equiv) in the presence of *t-*BuONO (2 equiv) as a nitrosating agent and MeNO_2_ as the solvent, at 80 °C for 12 h, under nitrogen atmosphere. The methodology was regioselective and the products **85** were obtained in low to moderate yields (34–60%). Apparently, the method was not sensible to the electronic effects of substituents in the *para*-position of the aryl substituted alkynes **84** nor in the arylamines **83**, as shown for the yields obtained for the benzo[*b*]selenophenes **85d**–**h** vs. **85b**-**c**. In addition, the authors used different terminal alkynes **84f**–**h**, including 3-ethynylthiophene, hexyne, ethynyltrimethylsilane, obtaining the respective products **85i**–**k** in satisfactory yields. The scope of the reaction was extended to several thio-analogues to prepare benzothiophenes in good yields. Ethyl propiolate **84i,** together with *ortho*-methylselanyl-benzylamine **83a,** were used in the preparation of benzoselenophene **85l**, a key intermediate in the synthesis of milfasartan and eprosartan analogues.

**Scheme 59:**
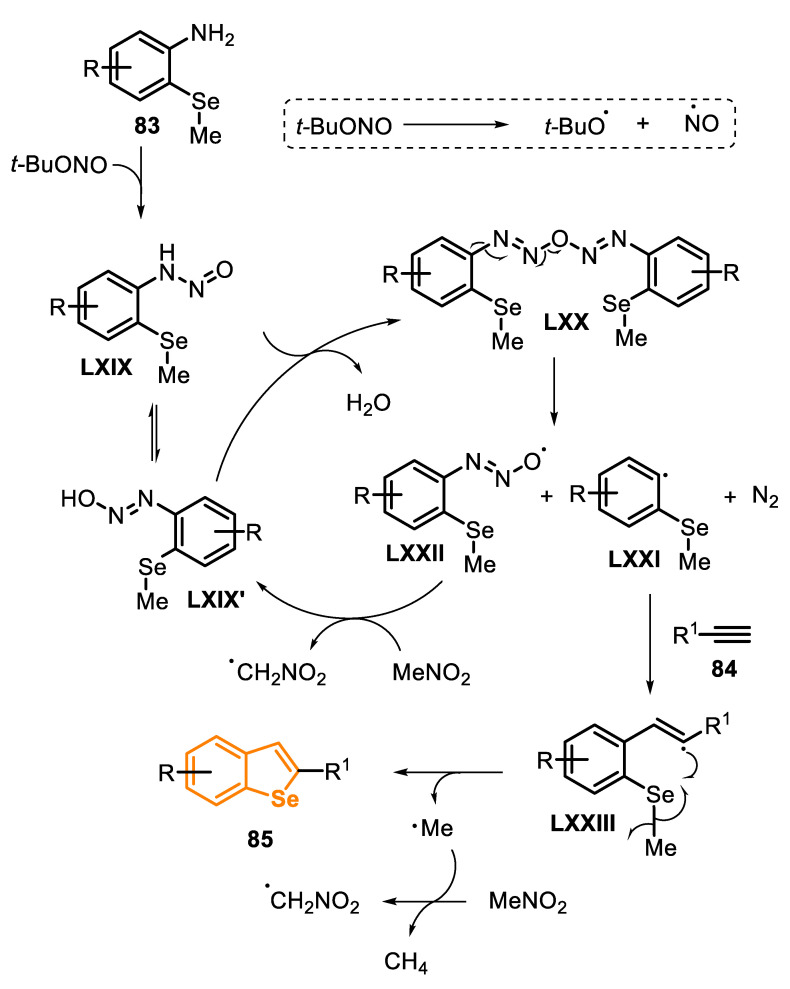
Proposed mechanism of the radical cascade reaction to prepare **85**.

After some control experiments, a mechanism for the intermolecular radical cascade reaction was proposed (Scheme 59). Initially, arylamine **83** reacts with *t*-BuONO to generate nitrosamine **LXIX**, which undergoes a self-condensation reaction to generate the diazo anhydride **LXX**. The N-O homolysis of **LXX** provides the aryl radical **LXXI**, along with the azoxy radical **LXXII**, and releases molecular nitrogen. Then, the radical **LXXII** abstracts a hydrogen atom from the solvent, in order to generate the diazo hydroxide **LXIX’** (easily interconverted into nitrosamine **LXIX**), which can react again to give the aryl radical **LXXI**. The addition of **LXXI** to alkyne **84** leads to the vinyl radical **LXXIII**, which reacts via an intramolecular homolytic substitution, by the Se atom, to give the benzo[*b*]selenophene **85**, followed by the elimination of methyl radical (Me**^•^**), which can react via an *H*-abstraction, from the solvent, giving CH_4_.

### 3.2. Starting from Type D Precursors

In the last few decades, *ortho*-halo-substituted ethynylarenes **86** (type D precursors) have been frequently used as starting material in the synthesis of benzoselenophenes **87**. The most common protocol involving these precursors involves the generation of Se-based nucleophilic species in situ, using sodium borohydride (NaBH_4_) and protic solvents. Some parameters, including stoichiometry, solvents and the presence of base, may directly affect the identity of the formed reactive species [159].

**Scheme 60:**
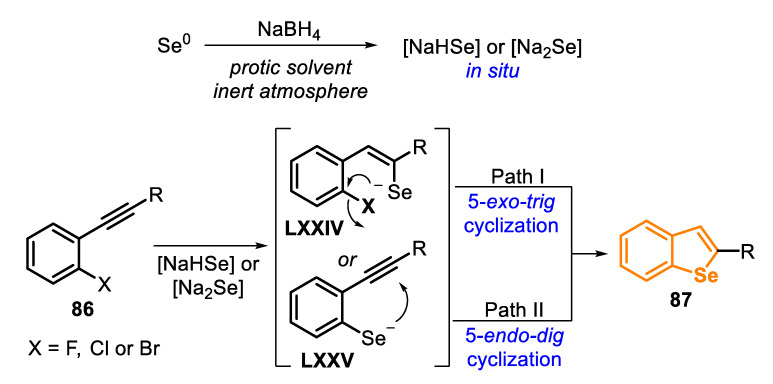
General protocol for cyclization of type D precursors using NaBH_4_.

In general, the mechanism for the synthesis of benzoselenophenes **87** using nucleophilic Se species, and type D precursors **86** can follow two different paths (Scheme 60). One possible pathway involves firstly the formation of the intermediate **LXXIV**, after an attack by NaHSe or Na_2_Se (pre-formed in situ) to the triple bond of **86**, which is followed by an intramolecular S_N_Ar reaction (Path I). However, the process can also follow a nucleophilic substitution (S_N_Ar) of the selenide anion, by the X group, generating the intermediate **LXXV**, which undergoes an intramolecular seleno-cyclization by the reaction of the selenium anion to the triple bond (Path II).

**Scheme 61:**
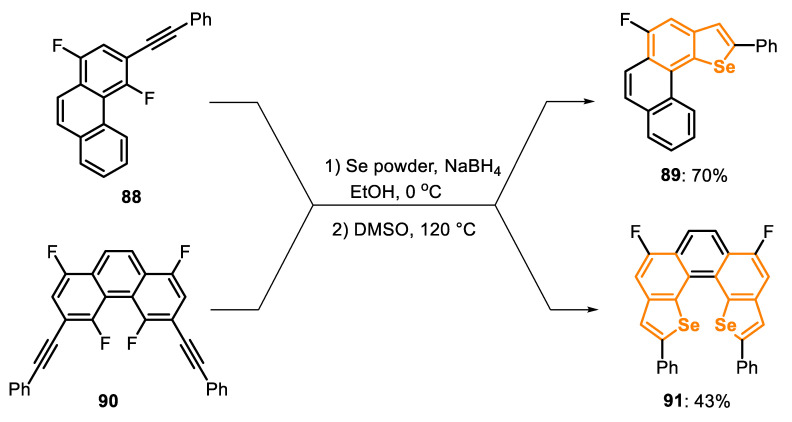
Synthesis of mono- and diselena[5]helicenes **89** and **91**.

These reaction pathways have been widely applied over the last few years and can be used in several organic transformations, even for the construction of complex structures. For example, in this year the synthesis of *Se*-helicenes **89** and **91** derivatives was reported [160]. In this one-pot protocol, the Se-based nucleophilic species were firstly generated (in situ) through the reaction of Se powder with NaBH_4_ at 0 °C for 40 min. In the sequence, the respective *ortho*-fluoro-ethynylarene **88** and **90** and DMSO were added to the reaction flask, and the temperature was increased to 120 °C. After 18 h, the respective products **89** and **91** were obtained in 70% and 43% yields, respectively (Scheme 61).

**Scheme 62:**
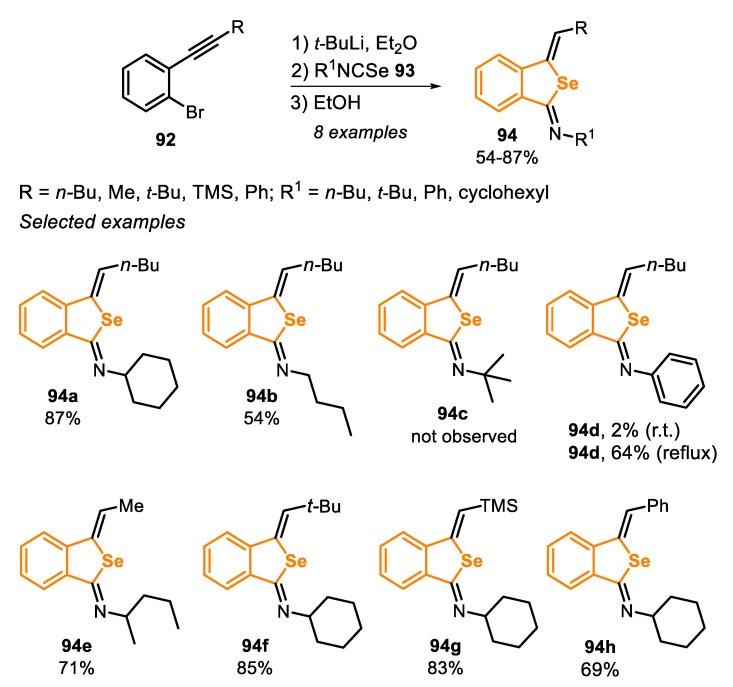
Synthesis of 3-methylidenebenzo[*c*]selenophenes **94**.

Type D precursors normally lead to the formation of benzo[*b*]selenophenes; however, benzo[*c*]selenophenes can also be obtained by the lithiation of *ortho*-bromo-ethynylarene **92** with *t-*BuLi in Et_2_O, followed by the addition of isoselenocyanates **93** and EtOH as a proton source (Scheme 62) [161]. Through this method, it was possible to prepare eight different 3-methylidenebenzo[*c*]selenophenes **94** in moderate to good yields (54–87%). In this work, the authors used different *o*-bromo-ethynylarenes **92** with alkyl, phenyl and trimethylsilyl groups linked to the triple bond, that were reacted with alkyl and phenyl isoselenocyanates **93**. Phenyl isoselenocyanate (R^1^ = C_6_H_5_) was less reactive than the alkyl analogues, and reflux temperature was necessary to afford the respective product **94d** acceptable yields (64% vs. 2% yield at room temperature). The method was not applicable to the bulky *tert*-butyl isoselenocyanate **93c**; in this case, a complex mixture was obtained instead of the expected benzo[*c*]selenophenes **94c** (Scheme 62).

**Scheme 63:**
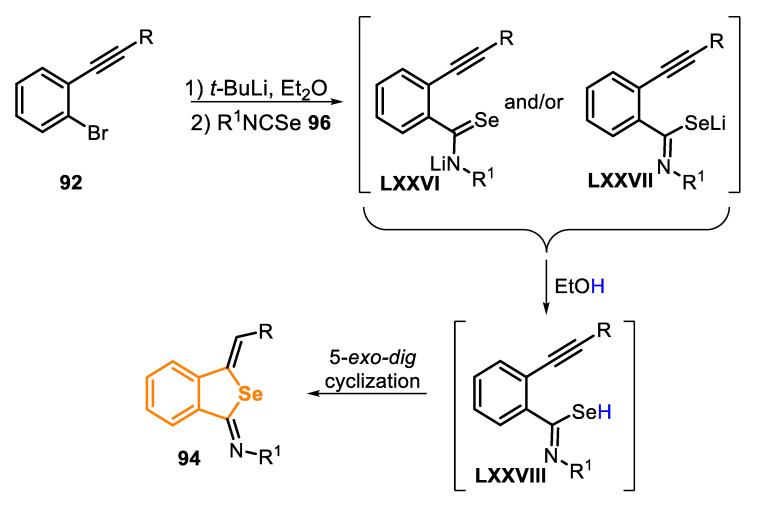
Proposed mechanism of the synthesis of benzo[*c*]selenophenes **94**.

Aiming to elucidate the reaction mechanism, a reaction was carried out in the absence of a proton source (EtOH), but no product was formed, and the starting isoselenocyanate **93** was recovered. Based on this, it was proposed that the reaction passes by the formation of selenol intermediate **LXXVIII** through the reaction of EtOH with intermediates **LXXVI** and **LXXVII**. Once formed, the key selenol **LXXVIII** reacts by a selective 5-*exo-dig* intramolecular seleno-cyclization, giving the respective benzo[*c*]selenophene **94** (Scheme 63).

**Scheme 64:**
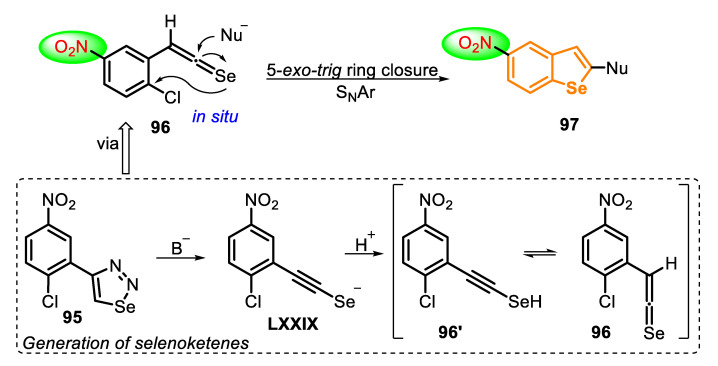
General protocol to obtaining selenoketenes **96** from **95**.

In 2013, a new approach, based on an intramolecular S_N_Ar cyclization to obtain benzo[*b*]selenophenes **97,** was developed [162]. The protocol is grounded in a ring closure of *ortho*-chloro-arylselenoketenes **96**, by a 5-*exo-trig* cyclization, using different secondary amines both as nucleophile and solvent (Scheme 64). The selenoketene precursor **96** is generated in situ from the decomposition of 4-substituted 1,2,3-selenadiazoles **95** (with elimination of N_2_). The treatment of **95** with a strong base afforded the alkynylselenolate **LXXIX** (confirmed by trapping using MeI), that is converted to a mixture of alkyneselenol **96′** and tautomeric selenoketene **96**, after protonation.

**Scheme 65:**
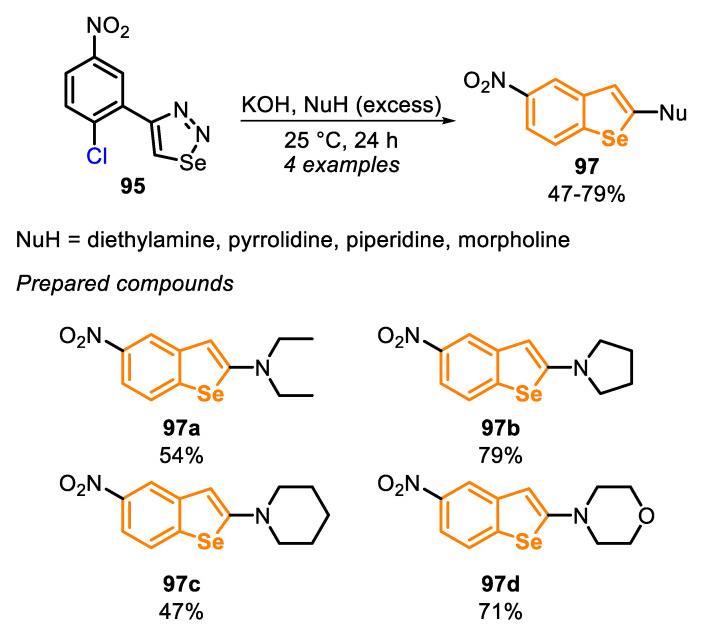
Synthesis of benzo[*b*]selenophenes **97**.

Using this strategy, four nitro-substituted benzo[*b*]selenophenes derivatives **97** were prepared in moderate to good yields (47–79%). Initially, 4-(2-chloro-5-nitrophenyl)-1,2,3-selenadiazole (**95**) were treated with KOH for the formation of the selenoketene **96** in situ. In the sequence, a secondary amine was added, and the resulting mixture was stirred at 25 °C for 24 h (Scheme 65).

The presence of the NO_2_ group at the *para* position to the chlorine is mandatory for the reaction success, once it favors the intramolecular S_N_Ar reaction, due to the strong electron-withdrawing effect. No cyclized product was observed starting from 4-(2-chlorophenyl)-1,2,3-selenadiazole, even at 130 °C, just accessing selenoamide derivative after the final acidic treatment. The protocol was expanded to the synthesis of benzo[*b*]thiophene derivatives, starting from 4-(2-chloro-5-nitrophenyl)-1,2,3-thiadiazoles instead the 1,2,3-selenadiazole analogues.

**Scheme 66:**
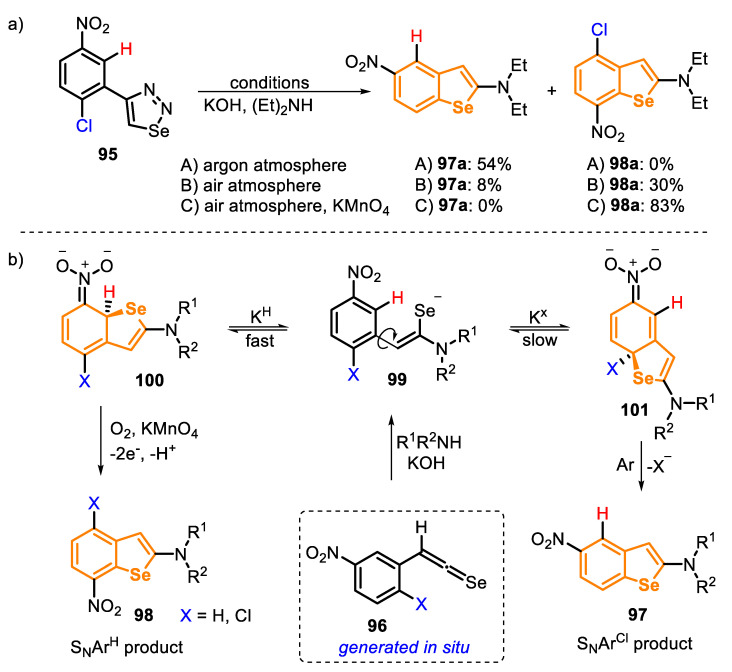
Control in the formation of **97** and **98** (**a**) and possible pathways of the cyclization of selenoketene **96** (**b**).

In 2013, an interesting study on the 5-*exo-trig* cyclization of selenoketenes **96**, obtained in situ from the treatment of 4-(2-chloro-5-nitrophenyl)-1,2,3-selenadiazole (**95**), was reported, as described above [163]. The authors discovered that the reaction course can be completely changed by adding a strong oxidant in the reaction medium. When the reaction described in Scheme 65 was performed under an air atmosphere, a mixture was created between the S_N_Ar^Cl^ **97a** and S_N_Ar^H^ **98a** products, which were isolated in 8% and 30% yields, respectively (Scheme 66, condition B). This observation indicates that the formation of the *σ*^H^-adduct is faster than the *σ*^Cl^ one. The reaction was directed to the exclusive formation of the *σ*^H^-adduct, through the addition of a strong oxidant (KMnO_4_), by an oxidative nucleophilic substitution of hydrogen, giving the product **98a** in 83% yield (Scheme 66, condition C).

**Scheme 67:**
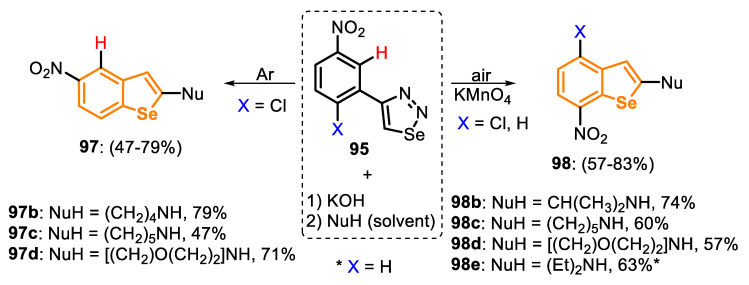
Regioselective synthesis of benzo[*b*]selenophenes **97** and **98**.

The high regioselectivity achieved in the reaction allowed us to access four benzoselenophene derivatives via S_N_Ar^Cl^ (**97**: 47–79% yields) and five via S_N_Ar^H^ (**98**: 57–83% yields), using different secondary amines as nucleophiles. One example was conducted starting from 4-(5-nitrophenyl)-1,2,3-selenadiazole (X = H), which, after the generation of the ketene intermediate, reacted with EtNH_2_ in the presence of KMnO_4_/air, affording the respective selenophene **98e** in 63% yield (Scheme 67).

**Scheme 68:**
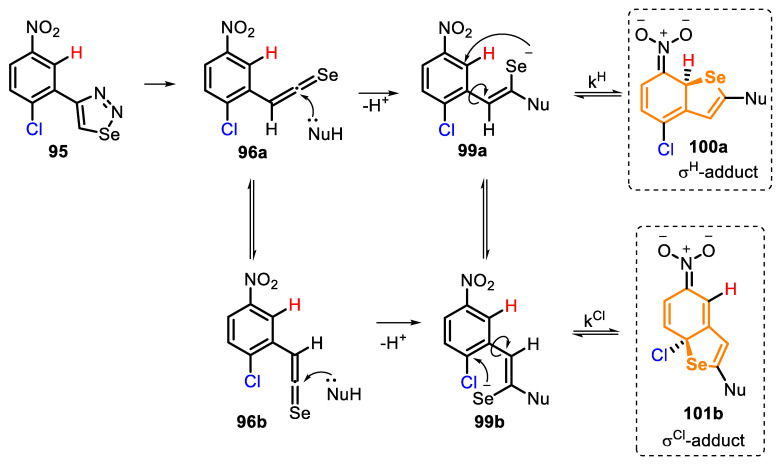
Formation of *σ*^H^- and *σ*^Cl^-adducts from selenoketene **96**.

According to the authors, the selenoketene intermediate **96** can exist in two rotational conformers, **96a** and **96b**. In each case, the sequential nucleophilic attack occurs on the opposite side of the bulky aryl ring, affording the conformeric eneselenolates **99a** and **99b**, which, after the intramolecular attack from selenium anion, lead to the respective *σ*^H^- and *σ*^Cl^-adducts **100a** and **101b** (Scheme 68).

### 3.3. Starting from Type E Precursor

A third usual strategy to prepare benzoselenophenes is through the cyclization of type E precursors. This approach involves electrophilic aromatic substitution reactions (S_E_Ar), employing different selenium derivatives and arylalkynes (the type E precursors).

**Scheme 69:**
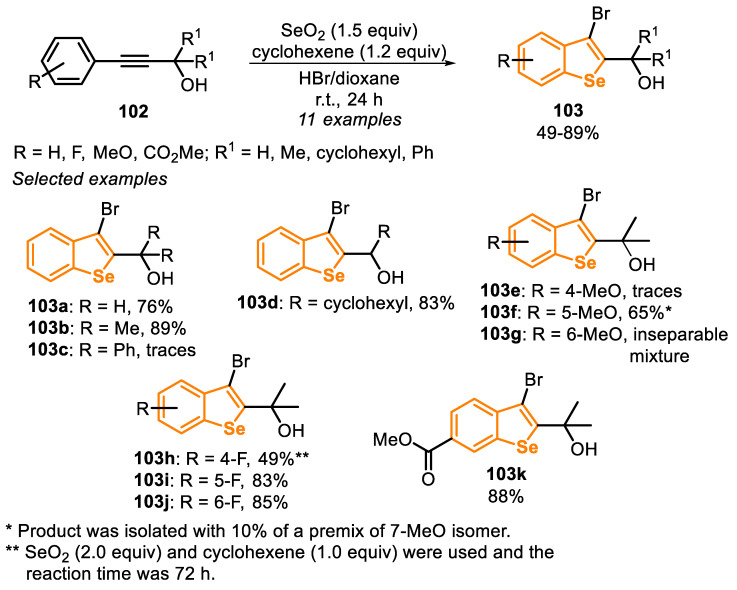
Synthesis of 3-bromo-benzo[*b*]selenophenes **103**.

In 2014, an important protocol based on the selenobromination of type E precursors **102** was reported [164]. The best condition for this transformation involves stirring a mixture of SeO_2_ (1.5 equiv) in dioxane as a solvent in the presence of 48% HBr (to generate selenium(IV) bromide in situ) with cyclohexene (1.2 equiv) as an additive, at room temperature for 24 h (Scheme 69). Under these conditions, a study on the reaction scope was carried out using several electron-rich and electron-deficient substrates, allowing for the synthesis of eleven 3-bromo-benzo[*b*]selenophenes analogues **103** in moderate to good yields (49–89%). The reaction was efficient using different substituted phenylpropargyl alcohols **102**; however, when phenyl groups were bonded to the propargylic carbon (R^1^ = C_6_H_5_), only traces of the respective product **103c** were observed, which can be explained by steric hindrance imparted by the phenyl groups.

In general, S_E_Ar reactions are strongly dependent on the nature of the substituents in the aromatic ring, being favored using directing groups (DG). This dependence is evident when the methoxyl group (MeO) was present in the aromatic ring of arylpropargyl alcohol **102**. In this case, only traces of product **103e** (R = 2-MeO) were observed (the dibromo derivative was the major product), while an inseparable mixture was obtained in the attempt to reach the product **103g** (R = 4-MeO) and the corresponding dibromo derivative. However, the product **103f** (R = 3-MeO) was obtained in 65% yield, but in an isomeric mixture (10% of 7-methoxy isomer). In general, the presence of strong electron-withdrawing group favored the transformation. For instance, products **103i** (R = 3-F), **103j** (R = 4-F) and **103k** (R = 4-CO_2_Me) were obtained in 83%, 85% and 88% yields, respectively. The presence of a fluorine in the *ortho*-position of the phenyl ring in **102** (R = 2-F), caused a decrease in the reactivity, and the product **103h** was obtained in 49% yield, after 72 h of reaction (Scheme 69).

**Scheme 70:**
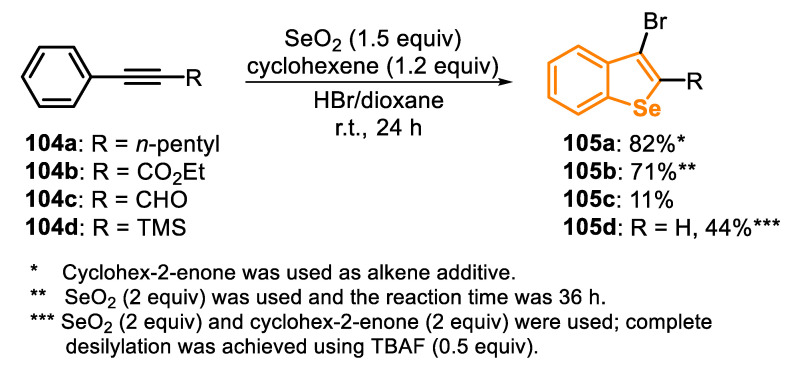
Synthesis of 3-substituted-benzo[*b*]selenophenes **105**.

The reaction with SeO_2_ was extended to other internal aryl alkynes **104**: hept-1-ynylbenzene (**104a**), ethyl phenylpropiolate (**104b**), 3-phenylpropiolaldehyde (**104c**) and trimethyl(phenylethynyl)silane (**104d**). Good results were obtained using **104a** and **104b**, and the respective benzoselenophenes **105a** and **108b** were obtained in 82% and 71% yields after small modifications of the optimal conditions used with the propargyl alcohols **102**. The aldehyde **104c**, however, afforded the respective product **105c** in only 11% yield, while the TMS-protected alkyne **104d** generated the deprotected benzoselenophene **105d**, in 44% yield, which was desilylated in situ by the addition of TBAF (0.5 equi) in the reaction medium (Scheme 70).

**Scheme 71:**
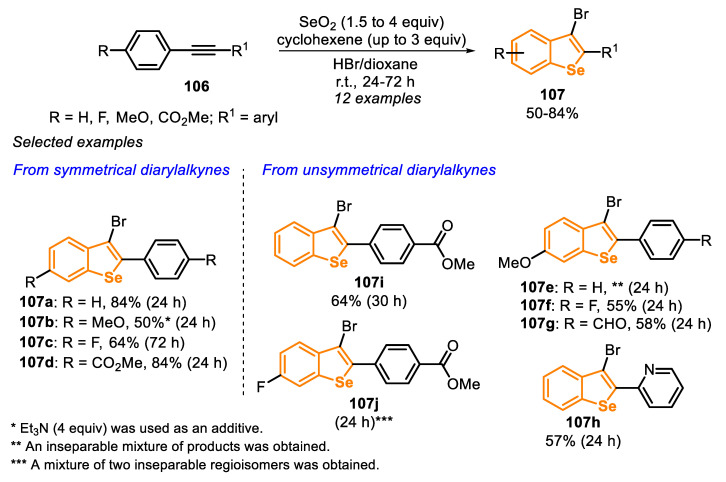
Synthesis of 2-aryl-3-bromo-benzo[*b*]selenophenes **107**.

In the following year (2015), additional outcomes on the SeO_2_-mediated cyclization of type E precursors were described (Scheme 71) [165]. The authors used different symmetrical and unsymmetrical diarylalkynes **106**, demonstrating the strong influence of different substituents attached to the aromatic ring. This effect was more substantial when unsymmetrical substituted diarylalkynes **106** were used. For example, starting from alkyne **106e** (R = 4-MeO; R^1^ = C_6_H_5_), the bromination of the triple bond was observed, including a low regioselectivity, resulting in a complex mixture of inseparable products, without the presence of a measurable amount of the expected benzoselenophene **107e** (Scheme 71). A different behavior was observed when electron-withdrawing groups were attached in the para-position of the pendant phenyl ring, such as in **106f** (R^1^ = 4-FC_6_H_4_) and **106g** (R^1^ = 4-CHOC_6_H_4_), and the respective benzoselenophenes **107f** and **107g** were obtained in 55% and 58% yields. The electronic effect, however, is not predictable, once the substrate **106j**, bearing two electron-withdrawing groups in distinct aromatic rings (R = 4-F; R^1^ = 4-CO_2_MeC_6_H_4_), was not a suitable substrate for the reaction. In this case, the bromination of the C-C triple bond was not observed; however, the transformation showed a poor regioselectivity, leading to the formation of both possible regioisomers and an inseparable mixture of compounds (Scheme 71, compound **107j**).

**Scheme 72:**
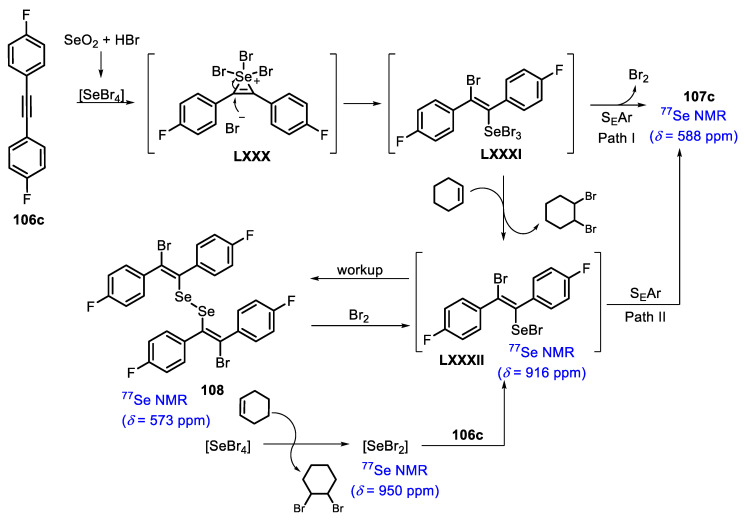
Reaction pathways to prepare benzoselenophenes **107**.

To collect insights into the mechanism of the reaction, the authors chose the synthesis of the difluoro-substituted derivative **107c**, once it is a slow reaction (72 h) and allows the different steps to be monitored by ^19^F and ^77^Se NMR spectroscopy. Initially, SeBr_4_ adds to the C-C triple bond of **106c**, giving the cationic seleniranium intermediate **LXXX**. This selenobromination step was crucial to achieve the regioselectivity in the unsymmetrical substrates, in which the nucleophilic attack of the bromide anion occurs at the carbon with the lowest electron density. Thus, a more polarized C-C triple bond leads to more pronounced regioselectivity. Based on this, the reaction can follow two different pathways. Firstly, the authors suggest that the cyclization step passes by the formation of the intermediate **LXXXI**, due to the fast formation of the cyclized product **107c**, in the absence of an alkene additive. This could be explained by the fact that more electrophilic Se(IV) **LXXXI** species are involved in the S_E_Ar step, in comparison to the intermediate Se(II) **LXXXII**. The bromine equivalent that was released during the cyclization process reacts with the starting material **106c** by the bromination of the C-C triple bond, giving the byproducts (Scheme 72, Path I). In the second possibility, the reaction proceeds via the intermediate **LXXXII** (detected by ^77^Se NMR), obtained by the reaction between the intermediate **LXXXI** and cyclohexene, which is directly converted into the product **107c** via a S_E_Ar (Scheme 72, Path II). Additionally, besides being formed from the intermediate **LXXXI**, the intermediate **LXXXII** can also be obtained by the direct addition of SeBr_4_ to the alkene additive, before the addition of substrate **106c**, affording SeBr_2_ (identified by ^77^Se NMR). When the crude reaction mixture was quenched with brine and AcOEt, the diselenide derivative **108** was formed (isolated in 42% yield), after a disproportionation of the intermediate **LXXXII** and subsequent Se-Se bond formation. This observation provided substantial evidence of a stereospecific *anti*-1,2-addition in the selenobromination step. In another experiment, the diselenide **108** was able to be converted to the intermediate **LXXXII** by the oxidative addition of Br_2_ (1 equiv), also following the path II (Scheme 72).

**Scheme 73:**
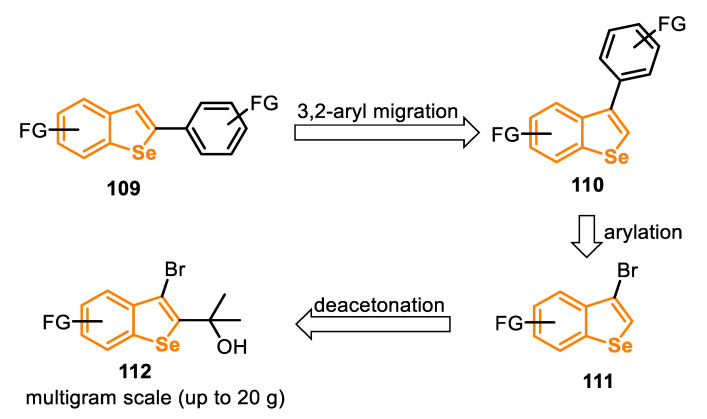
Retrosynthetic strategy for the preparation of 2-aryl **109** and 3-aryl-benzo[*b*]selenophenes **110**.

In addition to this protocol, in 2016, a general method to prepare functionalized 2- and 3-arylbenzo[*b*]selenophenes (**109** and **110**, respectively), through a 3,2-diaryl migration starting from the same precursor **111** was developed (Scheme 73) [31]. This approach overcame the regioselectivity limitations faced previously, which restricted the scope of the direct cyclization of unsymmetrical diarylalkynes. An advantage of this 1,2-aryl shift in the rearrangement step is the elimination of the debromination/bromination steps. A large and complex study was carried out to evaluate the redox properties, free-radical-scavenging ability, and cytotoxicity of the prepared compounds against cancerous cell lines. Based on the experimental results, the structure–activity relationships (SARs) of these compounds were evaluated.

### 3.4. Starting from Type F Precursors

**Scheme 74:**
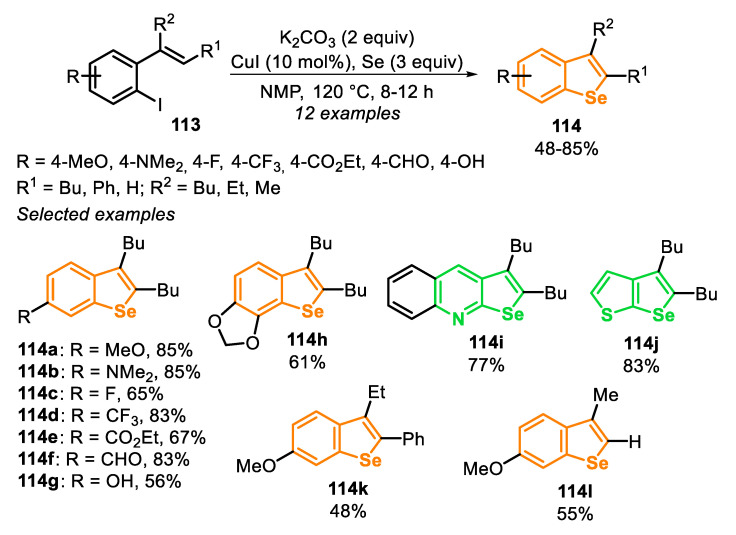
Scope of the synthesis of benzo[*b*]selenophenes through copper-catalyzed C-Se coupling/cyclization.

In 2013, the synthesis of benzoselenophenes **114** through a Cu-catalyzed reaction of *ortho*-alkenylaryl iodide **113** (a type F precursor) with selenium powder and K_2_CO_3_, in *N*-methyl-2-pyrrolidone (NMP) as the solvent at 120 °C was described (Scheme 74) [166]. Under these reaction conditions, twelve different benzoselenophenes **114a**-**g**, bearing electron-donating and electron-withdrawing substituents at the C6-position, were prepared in moderate to good yields. When the 6,7-methylenedioxy derivative **113h** was employed, the corresponding benzoselenophene **114h** was obtained in 61% yield. This protocol was also suitable to prepare the seleno[2,3-*b*]quinoline **114i** and the selenopheno[2,3-*b*]thiophene **114j** in good yields. Finally, benzoselenophenes **114k** and **114l**, bearing different substituents on the C2- and C3-positions, were obtained in the poorest yields.

**Scheme 75:**
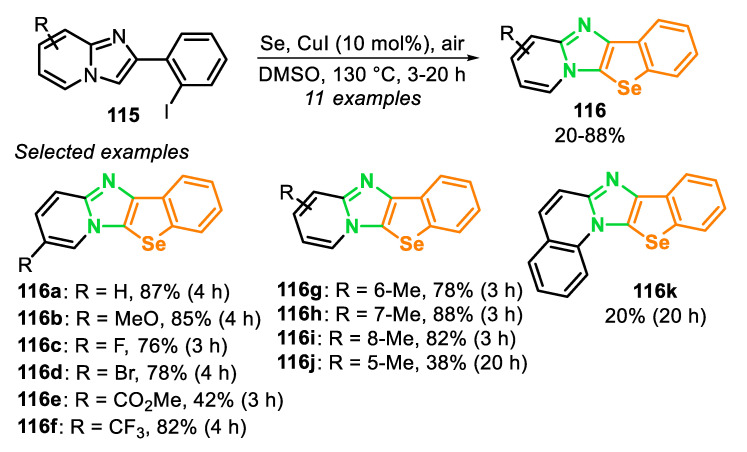
Scope of the synthesis of benzo[*b*]selenophene-fused imidazo[1,2-*a*]pyridines **116**.

Similarly, in recent years, several protocols have been described for the synthesis of fused benzoselenophene derivatives through TM-catalyzed tandem cyclization reactions, employing *ortho*-haloarene heterocycles and elemental selenium. In this context, in 2016, the Cu-catalyzed tandem cyclization of 2-(2-iodophenyl)imidazo[1,2-*a*]pyridines **115** with selenium, for the synthesis of benzo[*b*]selenophene-fused imidazo[1,2-*a*]pyridines **116** via Ullmann-type *Se*-arylation and C(*sp^2^*)-H selenylation was described (Scheme 75) [167]. The reaction was performed using CuI (10 mol%) as the catalyst, under aerobic conditions in DMSO at 130 °C. The efficiency and versatility of this protocol were demonstrated by employing different 2-(2-iodophenyl)imidazo[1,2-*a*]pyridines **115** as substrate. Substrates **115** bearing neutral (R = H), electron-donating (R = Me and MeO) and electron-withdrawing (R = F, Br, CO_2_Me and CF_3_) groups at the C6 position of the imidazopyridine ring were suitable substrates, affording the corresponding benzoselenophene-fused imidazopyridines **116a**-**g** poor to good yields (42–87%). The procedure was also applicable to the synthesis of 6-bromo-2-(2-iodophenyl)imidazopyridine **115d** (78% yield), and the highly reactive bromine moiety remained intact. Additionally, 2-(2-iodophenyl)imidazopyridines **115** with methyl groups at the C7- and C8-positions afforded the corresponding coupling products **116h** and **116i** in 88% and 82% yields, respectively. Reactions starting from C5-substituted 2-(2-iodophenyl)imidazo[1,2-*a*]pyridines **115j** and **115k** afforded the products **116j** and **116k** in low yields after 20 h of reaction, demonstrating the high sensitivity of the reaction to steric effects.

**Scheme 76:**
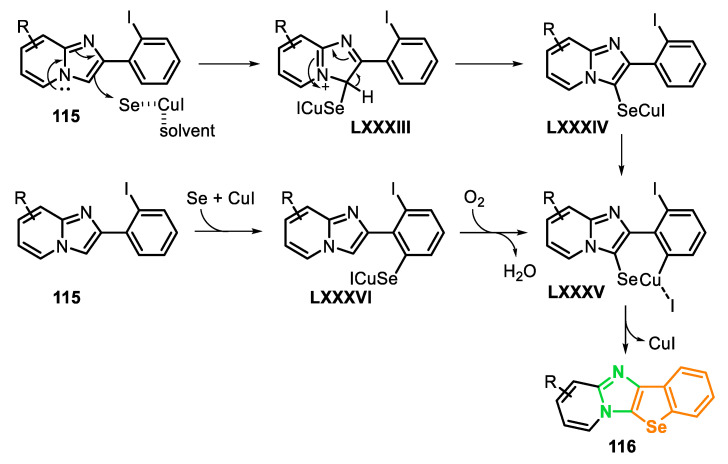
Mechanism of the synthesis of benzo[*b*]selenophene-fused imidazo[1,2-*a*]pyridines **116**.

The proposed mechanism for the tandem *Se*-arylation between 2-(2-iodophenyl)imidazopyridine **115** and Se powder involves, in the first step, the formation of the intermediate **LXXXIV** via the intermediate **LXXXIII**, by a Cu-mediated electrophilic substitution of the iodo-imidazopyridine **115** with Se (Scheme 76). After, the intermolecular oxidative addition of Cu(II) to the Ph-I bond, **LXXXIV** forms the metallacycle **LXXXV**. Finally, a reductive elimination of the intermediate **LXXXV** leads to benzoselenophene-fused imidazopyridine **116** and regenerates the Cu catalyst. Another possible route involves the Cu-catalyzed reaction of **115** with Se powder to form the complex **LXXXVI**, via an oxidative addition of **115** in the CuI coordinating sphere. Then, an oxidative cyclization of **LXXXVI** occurs, affording the metallacycle **LXXXV**, which is converted to the desired product **116** through a reductive elimination.

**Scheme 77:**
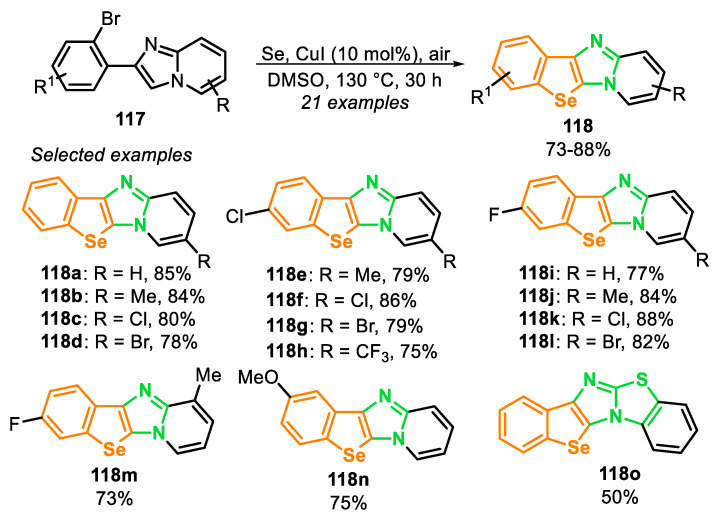
Scope of the synthesis of benzo[*b*]selenophenes fused to imidazo[1,2-*a*]-pyridines **118**.

In 2017, the synthesis of benzo[*b*]selenophene-fused imidazo[1,2-*a*]pyridines **118** through a Cu-catalyzed direct selenylation of readily available 2-(2-bromophenyl)imidazo[1,2-*a*]pyridines **117** was described [168]. The reaction involves a regioselective cleavage of C(*sp*^2^)-Br and C(*sp*^2^)-H bonds using Se powder (2 equiv) as the selenium source, in DMSO as a solvent at 130 °C for 30 h under air conditions (Scheme 77). The reaction scope was explored by employing several C6-substituted imidazo[1,2-*a*]pyridines bearing neutral, electron-donating and electron-withdrawing substituents, affording the desired benzoselenophene-fused imidazopyridines **118a**-**d** in good yields. Similar results were obtained when disubstituted 2-(2-bromophenyl)imidazo[1,2-*a*]pyridines were employed as substrates (**118e**-**n**). Additionally, 6-(2-bromophenyl)imidazo[2,1-*b*]benzo[*d*]thiazole was found to be a suitable substrate in this transformation, giving the selenylated product **118o** in 50% yield.

**Scheme 78:**
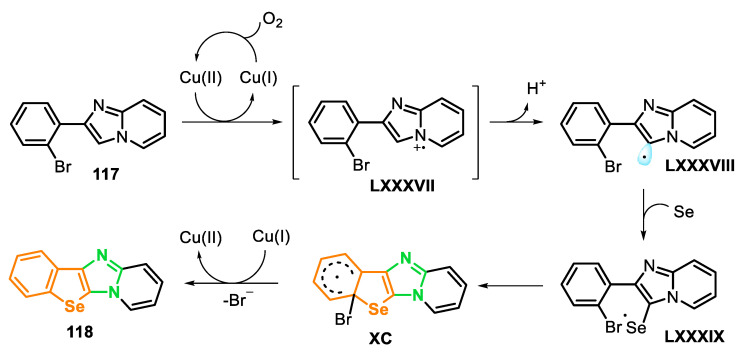
Mechanism of the synthesis of the fused benzo[*b*]selenophene **118**.

The mechanism of the reaction starts with the oxidation of the Cu(I) catalyst to the Cu(II) species in the presence of atmospheric oxygen. In the sequence, the Cu(II) species promotes an SET reaction, oxidizing the 2-(2-bromophenyl)imidazo[1,2-*a*]pyridines **117** to the cation radical **LXXXVII** and releasing Cu(I) to the reaction medium. Then, the intermediate **LXXXVII** is deprotonated to afford the vinyl radical intermediate **LXXXVIII**, which reacts with elemental selenium to give the selenium free radical species **LXXXIX**. Subsequently, the radical **LXXXIX** undergoes an intramolecular cyclization to generate the radical intermediate **XC**. Finally, a Cu(I)-mediated bromine abstraction of the radical intermediate **XC** takes place, releasing the fused benzo[*b*]selenophene **118**, one equivalent of the bromide ion and the Cu(II) species (Scheme 78).

**Scheme 79:**
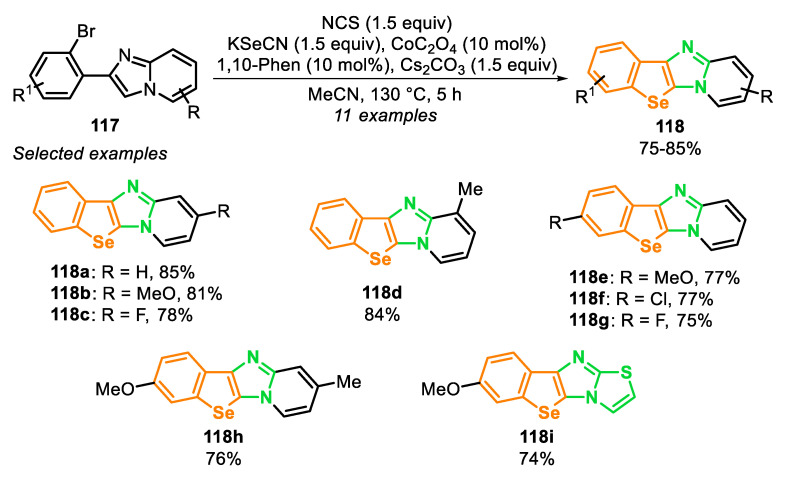
Cobalt-catalyzed synthesis of benzo[*b*]selenophene-fused imidazo[1,2-*a*]pyridines **118**.

In 2018, another approach for the synthesis of benzo[*b*]selenophene-fused imidazo[1,2-*a*]pyridines **118** was reported [169]. This protocol involves a Co-catalyzed reaction of 2-(2-bromophenyl)imidazo[1,2-*a*]pyridine **117** and potassium selenocyanate (KSeCN, 1.5 equiv) as the Se source, in the presence of *N*-chlorosuccinimide (NCS, 1.5 equiv), Cs_2_CO_3_ (1.5 equiv), 1,10-phenanthroline (10 mol%), CoC_2_O_4_ (10 mol%) and MeCN as the solvent. The reaction mixture was stirred at 130 °C for 5 h in a sealed tube (Scheme 79). The study on the reaction scope was firstly performed by employing different activated and unactivated 2-(2-bromophenyl)imidazo[1,2-*a*]pyridines **117**, giving the desired products **118a**-**h** in good yields (75-85%). The benzo[*b*]selenophene-fused imidazo[2,1-*b*]thiazole **118i** could be prepared in 74% yield, starting from a properly substituted imidazo[2,1-*b*]thiazole **117i**.

**Scheme 80:**
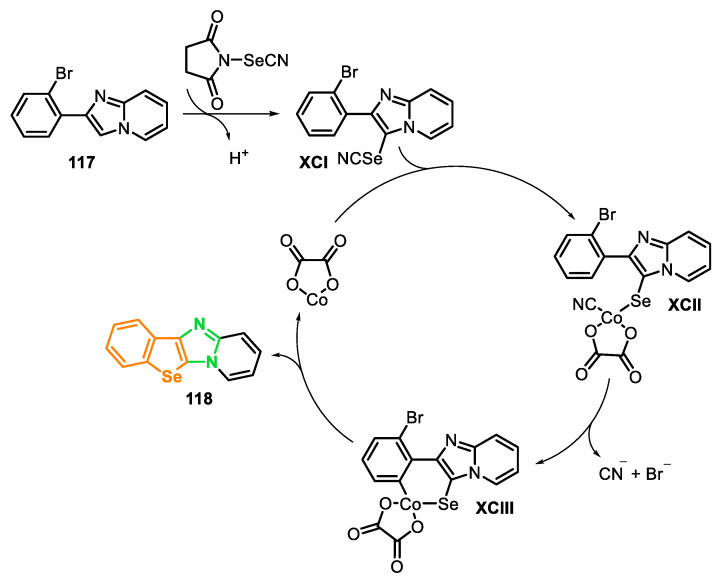
Proposed mechanism of the cobalt-catalyzed synthesis of fused benzo[*b*]selenophene **118**.

The first step in the proposed mechanism is the reaction between the 2-(2-bromophenyl)imidazo[1,2-*a*]pyridine **117** and *N*-selenocyanate-succinimide, which was formed in situ from the reaction of NCS with KSeCN, to give SeCN-substituted intermediate **XCI** (Scheme 80). Then, the intermediate **XCI** coordinates to the active cobalt catalyst, followed by an intramolecular addition to the Se-CN bond, providing the intermediate **XCII**. Then, the aryl halide displaces CN^−^, affording the intermediate **XCIII**, which undergoes a reductive elimination to regenerate the active cobalt catalyst, creating the desired product **118**.

**Scheme 81:**
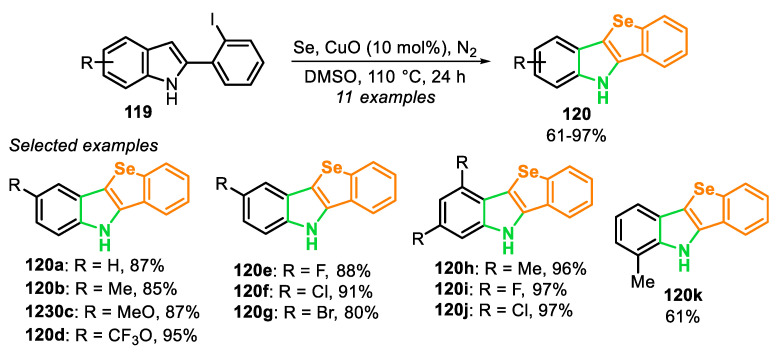
Scope of the synthesis of the benzoselenopheno[3,2-*b*]indoles **120**.

In 2016, a Cu-catalyzed intramolecular phenylselenation of 2-(2-iodophenyl)-1*H*-indoles **119** in the presence of elemental selenium for the synthesis of the benzoselenopheno[3,2-*b*]indoles **120** was described [170]. The optimal conditions involve the use of CuO (10 mol%) as a catalyst and DMSO as the solvent at 110 °C, under a N_2_ atmosphere (Scheme 81). The protocol tolerates the presence of several substituents in the indole aromatic ring: neutral (R = H), electron-donating (R = Me and MeO) and electron-withdrawing (R = CF_3_O, F, Cl and Br) groups were able to afford the corresponding fused-benzoselenophenes **120a**-**g** in good to excellent yields (80–95%). Additionally, disubstituted 2-(2-iodophenyl)-1*H*-indoles **119** afforded the respective benzoselenopheno[3,2-*b*]indoles **120h**–**j** in excellent yields (96–97%). The 2-(2-iodophenyl)-1*H*-indole **119k** (R = 7-Me); however, was less reactive, and the respective benzoselenophene **120k** was obtained in 61% yield.

### 3.5. Other Approaches to Prepare Benzoselenophenes

**Scheme 82:**
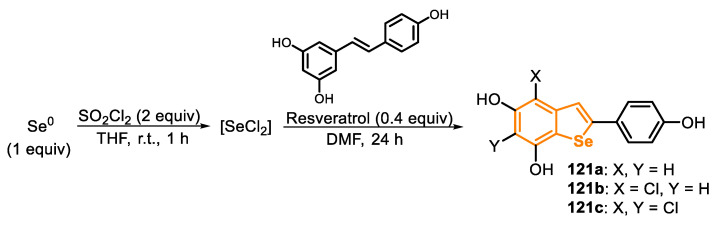
Synthesis of the benzoselenophenes from Resveratrol.

In addition to the use of types C, D, E and F precursors, there are other approaches for the synthesis of benzoselenophenes, starting from different reagents—for example, the use of resveratrol, a natural compound isolated from a broad range of plants, and present in red wines [31,171]. In these works, the authors promoted the direct selenenylation of resveratrol using SeCl_2_, wich was prepared in situ from Se(0) and SO_2_Cl_2_ in THF. The variation in the SO_2_Cl_2_ ratio resulted in different chlorinated or non-chlorinated benzo[*b*]selenophenes (Scheme 82, compounds **121a**–**c**). The authors also report that the structural modification in the Resveratrol scaffold afforded derivatives with promising pharmacological properties.

**Scheme 83:**
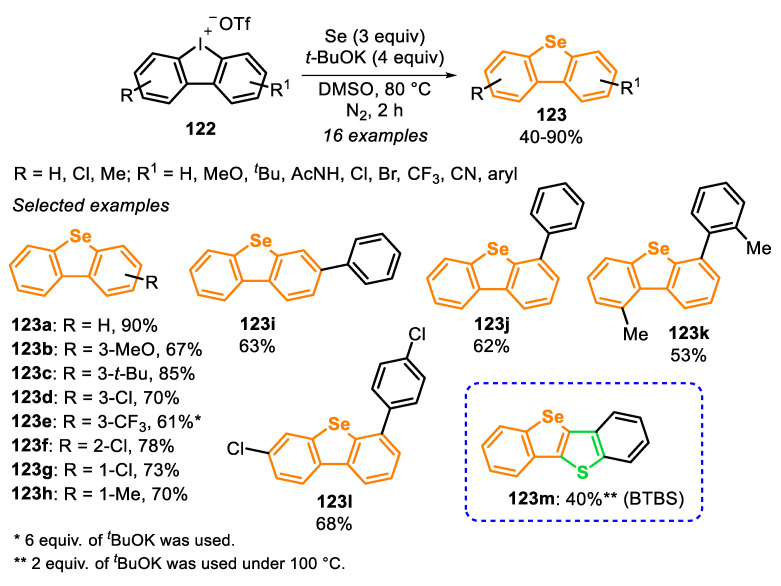
Scope of the synthesis of diaryl[*b*,*d*]selenophenes **123**.

In 2016, the synthesis of diarylannulated selenides through the selenium-iodine exchange of diaryliodonium salts **122** was reported [172]. The protocol involves the stirring a mixture of the iodonium salt, elemental selenium (3 equiv) and *t-*BuOK (4 equiv) as a strong base in DMSO as the solvent at 80 °C for 2 h, under a N_2_ atmosphere. A broad range of diaryliodonium salts **122**, bearing neutral, electron-rich and electron-deficient substituents were efficiently used as starting materials, giving the respective selenophenes **123** in good to excellent yields. The protocol was not sensitive to steric hindrance effects in the diaryliodonium salts, allowing for the synthesis of diarylannulated selenides **123i**–**l** with conjugated groups at the 3- and 4-positions. The optimal conditions were used in the synthesis of the BTBS material OFET **123m** (53% yield); however, using 2 equivalents of base at 100 °C (Scheme 83).

**Scheme 84:**
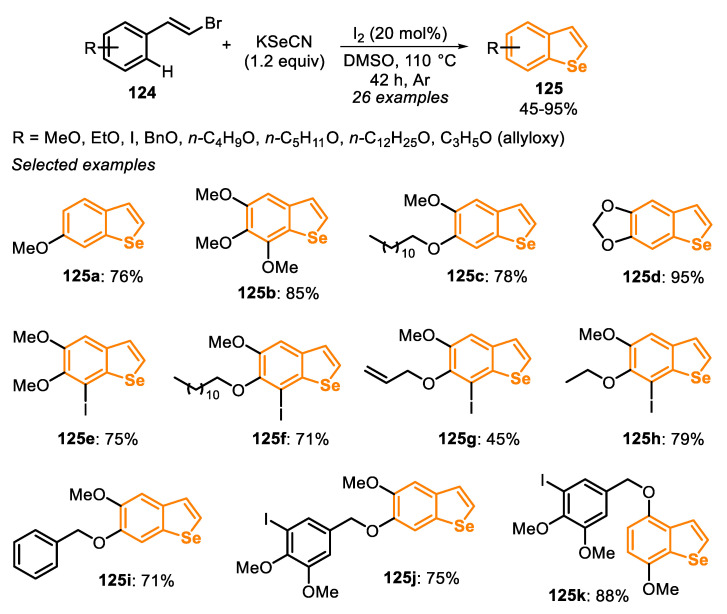
Scope of the synthesis of benzo[*b*]selenophenes **125** from styryl bromides.

In 2017, the I_2_-catalyzed selenocyanation of styryl bromides **124** with potassium selenocyanate, affording benzo[*b*]selenophenes **125** was described [173]. The best conditions for the annulation reaction was found as the stirring a mixture of the bromide **124** and KSeCN (1.2 equiv) in the presence of I_2_ (20 mol%) and DMSO as the solvent at 110 °C, under an argon atmosphere, for 48 h. Under these conditions, a total of twenty-six examples of benzoselenophenes **125** were prepared in moderate to excellent yields (45–95%). Electron-rich styryl bromides **124** substituted with methoxy, dimethoxy, trimethoxy, ethoxy, butyloxy, pentyloxy, dodecyloxy, and allyloxy groups on the aromatic ring were used as substrates in the reaction (Scheme 84).

**Scheme 85:**
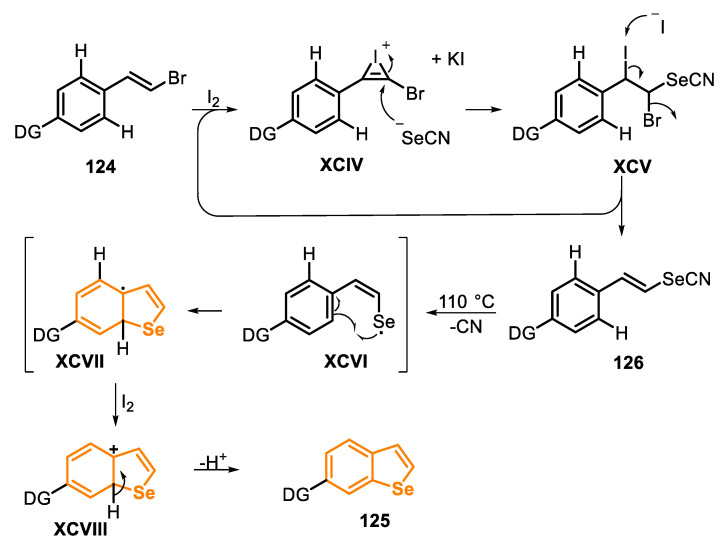
Proposed mechanism of the iodine-catalyzed synthesis of benzo[*b*]selenophene **125**.

The proposed mechanism starts with the reaction of the styryl bromide **124** with I_2_ to give a cycloiodonium ion **XCIV**, which is attacked by the nucleophile (-SeCN), affording the intermediate **XCV**. Then, the corresponding *trans*-styryl selenocyanate **126** is formed via an E_2_ mechanism, releasing I_2_ to the catalytic cycle. The next step is dependent on the presence of the electron-donor group (DG) and consists in a homolytic cleavage of the selenocyanate **126**, to generate the Se-centered radical **XCVI**. Thanks to the DG attached to the arene, the stabilized cyclic intermediate **XCVII** is generated. Finally, an oxidation-deprotonation occurs, affording the respective benzo[*b*]selenophene **125** (Scheme 85).

**Scheme 86:**
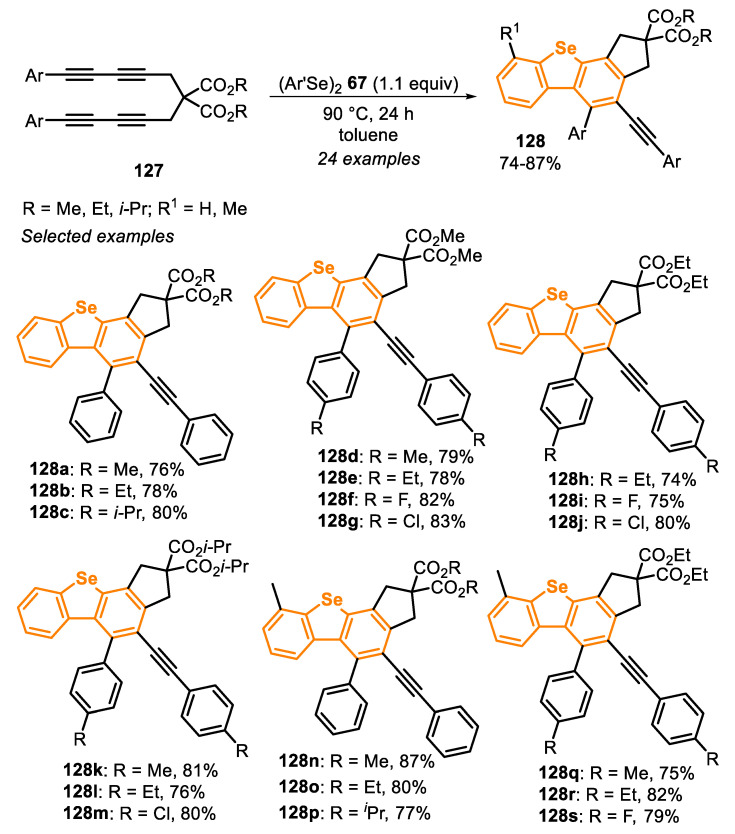
One-pot formation of fused dibenzoselenophenes **128**.

In 2017, the synthesis of multifunctionalized dibenzoselenophenes **128** through the hexadehydro-Diels-Alder (HDDA) reaction of tetrayne systems **127** with diaryl diselenides **64** was described [174]. The best results were obtained when a mixture of tetrayne **127**, and diaryl diselenide **64** (1.1 equiv), in toluene, was stirred at 90 °C for 24 h (Scheme 86). This approach is free of metals, catalysts, and additives, such as bases or oxidants, or directing groups, and worked well with a range of substrates. In general, the protocol was efficient, being suitable for tetraynes bearing neutral (H), electron-donating (Me, Et, *i-*Pr) and electron-withdrawing (F, Cl) substituents in the aryl ring. A total of twenty-four new fused dibenzoselenophenes **128** were prepared in good to excellent yields (74–87%). Good results were obtained, starting from bis-(*o*-tolyl) diselenide (Ar’ = 2-MeC_6_H_4_), and the products **128n**-**s** were isolated in 75–87% yields (Scheme 86).

**Scheme 87:**
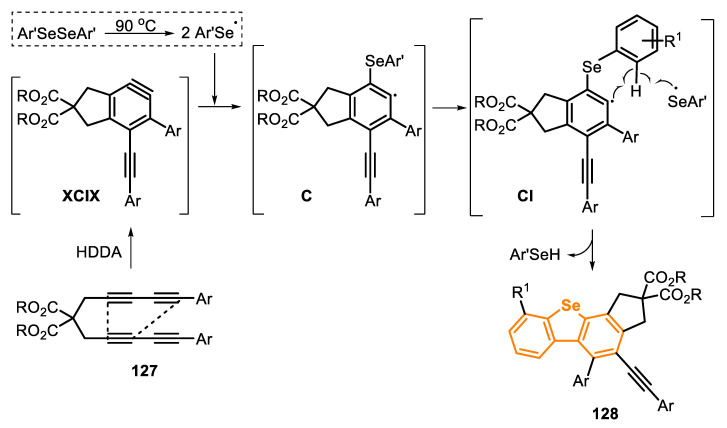
Plausible mechanism of the HDDA annulation to prepare dibenzoselenophenes **128**.

A plausible reaction mechanism was proposed for the synthesis of dibenzoselenophenes **128**, which consists in a cascade hexadehydro-Diels-Alder (HDDA) reaction, followed by a radical based C-H activation reaction. Initially, the HDDA reaction of tetrayne **127** produces an aryne as intermediate (**XCIX**), which subsequently reacts with the radical Ar’Se**^•^** (generated under heating) to give the intermediate **C**. Then, there is a homolytic α-C-H bond cleavage into the aryl group in the intermediate **C**, followed by the abstraction of an hydrogen by the free radical (Ar’Se**^•^**), giving the compound **128** (Scheme 87).

**Scheme 88:**
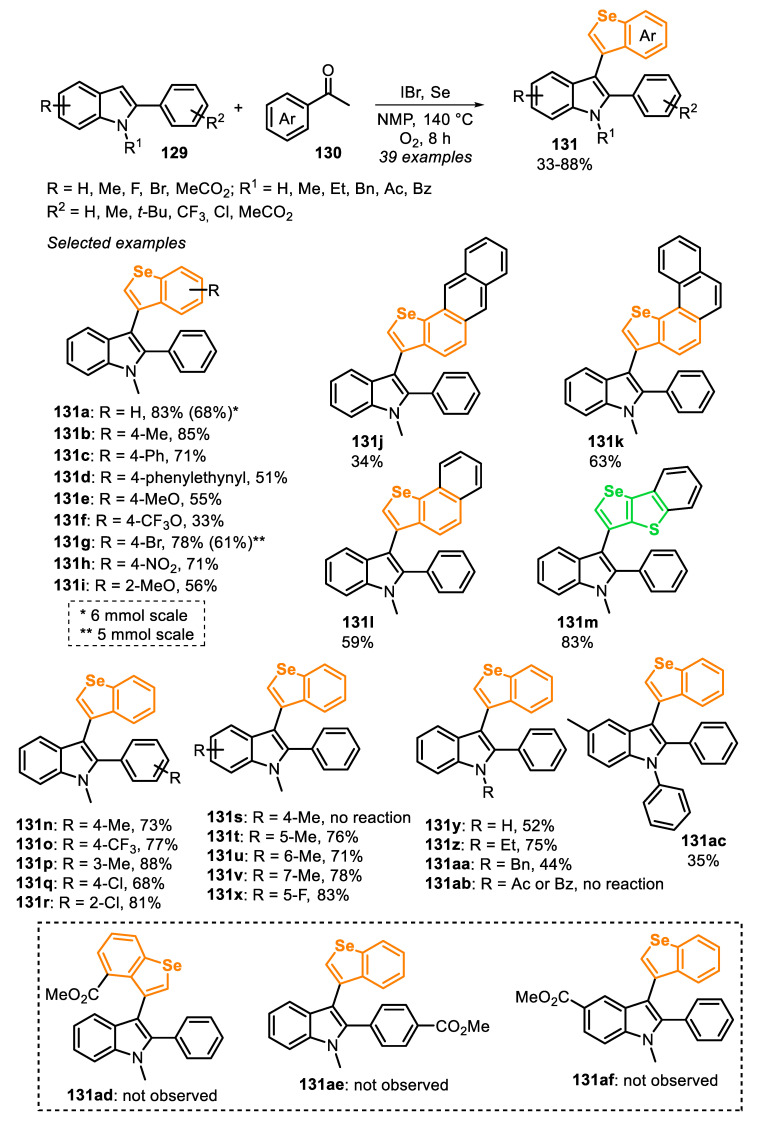
Synthesis of 3-benzo[*b*]selenophenes-2-aryl-1*H*-indoles **131**.

In 2019, a three-component cycloaddition reaction for the synthesis of benzoselenophenes **131** was developed [175]. The protocol involves the reaction of substituted indoles **129** with acetophenones **130** and Se powder, under metal-free conditions. After a systematic reaction optimization study, the best condition was established as being the stirring of a mixture of the acetophenone **130** and the indole **129** in the presence of elemental selenium (1.5 equiv), IBr (1.2 equiv) as the oxidant agent, in NMP as the solvent at 140 °C for 8 h, under O_2_ atmosphere. A study on the reaction scope was carried out, driving the formation of a diversity of thirty-nine new bis-heteroaryl compounds **131** containing both indole and benzoselenophene moieties in moderate to very good yields (Scheme 88). The optimized methodology was shown to be compatible with a range of substituted acetophenones **130** and indoles **129** bearing electron-releasing and electron-withdrawing groups. In general, the electronic effects did not affect the reaction efficiency to access the products **131**. Substrates bearing strong electron-withdrawing groups (e.g., CF_3_, NO_2_) were also compatible with the reaction conditions. However, the presence of an ester group in the aromatic ring of both, the acetophenone **130** or the indole **129** caused the total failure of the reaction, and the expected products **131ad**-**af** were not observed. The protocol was also satisfactorily scalable to 6 and 5 mmol, affording the products **131a** and **131g** in 68% and 61% yields, respectively.

**Scheme 89:**
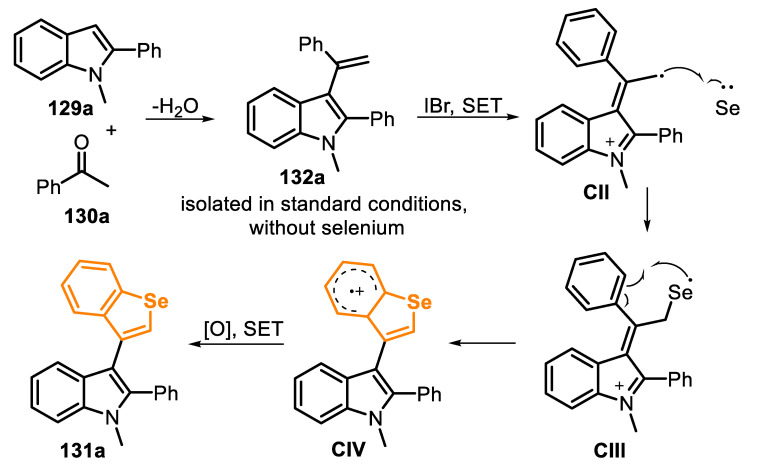
The reaction mechanism of the synthesis of selenophene **131a**.

The proposed mechanism involves, initially, a dehydrative condensation of 2-phenyl-1*H*-indole **129a** and acetophenone **130a**, giving the intermediate 3-vinyl-indole **132a**. Then, the allyl radical intermediate **CII** is generated from 3-vinyl-indole **132a** via an SET oxidation, performed by IBr. In the sequence, the intermediate **CII** reacts with elemental selenium to give the Se-centered radical intermediate **CIII**, which is converted to the intermediate **CIV**, through an intramolecular cyclization. Then, a new SET oxidation converts the radical intermediate **CIV** to the product **131a** (Scheme 89).

## 4. Fused Selenophenes and Benzoselenophenes

### 4.1. Starting from Type C Precursor

In recent years, several protocols for the synthesis of selenophenes and benzoselenophenes fused to other heterocycles, including chromenes, thiophenes, quinolines and indoles, among others, have been reported. These compounds are mainly obtained by electrophilic cyclization reactions of type C precursors, using commercially available halogenated selenium electrophiles or selenium and tellurium electrophilic species, prepared in situ from the respective diorganyl dichalcogenides using Fe-based salts.

**Scheme 90:**
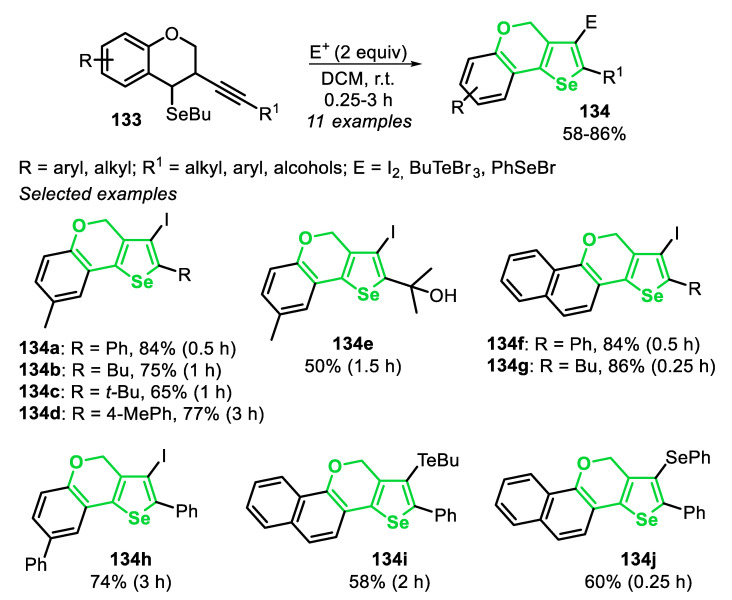
Scope of the synthesis of selenophene[3,2-*c*]chromene 3-substituted **134**.

In this context, the synthesis of 3-substituted selenonophene[3,2-*c*]chromenes **134** through the electrophilic cyclization of 3-alkynyl-4-chalcogen-2*H*-chromenes **136** using I_2_, PhSeBr and BuTeBr_3_ as electrophilic sources, and DCM as the solvent at room temperature was described (Scheme 90) [176]. Under these reaction conditions, the electrophilic cyclization of substrates **133** bearing aryl or alkyl groups at the triple bond, using I_2_ as electrophilic source, gave the products **134a**, **134b** and **134d** in good yields (75–84%). In addition, substrate **133,** bearing bulky alkyl and propargyl alcohols groups, afforded the desired products **134c** and **134e** in lower yields (65% and 50%, respectively). Besides that, the protocol was explored using different substituents in the chromene ring, such as naphthyl and aryl ones, producing the desired fused selenophenes **134f**-**h** in good yields (74–86%). When the cyclization reaction was carried using the BuTeBr_3_ and PhSeBr as electrophiles, the corresponding 3-chalcogen-selenophene[3,2-*c*]chromene **134i** and **134j** were obtained in similar yields after 2 h and 15 min, respectively.

**Scheme 91:**
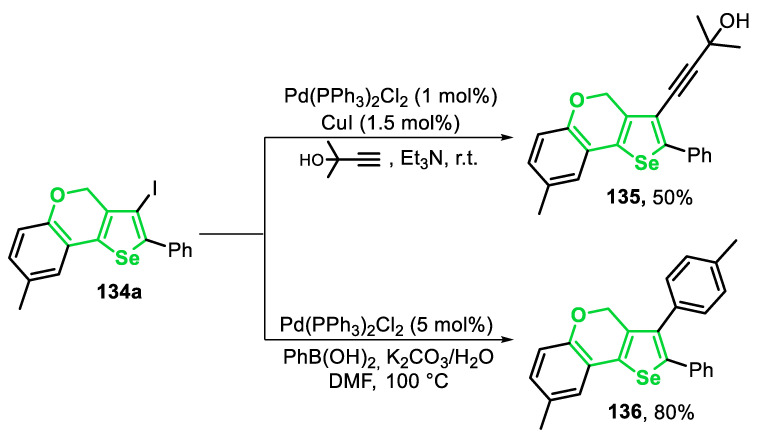
Reactivity of 3-iodo-selenophene[3,2-*c*]chromene **134a** toward Sonogashira and Suzuki cross-couplings.

The reactivity of the 3-iodo-selenophene[3,2-*c*]chromenes **134** was evaluated in the Pd-catalyzed cross-coupling reaction. Thus, the 3-iodo-selenophene[3,2-*c*]chromenes **134a** reacted satisfactorily with terminal alkynes and boronic acid, to give the Sonogashira and Suzuki-type products **135** and **136** in 50% and 80% yields, respectively (Scheme 91).

**Scheme 92:**
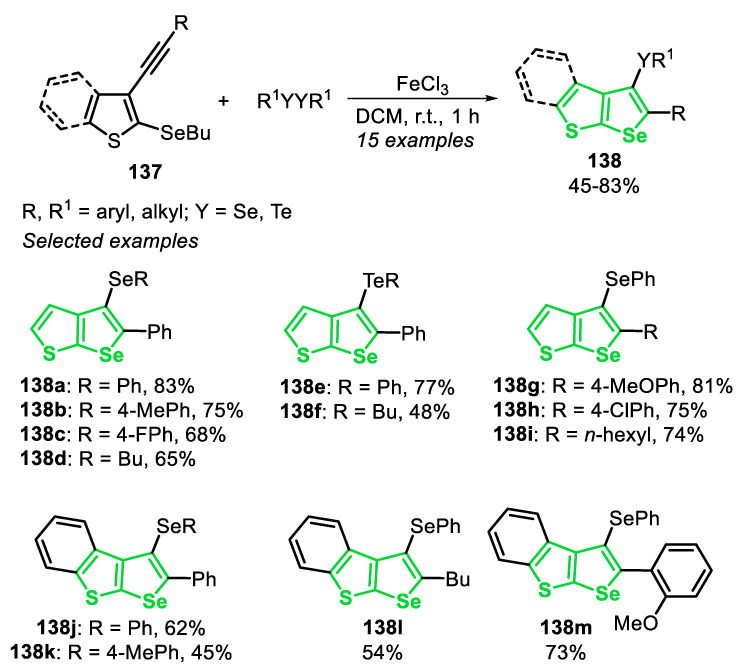
Scope of the synthesis of 3-organochalcogen-selenophen[2,3-*b*]thiophenes **138**.

In 2013, a FeCl_3_/diorganyl dichalcogenides-promoted intramolecular cyclization of 2-organochalcogen-3-alkynylthiophenes **137**, for the synthesis of selenophene[2,3-*b*]thiophenes **138**, using DCM as the solvent at room temperature and under air atmosphere was developed (Scheme 92) [177]. The scope of the reaction was firstly evaluated by using alkyl and diaryl diselenides bearing neutral, electron-donor and electron-withdrawing substituents in the aromatic ring, producing the desired 3-organoselanyl-selenophene[2,3-*b*]thiophenes **138a**–**d** in moderate to good yields (65–83%). When dialkyl and diaryl ditelluride were used as substrates, similar results were obtained. Dibutyl dichalcogenides were less reactive than the diaryl analogues (Scheme 92, products **138d** and **138f** vs. **138a**–**c** and **138e**). This behavior was attributed by the authors to the presence of β-hydrogen atoms in the butyl group bonded to the chalcogen atom, which may favor a decomposition via a telluroxide or selenoxide *syn*-elimination during the purification or work-up process. The 3-Alkynylbenzothiophenes were less reactive in the cyclization reaction, affording the products **138j**–**l** in 62%, 45% and 54% yields, respectively. A competitive cyclization reaction between the nucleophilic oxygen from an *o*-methoxyl substituent in the aryl group of the alkyne and a selenium nucleophile directly bonded to the thiophene ring was not observed. For instance, the selenophene **138m** formed by a *Se*-cyclization, was the only product observed in the reaction of the alkyne **137m**, with the complete absence of furan derivatives.

**Scheme 93:**
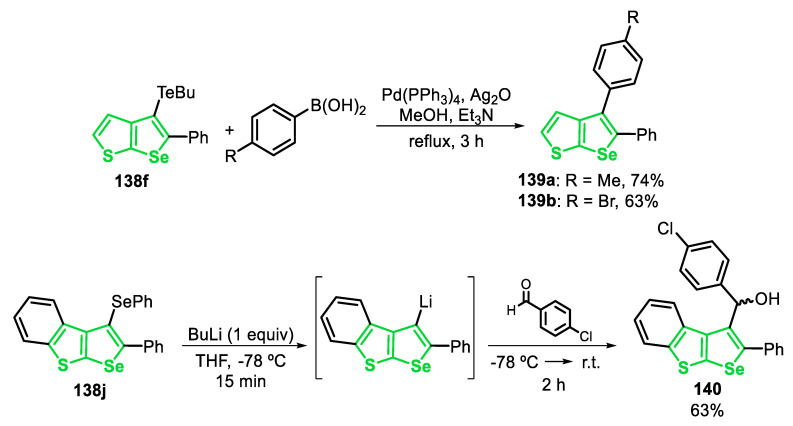
Suzuki and Li-Se exchange reactions of fused selenophenes **138f** and **138j**.

The reactivity of 3-organochalcogenyl-selenophene[2,3-*b*]thiophenes **138** was explored in the Suzuki Pd-catalyzed (YR^1^ = TeBu) and Li-Se exchange (YR^1^ = SePh) reactions (Scheme 93). Under the Suzuki reaction conditions, 4-butyltelluro-selenophene[2,3-*b*]-thiophene **138f** underwent the cross-coupling reaction smoothly, by the exposure to aryl boronic acids bearing electron-rich and electron-deficient substituents, affording the desired products **139a** and **139b** in 74% and 63% yields, respectively. In addition, the 4-phenylselanyl-selenophene[2,3-*b*]thiophene **138j** was submitted to the Li-Se exchange reaction, with butyllithium in THF at −78 °C, giving the lithium intermediate, which was trapped with 4-chlorobenzaldehyde, affording the secondary alcohol **140** a 63% yield after 2 h.

**Scheme 94:**
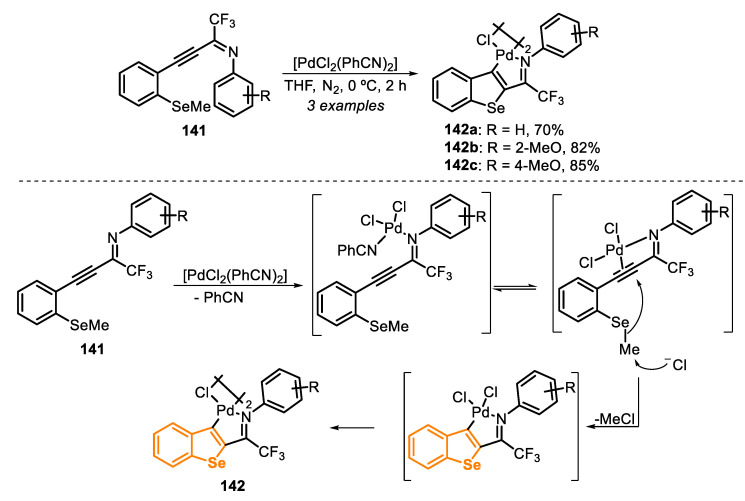
Scope and mechanism of the synthesis of benzoselenophene-based palladacycles **142**.

In 2014, was described the intramolecular hetero-cyclization and cyclopalladation of selenoalkyne-substituted propargyl imines **141**, for the synthesis benzoselenophene[2,3-*c*]pyridinones **142 [178]**. In order to promote this reaction, the palladations of *ortho*-selenoanisole perfluoro propargyl imines **141** was performed using a stoichiometric amount of dichoro-bis(triphenylphosphine)palladium(II) in dry THF, for 2 h at 0 °C, producing the respective benzoselenophene-based palladacycles **142a**-**c** in 70–85% yields (Scheme 94). A plausible reaction mechanism for the formation of benzoselenophene-based palladacycles involves the coordination of the palladium catalyst to the nitrogen atom, followed by an interaction of the triple bond to the palladium atom. After, the electron-rich selenium atom attacks the activated triple bond, with concomitant removal of the methyl group via an S_N_2 nucleophilic displacement, which promotes it by the chloride anion present in the reaction medium, to give the product **142** (Scheme 94).

**Scheme 95:**
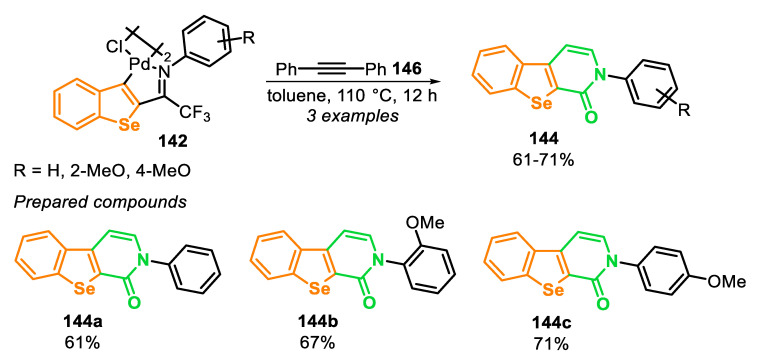
Synthesis of benzoselenophene[2,3-*c*]pyridinones **144**.

These palladacycles were then subjected to the insertion of diphenyl acetylene (DPA) **143** into the Pd-C bond, in refluxing toluene for 12 h, affording three different benzoselenophene[2,3-*c*]pyridinones **144a**-**c** in 61–71% yields (Scheme 95).

**Scheme 96:**
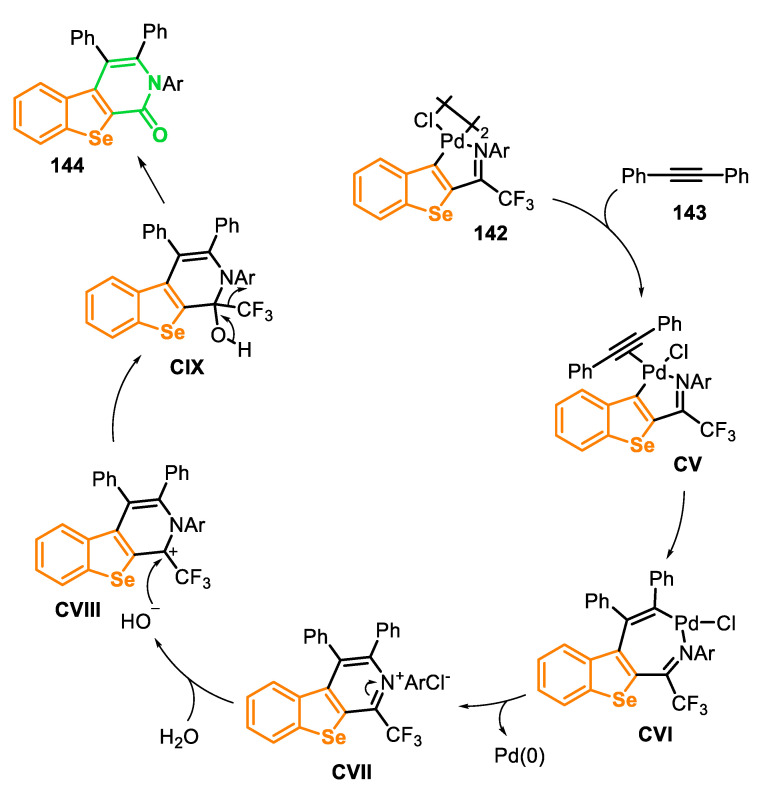
Mechanism of the synthesis of benzoselenophene[2,3-*c*]pyridinone **144**.

The proposed reaction mechanism to prepare the fused benzoselenophene derivatives **144** involves, firstly, the generation of the seven-membered ring intermediate **CVI**, after the coordination of the C-C triple bond to the Pd coordinating sphere (intermediate **CV**). Then, the insertion into the Pd-C bond occurs, where the Pd atom interacts with the alkynyl carbon followed by heterolytic Pd-C cleavage and ring closure to form the intermediate **CVII**. The formation of benzoselenophene[2,3-*c*]pyridinones **144** rather than the expected pyridinium salt may be attributed to the presence of the CF_3_ group bonded to the pyridinium ring in **CVII**. The carbon atom attached to CF_3_ is more electrophilic because of the highly electronegative nature of CF_3_ group, which in turn is susceptible to a nucleophilic attack by the hydroxyl ion to the intermediate **CVIII**. Finally, the intermediate **CIX** undergoes an oxidation to form the carbonyl group in **144** (Scheme 96).

**Scheme 97:**
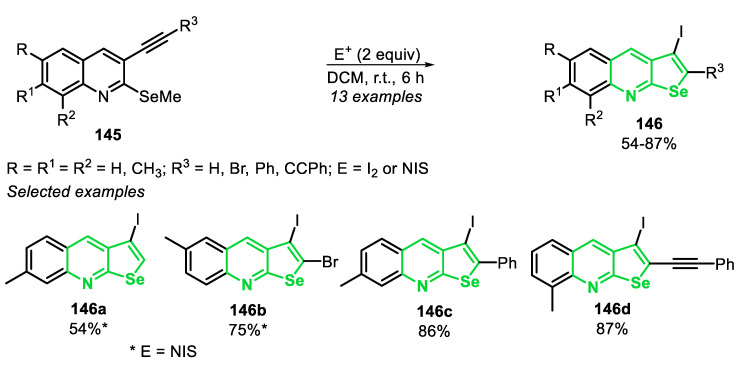
Scope of the synthesis of selenopheno[2,3-*b*]quinolines **146**.

In 2018, the synthesis of selenopheno[2,3-*b*]quinoline **146** through the iodo-cyclization reaction of 3-alkynyl-2-(methylseleno)quinolines **145** in DCM at room temperature for 6 h, using I_2_ or NIS as an electrophilic source, was reported [179]. Under these reaction conditions, the corresponding fused selenophenes **146a** and **146b** were synthetized in 54% and 75% yields, respectively, by the iodocyclization of 3-alkynyl-2-(methylseleno)quinolines **145a** (R = H) and **145b** (R = Br), using *N*-iodosuccinimide (NIS) as an electrophilic source. Similarly, 2-substituted selenopheno[2,3-*b*]quinolines, **146c** and **146d**, bearing phenyl and phenylethynyl groups, were obtained in 86% and 87% yields, respectively, using I_2_ as an electrophile (Scheme 97).

**Scheme 98:**
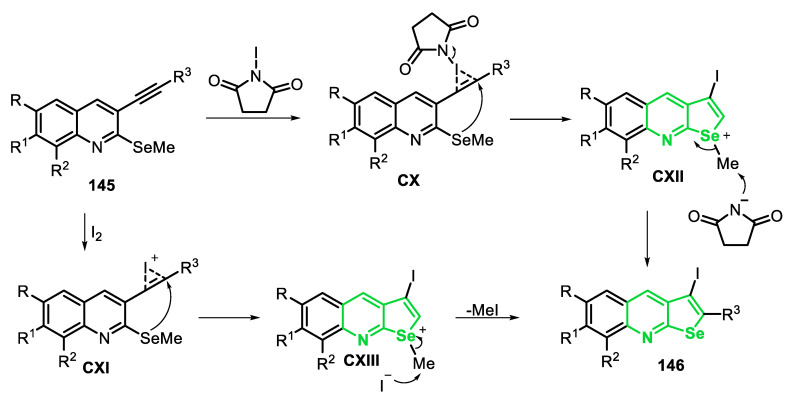
Proposed mechanism for the synthesis of selenopheno[2,3-*b*]quinoline **146**.

To investigate the reaction mechanism of the iodocyclization of 3-alkynyl-2-(methylseleno)quinolines **145**, the authors performed density functional theory (DFT) calculations. Initially, the mechanism involves the addition of electrophile NIS or I_2_ to the C-C triple bond of the 3-alkynyl-2-(methylseleno)quinoline **145**, producing the three-membered cyclic intermediate iodonium **CX** or **CXI**. In the sequence, an *anti*-attack of the Se atom to the activated triple bond of **CX** or **CXI** occurs, producing the selenonium salt **CXII** or **CXIII**, via a 5-*endo-dig* cyclization (Scheme 98). Subsequently, an S_N_2 displacement of the methyl group, linked to the Se atom by the nucleophile present in the reaction medium, creates the selenopheno[2,3-*b*]quinoline **146** and MeI or *N*-methylsuccinimide as byproducts.

**Scheme 99:**
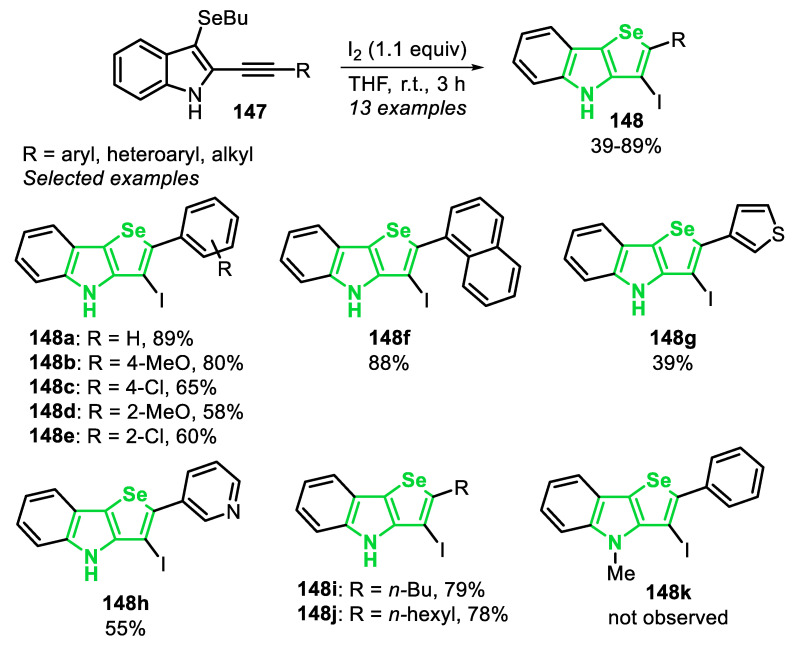
Scope of the synthesis of 3-iodo-4*H*-selenopheno[3,2-*b*]indoles **148**.

A similar protocol was developed this year for the synthesis of 3-iodo-selenophene-fused indoles **148**, through an intramolecular electrophilic cyclization of 3-organoselanyl-2-alkynylindoles **147** using I_2_ in THF, at room temperature for 3 h (Scheme 99) [180]. The versatility of this protocol was firstly evaluated by the reaction of alkynylindoles **147** bearing neutral, electron-rich, and electron-deficient aryl groups, as well as the naphtyl derivative, producing the 3-iodo-selenophene-fused to indoles **148a**-**f** in moderate to good yields. In these reactions, lower efficiency was observed starting from *ortho*-methoxy- and *ortho*-chloro-substituted arylalkynes, probably due to steric effects, and **148d** and **148e** were obtained in 58% and 60% yields, respectively. When heteroaryl groups directly bonded to the alkyne were employed as substrates, the desired cyclized products **148g** and **148h** were afforded in 39% and 55% yields, respectively. Good results were obtained starting from *n*-butyl- or *n*-hexyl-substituted alkynes, affording the fused selenophenes **148i** and **148j** in 79% and 78% yields, respectively. The reaction did not work when the *N*-methyl indole **147k** was employed as a substrate, and the expected cyclized product **148k** could not be obtained.

**Scheme 100:**
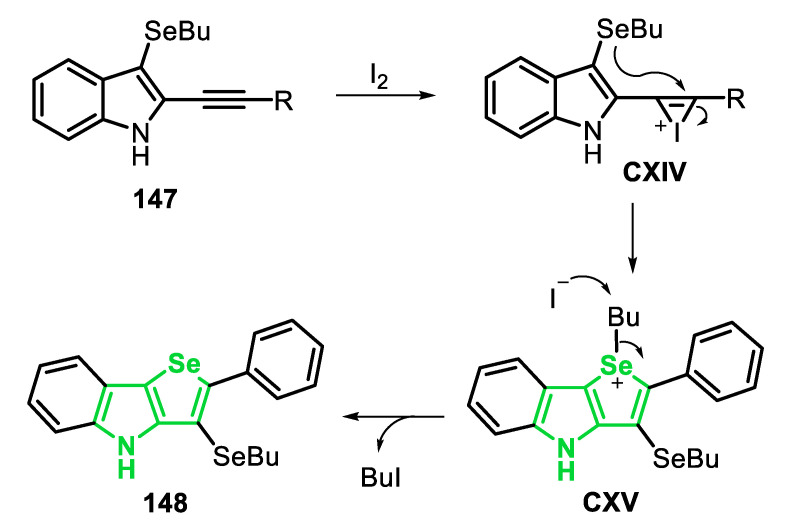
Mechanism of the synthesis of 3-iodo-4*H*-selenopheno[3,2-*b*]indoles **148**.

The mechanism of the formation of 3-iodo-4*H*-selenopheno[3,2-*b*]indole **148** involves the initial activation of the C-C triple bond of the substrate **147**, via the formation of the iodonium ion **CXIV**, which was obtained by the coordination of the triple bond to the I_2_. In the sequence, an *anti*-nucleophilic attack of the Se atom to the activated triple bond gives the selenium salt **CXV**, through a selective intramolecular 5-*endo-dig* cyclization. The iodide anion, present in the reaction mixture, promotes the S_N_2 displacement of the butyl group bonded to selenium, affording the desired fused selenophene **148** and butyl iodide (Scheme 100).

**Scheme 101:**
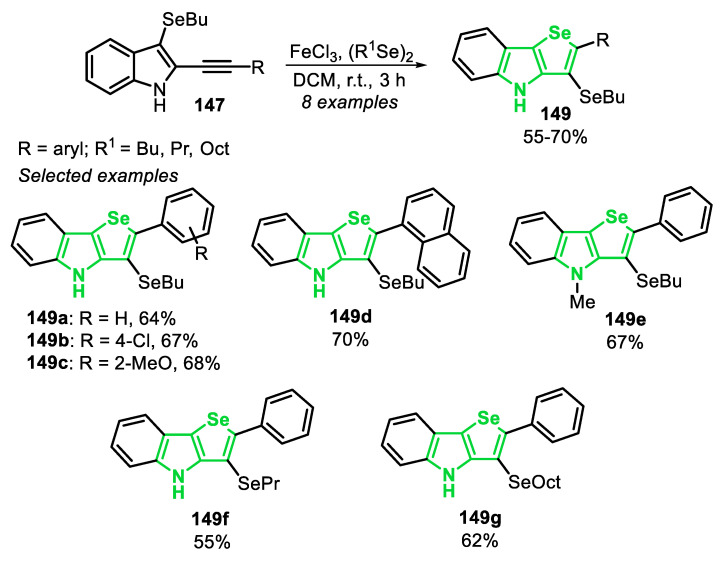
Scope of the synthesis of 3-(alkylselanyl)-4*H*-selenopheno[3,2-*b*]indoles **149**.

In the same work, the authors described the synthesis of 3-(alkylselanyl)-selenophene-fused indoles **149** through the cyclization reaction of 3-butylselanyl-2-alkynylindoles **147** using a selenium electrophilic species generated in situ. In this reaction, a mixture of the substrate **147**, FeCl_3_ and dibutyl diselenide in DCM was stirred at room temperature for 3 h. By this protocol, 3-butylselanyl-2-alkynylindoles bearing pendant naphthyl, neutral, electron-poor and electron-rich aryl groups in the alkyne were converted to the respective 3-butylseleno-selenonophene-fused indoles **149a**-**d** in 64%–70% yields. Good results were obtained starting from *N*-protected 3-(butylselanyl)indole and other dialkyl diselenides, and compounds **149e**-**g** were prepared in 55%–67% yields (Scheme 101).

### 4.2. Starting from Type D Precursors

**Scheme 102:**
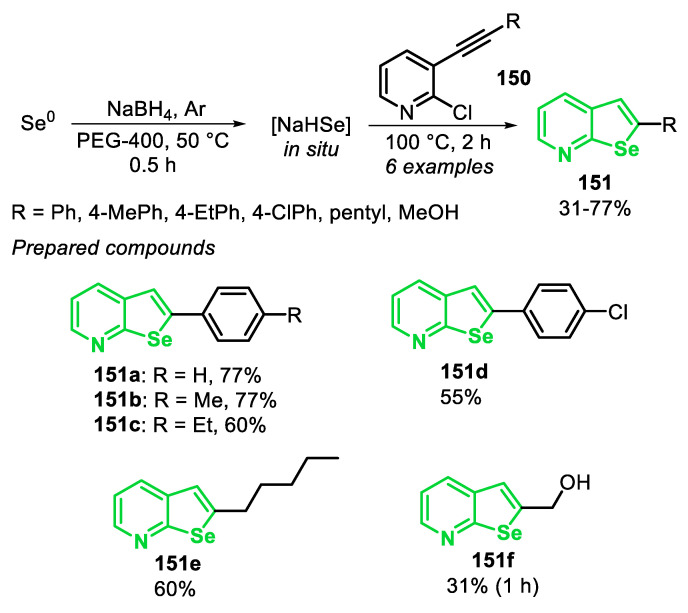
Scope of the synthesis of selenophenes fused to pyridines.

Recently, some of us [181] described the synthesis of 2-organylselenopheno[2,3-*b*]pyridines **151** by the insertion of Se-based nucleophilic species into 2-chloro-3-(organylethynyl)pyridines **150**, followed by an intramolecular cyclization reaction. The nucleophilic selenide was generated in situ by the reduction of elemental selenium using the NaBH_4_/PEG-400 system (Scheme 102). The experimental procedure for the synthesis of selenophenes fused to pyridines **151** consists of two steps: firstly, Se powder (0.3 mmol) and NaBH_4_ (0.9 mmol) are poured into a vessel containing PEG-400 (2 mL), under an argon atmosphere at 50 °C, and are stirred at this temperature for approximately 0.5 h (the color of the mixture changes from black to white). Then, in a second step, 2-chloro-3-(organylethynyl)pyridine **150** (0.25 mmol) is added, and the temperature is raised to 100 °C for an additional 2 h. The generality of this protocol was evaluated in the reaction of differently substituted 2-chloro-3-alkynylpyridines **150**, including those with electron-rich and electron-poor arenes and alkyl groups. In a general way, the desired fused-selenophenes **151a**-**e** were obtained in moderate to good yields (55–77% yield). One exception was the 2-chloropyridine **150f**, containing a propargyl alcohol at the 3-position, which afforded the corresponding selenopheno[2,3-*b*]pyridin-2-ylmethanol **151f** in only 31% yield after 1 h.

**Scheme 103:**
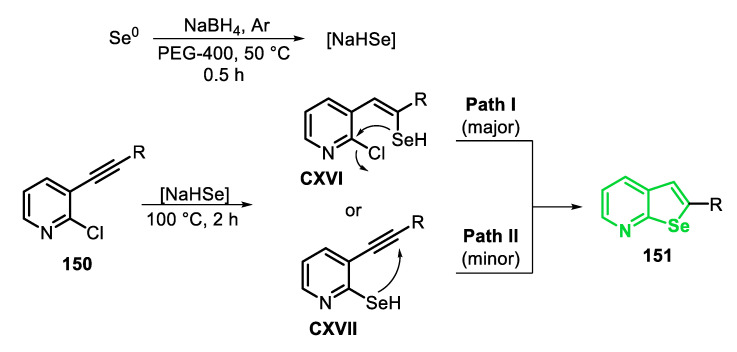
Proposed mechanism of the synthesis of **151**.

After some control experiments, the authors were able to propose a mechanism for the synthesis of 2-organylselenopheno[2,3-*b*]pyridines **151**. The first step is the attack of the pre-formed NaHSe to the C-C triple bond of the alkynylpyridine **150**, generating the intermediate **CXVI**, followed by an intramolecular S_N_Ar reaction (Path I). However, the reaction may also go through another pathway, which consists in a nucleophilic attack (S_N_Ar) of the selenium anion to the 2-chloroalkylpyridine **150**, followed by an intramolecular hydroselenation reaction of the triple bond (Path II) (Scheme 103).

**Scheme 104:**
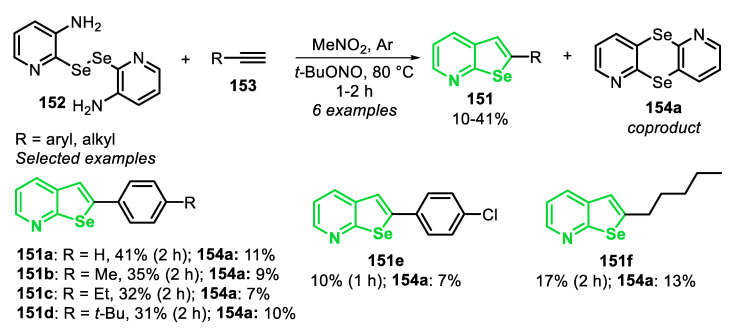
Scope of the synthesis of 2-organylselenopheno[2,3-*b*]pyridines **151**.

In addition, some of us [182] reported the synthesis of 2-organylselenopheno[2,3-*b*]pyridines **151**, starting from bis(3-amino-2-pyridyl)diselenide **152** and aryl- or alkylacetylenes **153**, in the presence of *t*-BuONO as a nitrosating agent in MeNO_2_, at 80 °C under argon atmosphere for 2 h (Scheme 104). The scope of the protocol was investigated by employing different aryl- and alkylacetylenes **153** in the reaction with bis(3-amino-2-pyridyl)diselenide **152** under the optimized conditions. Thus, when the reaction was performed using arylacetylenes with electron-donating groups at the *para*-position of the aromatic ring, the respective fused selenophenes **151b**-**d** were obtained in similar yields than that using the neutral phenylacetylene (**151a**). In contrast, the presence of the electron-withdrawing Cl-substituent at the aromatic ring caused a fast consumption of the starting material; however, the product **151e** was obtained in only 10% yield, due to the formation of many byproducts. When heptyne was employed as substrate, the respective 2-pentylselenopheno[2,3-*b*]pyridine **151f** was obtained in 17% yield after 2 h. In all cases 1,6-diazaselenanthrene **154a** was formed as a coproduct in yields ranging from 7% to 13%.

**Scheme 105:**
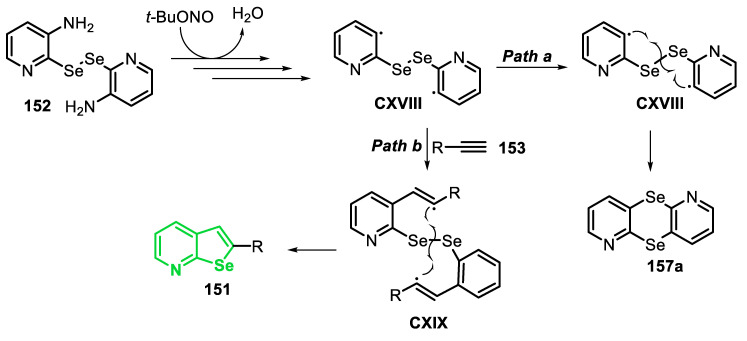
Mechanism of the synthesis of 2-organylselenopheno[2,3-*b*]pyridines **151**.

The proposed mechanism of the synthesis of the fused selenophenes **151** initiates with the reaction between pyridylamine **152** and *tert*-butyl nitrite, resulting in the radical species **CXVIII**. From the intermediate **CXVIII**, the reaction can follow two different paths, which depend on the interaction with the acetylene. In Path a, the reaction occurs without the presence of acetylene **153**, through two intramolecular radical reactions to afford 1,6-diazaselenanthrene **154a**. Alternatively, the reaction could follow the Path b, with the reaction between the radical intermediate **CXVIII** and the acetylene **153**, affording the vinyl radical **CXIX**, which reacts by an intramolecular homolytic substitution at the Se atom, leading to the final product **151** (Scheme 105).

**Scheme 106:**
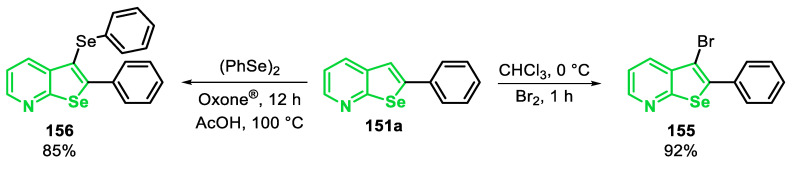
Synthetic applications for compound **151a**.

To demonstrate the synthetic applicability of the prepared 2-phenylselenopheno[2,3-*b*]pyridines **151a**, this compound was reacted with Br_2_, in dry chloroform at 0 °C, affording, after 1 h, 3-bromo-2-phenylselenopheno[2,3-*b*]pyridine **155** in 92% yield, which is a suitable substrate in several classic cross-coupling reactions, such as Suzuki–Miyaura, Sonogashira and Negishi. In addition, the compound **151a** reacted with diphenyl diselenide, in the presence of Oxone^®^, in AcOH at 100 °C to give the 2-phenyl-3-(phenylselanyl)selenopheno[2,3-*b*]pyridine **156** in 85% yield after 12 h (Scheme 106).

### 4.3. Starting from Type E Precursors

**Scheme 107:**
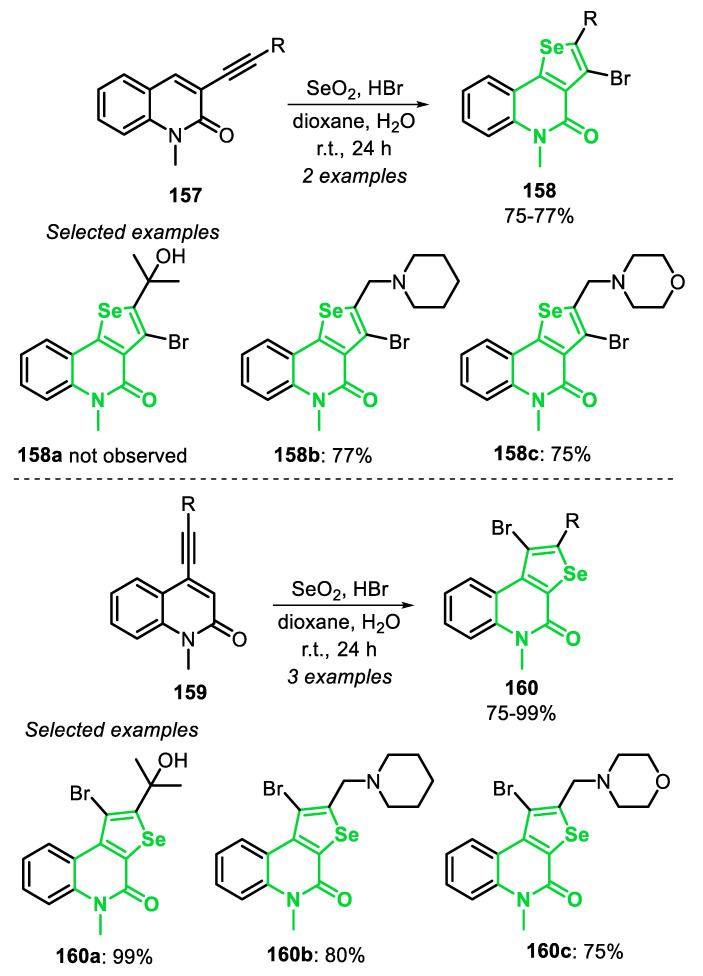
Scope of the synthesis of selenopheno[3,2-*c*]- and selenopheno[2,3-*c*]quinolones.

In 2014, the synthesis of selenophenes fused to quinolines **158** and **160** through the selenobromination of alkynilquinolones **157** and **159**, respectively, was described [183]. This reaction was promoted by selenium tetrabromide in dioxane as the solvent, at room temperature for 24 h. The selenium(IV) species was generated in situ from SeO_2_ and HBr (Scheme 107). The optimal conditions were applied in the *selane*-Friedel-Crafts cyclization of 3-alkynylquinolones **157b-c** to afford the respective selenophene[3,2-*c*]quinolones **158b** and **158c** in 77% and 75% yields. When the 3-alkynylquinolone **157a**, containing the hydroxyl group, was employed as a substrate, the expected product **158a** was not formed. Better results, however, were obtained starting from the 4-alkynylquinolone isomers **159**, for which the same conditions were afforded the selenopheno[2,3-*c*]-quinolones **160a**-**c** in 75–99% yield, including the fused selenophene **160a** containing the hydroxyl group that was selectively formed in a nearly quantitative yield. In the same work, the compounds were tested for their cytotoxic activity in vitro against several tumor cells lines. The 3-Bromo-5-methyl-2-(piperidinylmethyl)selenopheno[3,2-*c*]quinolone (**158b**) presented a pronounced cytotoxic activity against the human uterine sarcoma MES-SA.

**Scheme 108:**
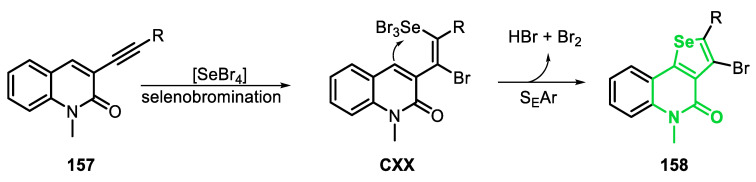
Proposed mechanism of the synthesis of selenopheno[3,2-*c*]quinolones.

The proposed mechanism involves a cyclization reaction, followed by two sequential processes. The first step involves an *anti*-addition of selenium tetrabromide to the C-C triple bond of the 3-alkynylquinolone **157**, affording the selenotribromide intermediate **CXX**. Then, an intramolecular cyclization through a S_E_Ar mechanism occurs, giving the selenopheno[3,2-*c*]quinolones **158**, while one equivalent of HBr and Br_2_ is released. The formation of selenopheno[2,3-*c*]quinolones **160** from the 4-alkynylquinolone **158** proceeds by the same mechanism (Scheme 108).

**Scheme 109:**
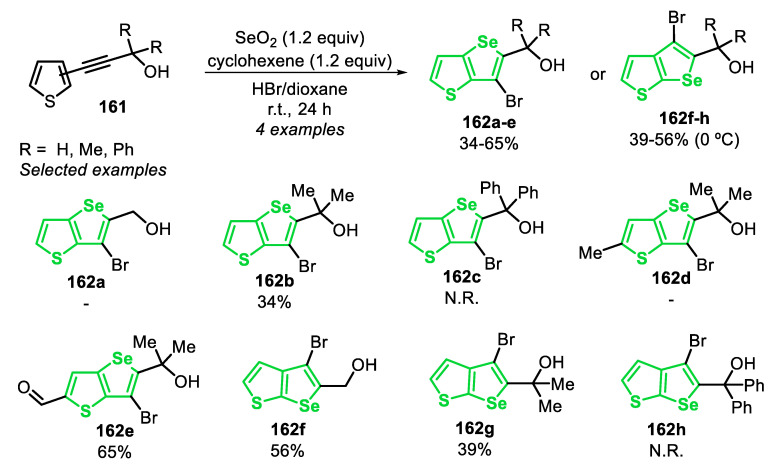
Scope of the synthesis of selenopheno[3,2-*b*]- and selenopheno[2,3-*b*]thiophenes **162**.

In 2014, a close-related procedure was developed, the selenobromination of thienylpropargyl alcohols **161**, using cyclohexene as an additive, for the synthesis of selenopheno[3,2-*b*]- and selenopheno[2,3-*b*]thiophenes **162**, when the reaction is conducted at room temperature or at 0 °C, respectively (Scheme 109) [164]. The selenium(IV) bromide was prepared in situ by the reaction of SeO_2_ with an appropriate amount of concentrated HBr. The reaction using the unsubstituted thienylpropargyl alcohol **161a** (R = H) produced a complex mixture of products besides the expected selenophene **162a**, including the corresponding dibromo-derived and the bromination of the α-position of the thiophene ring. The cyclization of dimethyl-substituted thienylpropargyl alcohol **161b** (R = Me) led to the formation of the selenopheno[3,2-*b*]thiophene derivative **162b** in 34% yield. The cyclization product **162c** was not detected, probably due to the steric hindrance of the phenyl groups in the starting material. A low reactivity was also observed when the α-methyl-substituted thienylpropargyl alcohol **161d** was the substrate, and only a trace amount of **162d** was observed by GC/MS, with the corresponding dibromo derivative being formed as the main product. The presence of the strongly electron-withdrawing formyl group in the pendant thienyl ring positively influenced the reaction, and the fused selenophene **162e** was afforded a 65% yield, without formation of the corresponding dibromo derivative. The selenopheno[2,3-*b*]thiophenes **162f** and **162g** were obtained in 56% and 39% yields, respectively, while the diphenyl-substituted selenophene (**162h**) was not formed, reinforcing the influence of steric effects in the reaction.

**Scheme 110:**
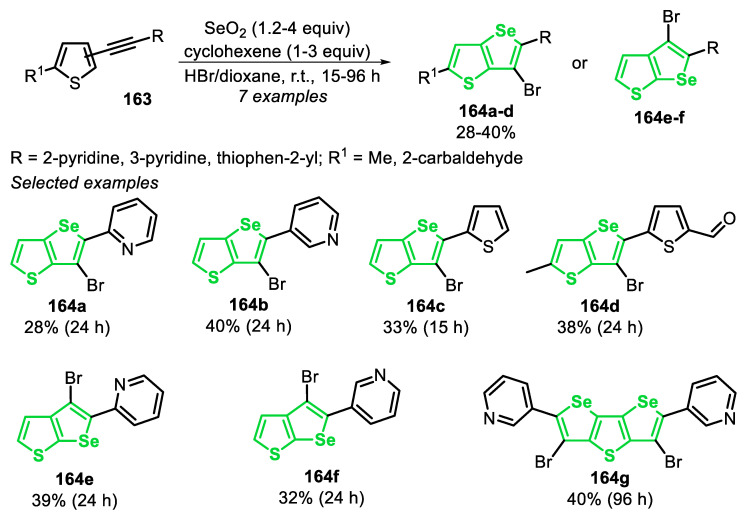
Scope of the synthesis of the selenophenothiophenes **164**.

This strategy to generate SeBr_4_ in situ was used in 2015 by the same group [165], in the synthesis of selenophenothiophenes **164**, through the cyclization of diheteroarylalkynes **163**. The cyclization reactions were regiospecific in all cases, affording the corresponding selenophenothiophene derivatives **164a**–**g** in modest yields (28–40%). For the synthesis of **164a** (R = 2-pyridinyl) and **164b** (R = 3-pyridinyl), the optimal reaction conditions were achieved when 1.2 equiv. of SeO_2_ and 1.5 equiv. of cyclohexene were used in the presence of HBr in dioxane at room temperature. The low yields observed in all the cases could be attributed to the competing α-bromination of the thiophene ring. In the reaction starting from bis(thiophen-2-yl)ethyne, the expected product **164c** was obtained in 33% yield. In this case, cyclohex-2-enone was used as a bromine scavenger, reducing the α-bromination of the thiophenes. In the case of unsymmetrically substituted derivative **163d** (R = 5-thiophenaldehyde, R^1^ = Me), the α-positions of the thiophene ring are blocked, preventing their bromination. However, a large excess of SeO_2_ (4 equiv) and cyclohexene (3 equiv) were necessary to afford the corresponding cyclization product **164d** in only 38% yield after 24 h of reaction. It was observed that the bromination of the C-C triple bond of the starting dihetarylalkyne **163d** was quite pronounced. This approach was suitable to prepare the selenopheno[2,3-*b*]thiophene derivatives **164e** and **164f**, which were obtained in 39% and 32% yields after 24 h. The cyclization of 2,5-bis(pyridin-3-ylethynyl)thiophene (**163g**) was a successful example of bis-cyclization, giving **164g** in 40% yield after 96 h. This reaction was performed in the presence of 3 equivalents of SeO_2_ and 2.4 equivalents of cyclohexene (Scheme 110).

**Scheme 111:**
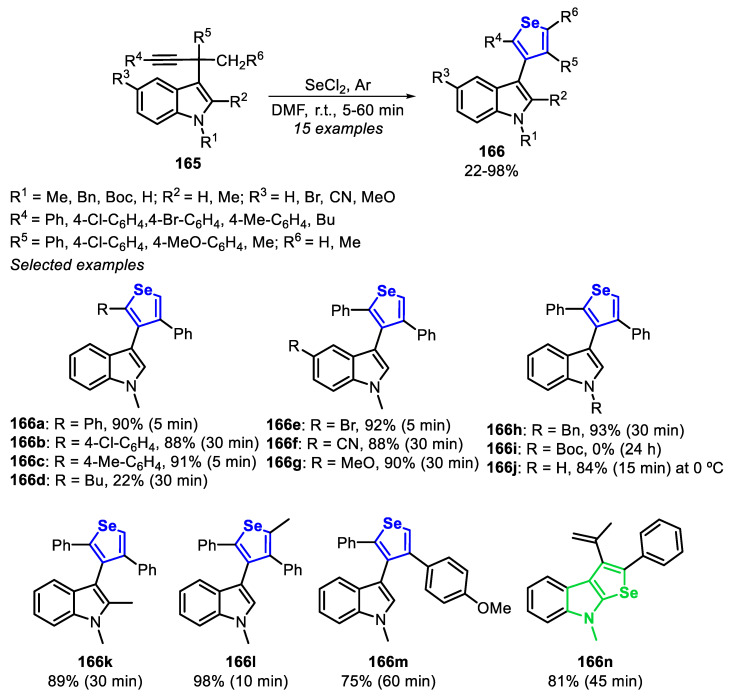
Scope of the synthesis of polysubstituted selenophenes **166**.

In 2018, the synthesis of 3-selenophen-3-yl-1*H*-indoles **166** through the aromatic electrophilic cyclization of propargyl indoles **165**, promoted by SeCl_2_, was described [184]. The electrophilic selenium species was prepared in situ by the reaction between SO_2_Cl_2_ and selenium powder in DMF at room temperature under an argon atmosphere. The versatility of this protocol was evaluated by using a diversity of propargyl indoles **165**, containing substituents at the terminal triple bond, at C-2, C-5, and at the nitrogen atom of the indole core, as well as the other two substituents at the propargylic position. Regarding the aryl group directly bonded to the alkyne, neutral, electron-rich, and electron-poor groups were able to give the desired products **166a**–**c** in good yields (88–91%) and short reaction times (5–30 min). The replacement of the aryl group by a less reactive aliphatic one caused a remarkable decrease in the reaction efficiency, affording the fused-selenophene **166d** an only 22% yield after 30 min. The reactivity of the substrate was not affected by modifications in the indole core at the C-5 position, and selenophenes **166e** (R = Br), **166f** (R = CN) and **166g** (R = MeO) were obtained at 88–92% yields, without any evident electronic effect. A similar good reactivity was observed when the 2-Me-indole derivative **165k** was employed, and the corresponding product **166k** was obtained in 89% yield. In contrast, the nature of the *N*-substituent was relevant to the reaction performance; while *N*-methyl and *N*-benzyl indoles were suitable substrates, affording the respective products in good to very good yields, no reaction was observed when the *N*-Boc derivative was used, and the selenophene **166i** (R = Boc) did not form, even after 24 h of reaction. Unsubstituted (*N*-H free) indole **165j** afforded the product **166j** in 84% yield after 15 min at 0 ^°^C. The low temperature was necessary to avoid the formation of coproducts, observed at room temperature. The presence of an electron-donating group at the *para*-position of the aromatic ring of the propargylic position in **165m** (R^5^ = 4-MeOC_6_H_4_) adversely affected the reaction performance, and the respective selenophene **166m** was obtained in 75% yield after 1 h. Interestingly, when a methyl group was present at the propargylic position, such as in **165n** (R^5^ = Ph, R^6^ = Me), the respective fused selenophene[2,3-*b*]indole **166n** was obtained in 81% yield after 45 min (Scheme 111).

**Scheme 112:**
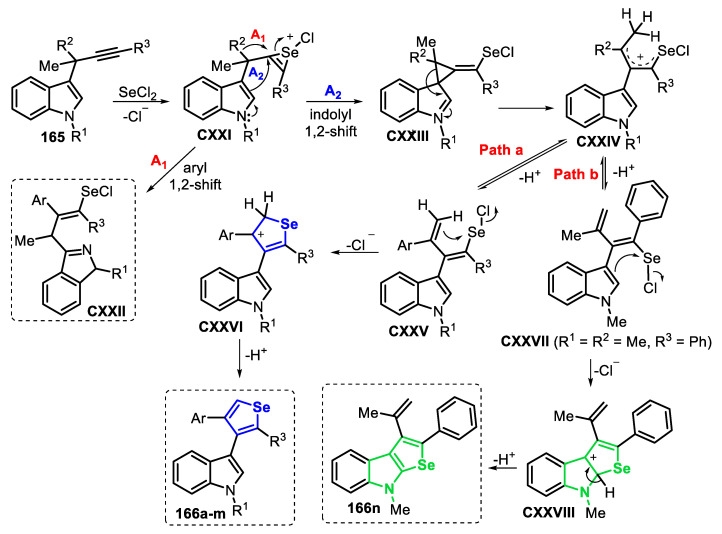
Proposed mechanism of the synthesis of polysubstituted selenophenes **166**.

The first step of the mechanism of the synthesis of selenophenes **166** is the electrophilic addition of SeCl_2_ to the C-C triple bond of the propargyl indole **165**, affording the seleniranium intermediate **CXXI** (Scheme 112). This triggers a cationic cascade process that could undergo at least three different evolutions, including a chloride-mediated and two intramolecular (routes A_1_ and A_2_) nucleophilic seleniranium ring-openings. In route A_1_, a 1,2-sigmatropic rearrangement of the aryl group (R^2^) results in the tertiary allyl carbocation intermediate **CXXII**, stabilized by the pendant 3-indolyl moiety. Alternatively, the intermediate **CXXI** could be involved in a 1,2-indolyl shift, with the aid of the lone pair of electrons of the indole nitrogen, affording the polysubstituted spirocycle **CXXIII** (route A_2_). The ring-opening of the seleniranium and the C-C bond formation, through S_N_2 attack of the indole C-3 position to the electron-deficient center, is a concerted process, which would avoid passing through a highly reactive vinyl cation species. Once formed, the intermediate **CXXIII** undergoes a ring-opening of the strained cyclopropane motif, affording the carbocation intermediate **CXXIV**. The migratory aptitudes of the migrating moieties are typically correlated to their electron-richness and their ability to stabilize a positive charge in the transition state/intermediate. When R^2^ is an aromatic substituent, the reaction mechanism would occur by Path a, by losing a proton from the intermediate **CXXIV**, creating the selenodiene **CXXV**, which is suitable for the cyclization to the cationic intermediate **CXXVI** to afford, finally, the trisubstituted selenophene products **166a**-**m**. Considering this, it is plausible that the presence of an alkyl R^3^ substituent may fail to provide a satisfactory stabilization to the carbocation intermediate **CXXVI**, resulting in lower product yields (such as **166d**). In contrast, the formation of the fused selenophene[2,3-*b*]indole **166n** suggests the involvement of a slightly different route (Path b). The carbocation intermediate **CXXIV** would lose a proton to give the selenodiene **CXXVII** a geometric isomer of **CXXV**. Then, the selenodiene **CXXVII** undergoes an intramolecular nucleophilic attack by the indole ring, affording the carbocation intermediate **CXXVIII** that, after deprotonation, affords the selenophene **166n**.

**Scheme 113:**
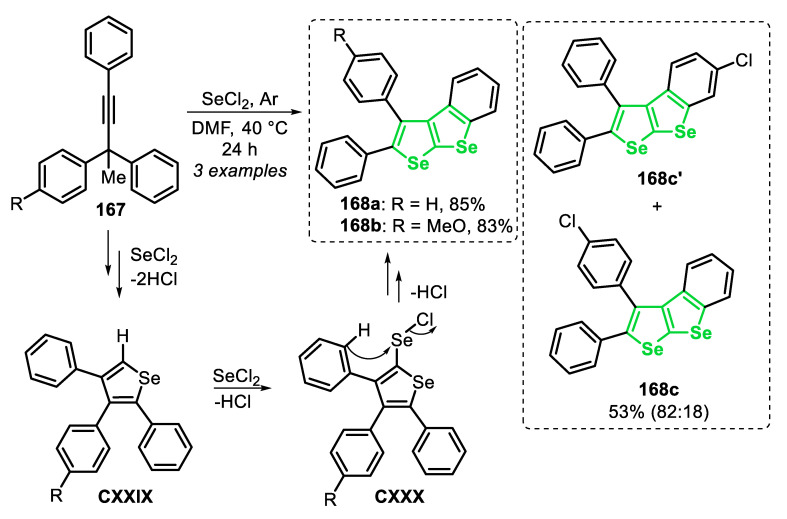
Scope and mechanism of the synthesis of 2,3-diaryl-benzo[*b*]selenopheno[3,2-*d*]selenophenes **168**.

In the same work, the authors evaluated the behavior of other aromatic substituents in the 1,2-shift process that initiates the cascade reaction toward substituted selenophenes. Thus, using 3-methyl-substituted 1,3,3-triphenyl-1-butynes **167** as starting materials in the reaction with SeCl_2_, under the previously optimized conditions, the corresponding fused-selenophenes **168a**–**c** were obtained in moderate to good yields (Scheme 113). These results confirmed that other aromatic rings are also able to promote the required initial 1,2-shift and suggest that the reaction could have a broader scope. Further, the isolation of **168c** as a mixture of products resulted from the migration of the phenyl and 4-chlorophenyl moieties, reinforced the conjecture that the migration aptitude is related to the electronic richness of the potentially migrating groups. These outcomes indicate that the formation of the benzo[*b*]selenopheno[3,2-*d*]selenophenes **168** occurs via the selenophene intermediate **CXXIX**, isostructural to the trisubstituted selenophenes **166a**–**m**. The intermediate **CXXIX** reacts with another equivalent of SeCl_2_ by a S_E_Ar, resulting in the chloroseleno intermediate **CXXX**, that undergoes an intramolecular cyclization to afford, after releasing HCl, the selenophene **168** (Scheme 113).

### 4.4. Other Approaches to Prepare Fused Selenophenes

**Scheme 114:**
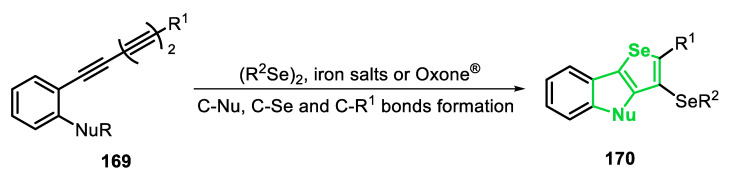
Other common strategies to access fused selenophenes.

Other efficient approaches for the synthesis of fused selenophenes **170** have been recently developed. For instance, the double intramolecular cyclization reaction of *ortho*-diynyl/triynyl chalcogenides **169**, using Se-based electrophilic species, generated in situ by the reaction between dialkyl diselenides and iron salts or Oxone^®^ (Scheme 114).

**Scheme 115:**
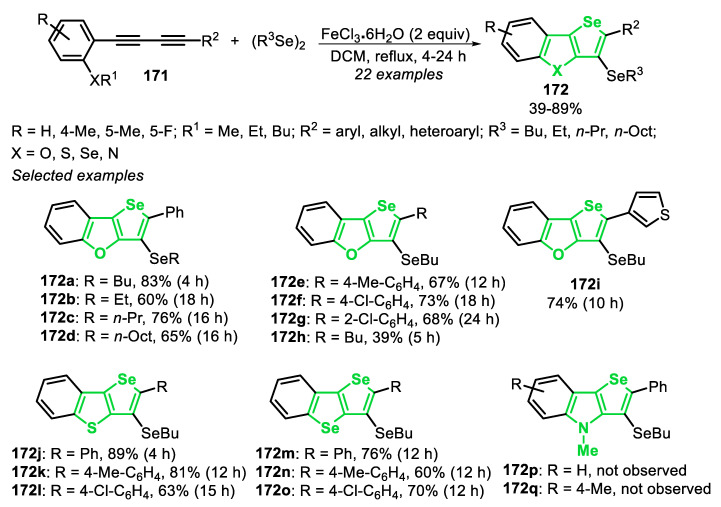
Scope of the synthesis of benzo[*b*]selenopheno[2,3-*d*]furans/thiophene/selenophenes **172**.

In this context, the synthesis of benzo[*b*]furan/thiophene/selenophene-fused selenophenes **172** through the double intramolecular 5-*endo*-*dig* cyclization of butyl[2-(phenylbuta-1,3-diynyl)phenyl]-chalcogens **171** was described (Scheme 115) [185]. The reaction was carried out in the presence of FeCl_3_·6H_2_O (2 equiv) and diorganyl diselenides (1.75 equiv) in refluxing DCM under air atmosphere for 4–24 h. The versatility of the protocol was firstly evaluated by using different dialkyl diselenides, giving the desired fused selenophenes **172a**–**d** in good yields. Several alkyl groups were used; however, butyl proved to be the more reactive one. These standard reaction conditions were also compatible with several 1,3-diynes bearing electron-rich and electron-poor aryl groups and 3-thyenyl, affording the respective products **172e**–**g** and **172i** in 67–74% yields. A lower yield was obtained when an alkyl 1,3-diyne was employed, probably due to the absence of π-bonds next to the alkyne moiety, which decreases the reactivity for the electrophilic attack at the C-C triple bond. The nucleophilic effect of a heteroatom other than oxygen was analyzed by the introduction of sulfur, selenium, and nitrogen atoms at the 2-position of the aryl group bonded to the alkyne. The cyclization reaction of sulfur and selenodiynes with dibutyl diselenide gave the fused selenophenes **172j**–**o** in 60–89% yields after 4–15 h. The cyclization of 1,3-diynes *ortho*-functionalized with nitrogen atom as a nucleophile, however, gave a mixture of byproducts.

**Scheme 116:**
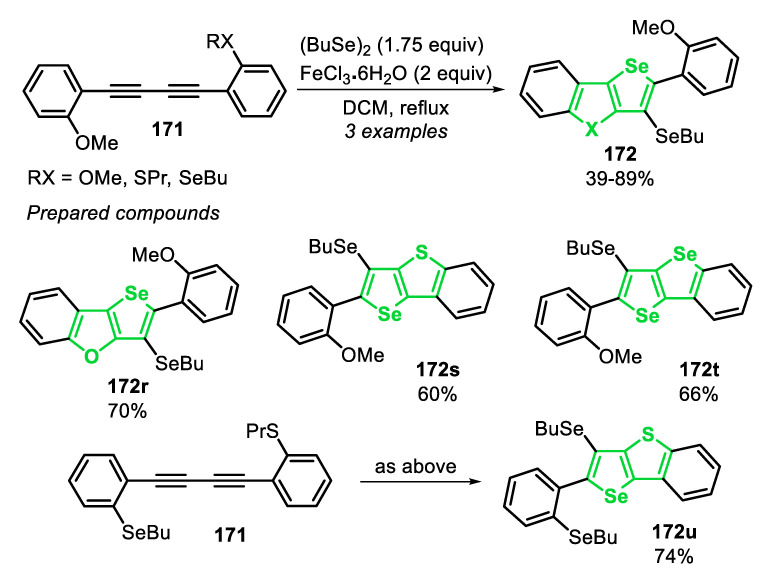
Competition between selenium, sulfur and oxygen as nucleophiles in the cyclization reaction.

In the same work, an interesting study using unsymmetrical 1,3-diynes bearing two potential nucleophiles was conducted; considering the differences in reactivity of the nucleophiles, the reaction could lead to different products of cyclization (Scheme 116). In the competitive cyclization reaction between the nucleophilic oxygen at the *ortho*-position of the aryl group and the sulfur or selenium at the *ortho*-position of the second aryl group, the selenophenes **172s** and **172t** were exclusively obtained, due to the higher nucleophilicity of sulfur and selenium compared to that of oxygen. In contrast, the cyclization of 1,3-diyne **171** involves the competition between selenium and sulfur nucleophiles and gave the product **172u**, from the sulfur nucleophilic attack, in 74% yield. In this case, although the nucleophilicity of the selenium atom is higher than that of the sulfur atom, the steric hindrance of the butyl group was determinant to the observed selectivity.

**Scheme 117:**
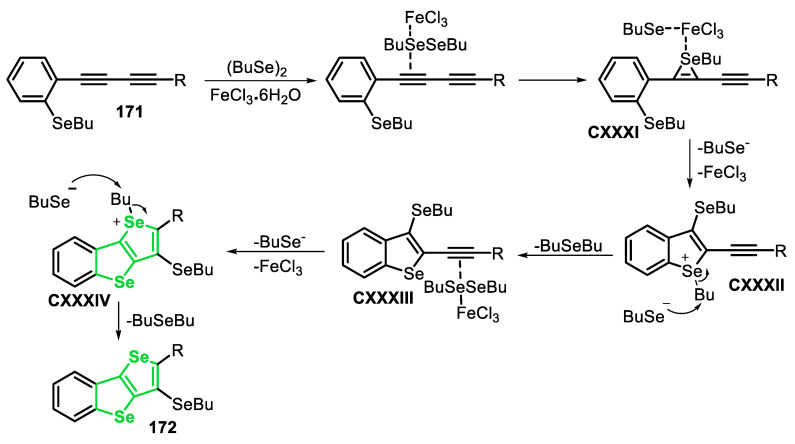
Proposed mechanism of the synthesis of **172**.

The proposed mechanism for the synthesis of the fused selenophenes **172** involves, firstly, the reaction between the iron salt and the dialkyl diselenide, promoting the heterogeneous cleavage of the Se-Se bond to give a butylselanyl cation and butylselenolate anion (Scheme 117). Then, the iron(III) coordination with one selenium atom from dibutyl diselenide activates the other one towards nucleophilic attack by the alkyne giving the seleniranium ion **CXXXI**. After an intramolecular nucleophilic *anti*-attack by the pendant *o*-selenium atom to the activated seleniranium ion **CXXXI**, the selenonium salt **CXXXII** and butylselenolate anion are formed. Then, the BuSe^-^ attacks the butyl group bonded to the selenium atom in the intermediate **CXXXII**, affording the 2-alkynyl benzoselenophene **CXXXIII**. In the sequence, a second similar cyclization process occurs with benzoselenophene **CXXXIII** to give the final product **172**.

**Scheme 118:**
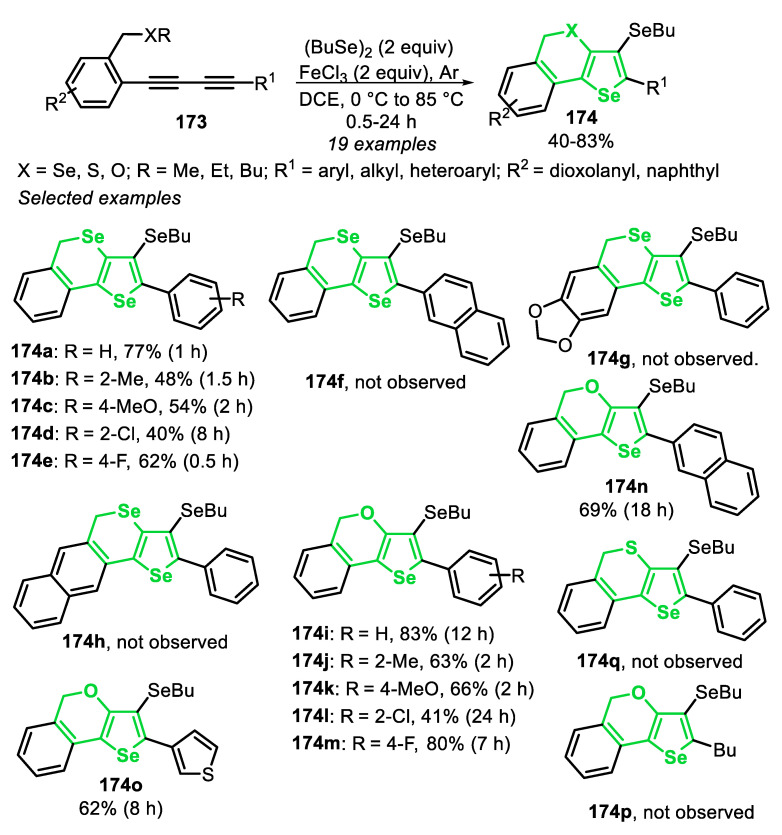
Scope of the synthesis of chalcogenochromene-fused selenophenes **174**.

In 2019, the use of the FeCl_3_/Bu_2_Se_2_-promoted cascade cyclization of *ortho*-diynyl benzyl chalcogenides **173** for the synthesis of isochromene-fused selenophene derivatives **174** was described (Scheme 118) [186]. A total of nineteen fused selenophenes **174** were prepared by stirring a mixture of the diyne **173**, FeCl_3_ and dibutyl diselenide in refluxing DCM under an argon atmosphere for 0.5–24 h. It was observed that *ortho*-diynyl benzyl selenide **173a**, containing a pendant unsubstituted phenyl group on the terminal alkyne (R^1^ = Ph) gave the respective selenophene **174a** in 77% yield after 1 h, while the presence of substituent at the *ortho*- (R^1^ = 2-Me-C_6_H_4_ and 2-Cl-C_6_H_4_) or *para*-positions (R^1^ = 4-MeOC_6_H_4_ and 4-FC_6_H_4_) of the phenyl caused a decrease in yields of products **174b**-**e**, which were obtained in 40%–62% yields. No reaction occurred when the naphthyl group was bonded on the terminal alkyne, or when dioxolane and naphthyl were present as substituent groups at the benzyl aryl ring. The use of oxygen as a nucleophile instead of selenium was evaluated. Although less nucleophilic than the selenium analogues, the benzyl methyl ethers were suitable substrates in the reaction, due the highly (Lewis) acidic and oxophilic character of iron(III), which probably support the nucleophilic substitution step allowing the cyclization. In general, larger reaction times (up to 24 h) were required to give the respective isochromene-fused selenophenes **174i**–**n** in 41%–83% yields. The protocol was compatible to diyne **173o**, containing the heteroaryl group 3-thienyl, giving the corresponding product **173o** in 62% yield after 8 h. No reaction was observed when an alkyl group was introduced at the carbon–carbon triple bond of the benzyl methyl selenoether **173p** (R^1^ = C_4_H_9_), as well as when the benzyl methyl thioether **173q** (XR = SMe) were substrates. In the case of thioether, the respective selenophene **174q** was not formed, probably because the sulfur atom is not sufficiently nucleophilic to promote the cyclization step.

**Scheme 119:**
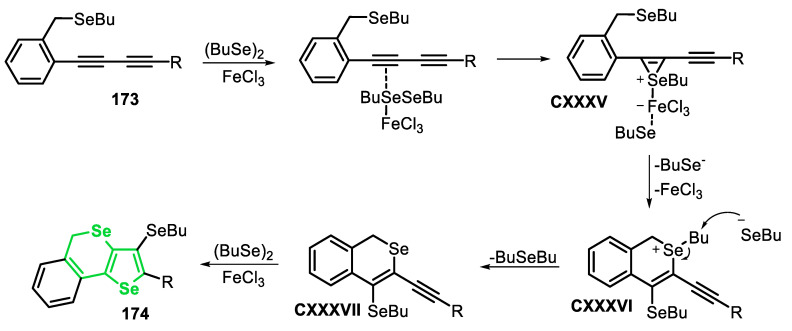
Mechanism of the synthesis of 3-(butylselanyl)-5*H*-selenopheno[3,2-*c*]isoselenochromene **174**.

The proposed mechanism for the synthesis of the fused selenophenes **174** is similar to that described in Scheme 119, for the synthesis of **172**. Firstly, FeCl_3_ reacts with dibutyl diselenide promoting a Se-Se bond heterolytic cleavage to form a butylselanyl cation and butylselenolate anion. Then, the electrophilic portion of dibutyl diselenide adds to the C-C triple bond of the substrate **173**, generating the three-membered cyclic seleniranium cation **CXXXV**. Subsequently, an intramolecular attack by the selenium atom from the benzylic position occurs, affording the isoselenochromene intermediate **CXXXVI**, through a 6-*endo-dig* cyclization and releasing BuSe^-^ to the reaction medium. In the sequence, the butyl group bonded to the selenium cation is removed by the reaction with butylselenolate, giving the 4-(butylselanyl)-isoselenochromene **CXXXVII**. Finally, this intermediary reacts with iron(III) chloride and dibutyl diselenide again, in a second cyclization reaction, now via a 5-*endo-dig* mode, to give the product **174** (Scheme 119).

**Scheme 120:**
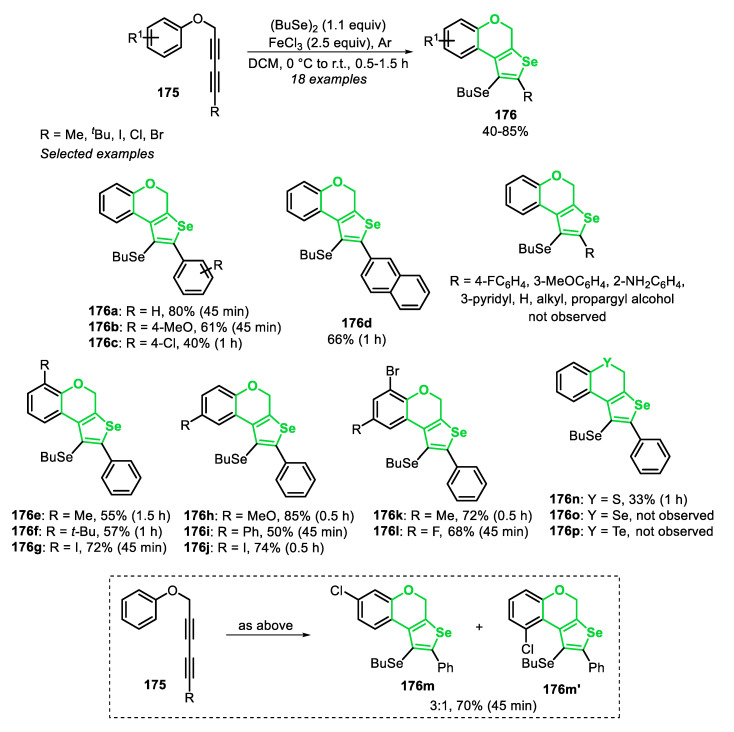
Scope of the synthesis of 1-(butylselanyl)-selenophene-fused chromenes **176**.

This year (2020), an alternative approach for the synthesis of selenophene-fused chromene derivatives **176** was described [187]. The method involves the one-pot formation of C-C, C-Se, Se-C and C-Se bonds, using FeCl_3_/Bu_2_Se_2_ to promote the cyclization reaction of 1,3-diynyl propargyl aryl ethers **175**, in DCM, at room temperature under argon atmosphere (Scheme 120). The extent and limitations of the protocol was firstly investigated by evaluating the influence of the substituents directly bonded to the alkyne triple bond. The electron-rich 1,3-diynyl propargyl aryl ether **175b** (R = 4-MeO-C_6_H_4_) reacted under the optimal conditions to afford the respective fused selenophene **176b** in 61% yield after 45 min, while the electron-poor **175c** (R = 4-Cl-C_6_H_4_) was less reactive, giving **176c** in 40% yield after 1 h. An equally good result was obtained using the sterically hindered alkyne **175d** (R = 2-naphthyl), and the selenophene derivative **176d** was isolated in 66% yield after 1 h of reaction. According to the authors, the expected products from 1,3-diynyl propargyl aryl ethers with *para*-fluoro, *meta*-methoxy and *ortho*-amino phenyl groups and 3-pyridyl could not be isolated, due to their decomposition during the purification process. The tentative reactions starting from alkyl, propargyl alcohol, and terminal 1,3-diynes hydrogen failed once the starting materials were unstable under the reaction conditions. Regarding the phenyl group directly bonded to the oxygen atom of the substrate **175**, the influence of the presence of *ortho*- and *para*-substituents was evaluated, and the respective cyclized products **176e**-**l** were obtained in moderate to good yields. The regioselectivity of the nucleophilic attack of the aromatic ring to the triple bond was studied by the reaction of the *meta*-chloro-substituted 1,3-diyne **175m**. In this case, it was observed the formation of a 3:1 mixture (determined by GC/MS) of **176m** and **176m’** in 70% overall yield. The moderate regioselectivity in favor of **176m** may be influenced by the steric hindrance caused by the chlorine atom in the **176m’** regioisomer. The reaction was explored in the cyclization of other chalcogenoethers (S, Se and Te), and only the thioether derivative was a suitable substrate, affording the respective thiochromene derivative **176n** in only 33% yield. This outcome indicates a strong influence of the identity of the chalcogen atom in the reactivity of the whole molecule.

**Scheme 121:**
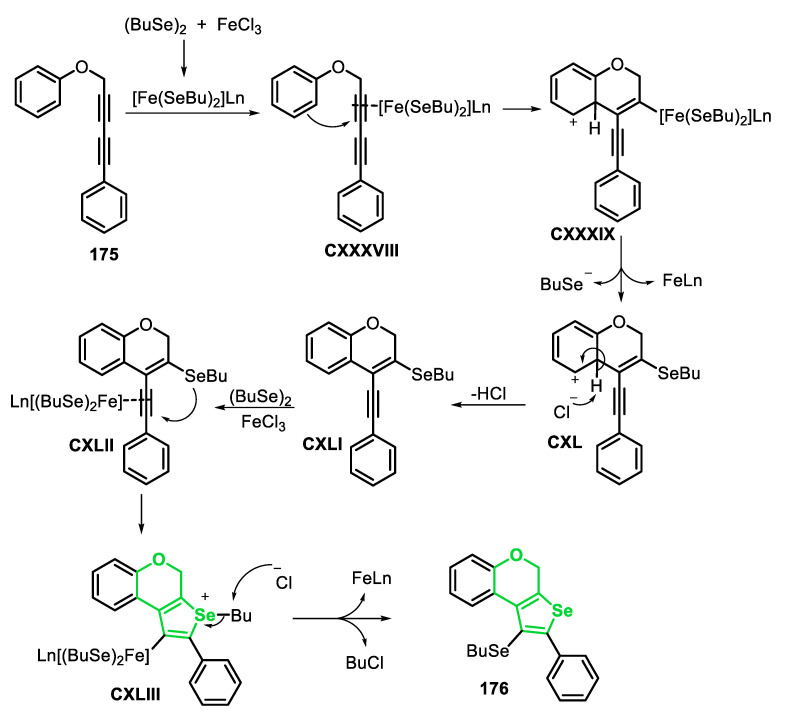
Proposed mechanism of the synthesis of 4*H*-selenopheno[2,3-*c*]chromene **176**.

The first step of the reaction mechanism is the formation of a butylselenium iron complex, which activates the triple of the substrate **175**, forming the intermediate **CXXXVIII**. Then, an attack of the electron cloud from the aromatic ring at the activated intermediate **CXXXVIII** occurs, giving the cyclized species **CXXXIX**, which rapidly releases butylselenolate and iron salt to the reaction medium, to form the intermediate **CXL**. The removal of a proton from intermediate **CXL** by a chloride anion, restores the aromaticity, giving the 3-(butylselanyl)-2*H*-chromene **CXLI**. In the sequence, the organoselenium iron complex promotes the activation of the remaining triple bond of intermediate **CXLI**, and an intramolecular nucleophilic attack by the selenium generates the selenonium **CXLII**. The removal of the butyl group bonded to the selenium atom by the chloride ion via an S_N_2 displacement affords the selenophene chromene derivative **176** and butyl chloride (detected by GC/MS) (Scheme 121).

**Scheme 122:**
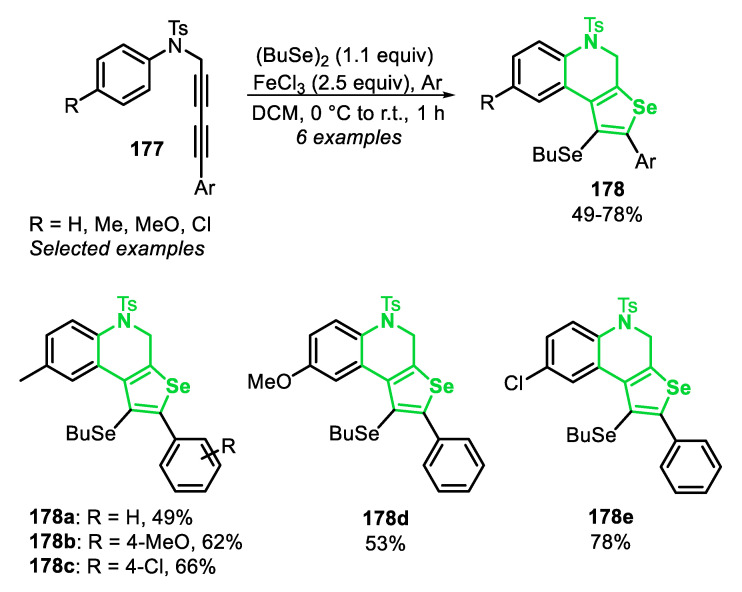
Scope of the synthesis of selenophene-fused quinolines **178**.

The optimal conditions were successfully extended to the cyclization of propargyl anilines **177**, leading to the formation of 1-(butylselanyl)-selenophene-fused quinolines **178**. The 1,3-Diynes containing both electron-donor or electron-withdrawing groups at the pendant aromatic rings were suitable substrates for the reaction, and the respective products **178a**–**e** were obtained in 49%–78% yield after 1 h (Scheme 122).

**Scheme 123:**
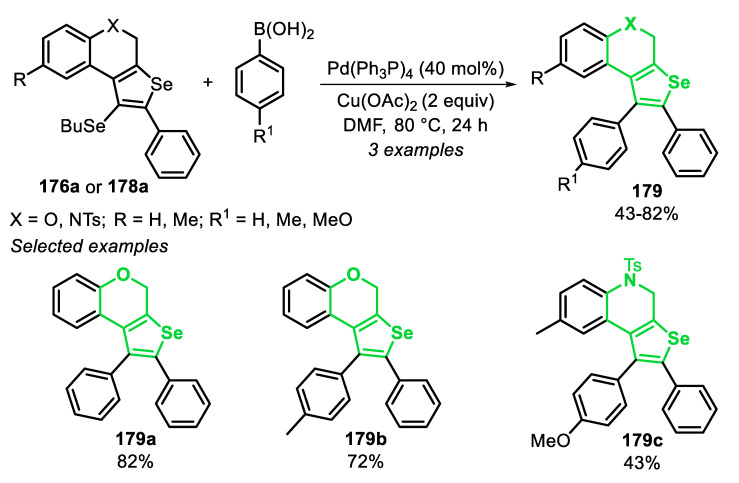
Suzuki cross-coupling reactions of **176a** and **178a**.

The reactivity of the new fused-selenophenes **176a** and **178a** was explored in the Pd-catalyzed Suzuki cross-coupling reaction with arylboronic acids (Scheme 123). The selenophene-fused chromene **176a** reacted with phenyl and *p*-tolylboronic acids under Suzuki conditions to produce the corresponding products **179a** and **179b** in 82% and 72% yields, respectively. Similarly, the selenophene-fused quinoline **178a** coupled with 4-methoxyphenylboronic acid to give **179c** in 43% yield.

**Scheme 124:**
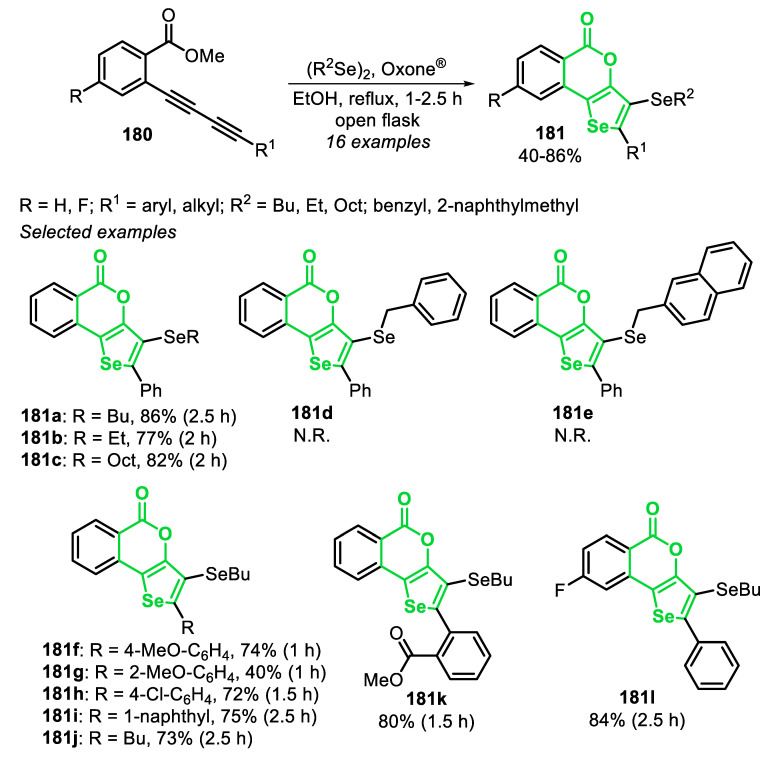
Scope of the synthesis of isochromenones fused to selenophenes **181**.

**Scheme 125:**
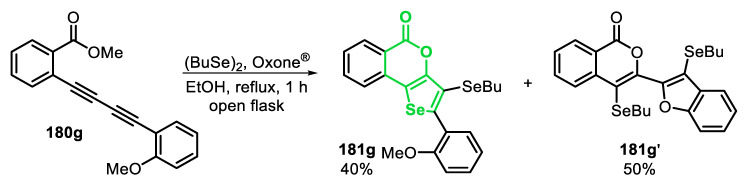
Seleno- and oxo-cyclization competing reactions.

**Scheme 126:**
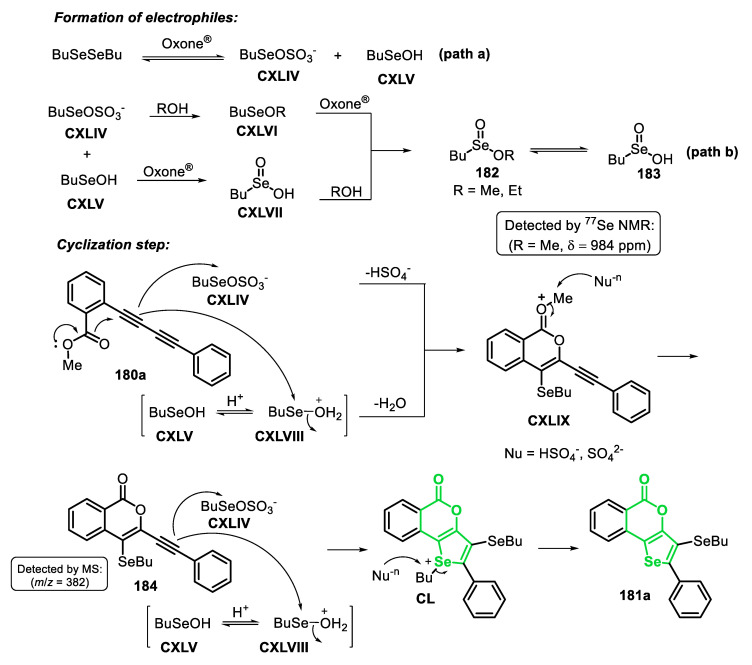
Mechanism of the synthesis of isochromenones fused to selenophene **181a**.

In 2019, some of us [188] reported the synthesis of 5-*H-*selenopheno[3,2-*c*]isochromen-5-ones **181** through a double intramolecular cyclization of methyl 2-(organyl-1,3-diynyl)benzoate **180** promoted by electrophilic species of selenium generated in situ by the reaction of dialkyl diselenides with Oxone^®^, under the reflux of EtOH, in an open flask (Scheme 124). This protocol was suitable for different dialkyl diselenides (R^2^ = Bu, Et, Oct), which reacted with the diyne **180** to give the expected isochromenones **181a**-**c** in 77–86% yields, while no reaction was observed starting from dibenzyl- and bis(2-naphthylmethyl)diselenide. Regarding the 1,3-diyne counterpart, both electron-releasing and electron-withdrawing groups at the *para*-position of the pendant phenyl ring (R^1^) of **180** were tolerated in the reaction with dibutyl diselenide (**181f**–**h**). Similar results were obtained when naphthyl and butyl groups are directly bonded to the alkyne. An interesting result was obtained when *ortho*-methoxy-1,3-diyne **180g** (R^1^ = 2-MeOC_6_H_4_) was used as substrate, with the desired fused selenophene **181g** being obtained in only 40% yield (Scheme 125). This result can be explained by the two competing intramolecular *Se*-cyclization and the *O*-cyclization reactions in the intermediate **184c**, formed in the first step of the reaction (see Scheme 126, for the reaction mechanism), producing the benzofuran derivative **181g’** in 50% yield as a coproduct of the reaction. However, when the dimer dimethyl 2,2′-(buta-1,3-diyne-1,4-diyl)dibenzoate was submitted the optimal conditions in the reaction with Bu_2_Se_2_, the double intramolecular *O*-cyclization product was not formed, and the fused isochromenones selenophene **181k** was obtained in 80% yield after 1.5 h. The presence of a fluor-group at the pendant ring of the ester group in **180l** (R = F) did not alter the reactivity, affording the respective selenophene **181l** in 84% yield after 2.5 h (Scheme 124).

To better understand the mechanism for this cyclization reaction, a series of control experiments were carried out by the authors. For the identification of the active electrophilic selenium species involved in the reaction, ^77^Se NMR spectra were collected from the reaction mixture between Oxone^®^ and dibutyl diselenide in an NMR tube, using MeOD-*d*_4_ as the solvent. This was observed as a signal at 984 ppm, which was attributed to methyl butane-1-seleninate **182**, formed from the reaction between butylseleninic acid **183** and MeOD-*d*_4_. The formation of butylseleninic acid **182** is a result of the overoxidation of dibutyl diselenide by Oxone^®^, which could pass by the intermediates BuSeOSO_3_^-^ (**CXLIV**) and BuSeOH (**CXLV**). The reaction between the diyne **180a** and Bu_2_Se_2_/Oxone^®^ was monitored by GC/MS. Aliquots of the reaction mixture were collected, pretreated by a “micro work-up” and injected into the GC/MS spectrometer. This experiment was carried out under the optimal conditions, and aliquots were collected at 15, 30, 60 and 120 min. From these experiments, the intermediate **184** was identified (formed by *O*-cyclization reactions). With these elements in hand, a mechanism was proposed, which firstly involves the reaction between dibutyl diselenide and Oxone^®^ to form the intermediates **CXLIV** and **CXLV** (Scheme 126, path a). The species **CXLV** can react with H^+^ from the reaction medium, leading to BuSeOH_2_^+^ (**CXLVIII**). After, diyne **180a** reacts with **CXLIV** and **CXLVIII**, leading to the cyclic intermediate **CXLIX**, releasing HSO_4_^-^ and H_2_O to the medium. The displacement of the methyl group from **CXLIX** by a nucleophile (HSO_4_^-^ from previous step and/or SO_4_^2^^-^, from Oxone^®^), affords the key intermediate **184**. Following this, the remaining triple bond of **187** reacts in the same way with **CXLIV** and **CXLVIII**, giving the fused-selenophene cation intermediate **CL**. The displacement of the butyl group from the selenonium cation affords the expected 5*H*-selenopheno[3,2-*c*]isochromen-5-one **181a**.

**Scheme 127:**
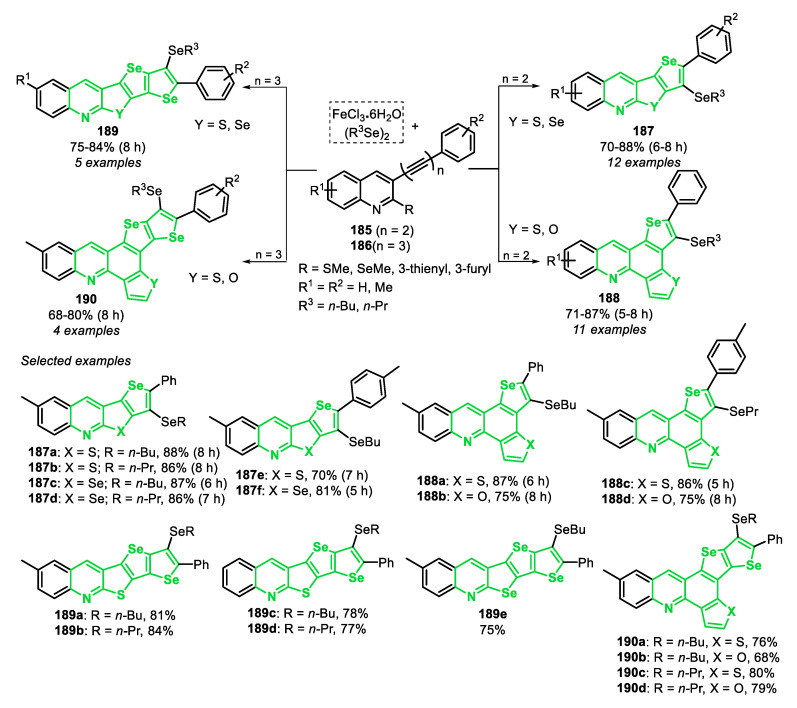
Scope of the synthesis of selenophene and diselenophene-fused compounds.

In 2019, the Fe(III)-promoted intramolecular cascade cyclization of 1,3-diynes **185** and 1,3,5-triynes **186** under reflux of DCM for the construction of selenophene-fused, quinoline- and acridine-based heteroacene scaffolds **187**–**190** was described (Scheme 127) [189]. In this protocol, the 1,3-diyne and 1,3,5-triyne were cyclized through diversified internal nucleophiles by using dialkyl diselenides, which play a dual role, as a cyclizing agent and in the insertion of one and/or two selenium atoms and one R^2^Se group in the final product. The synthesis of selenophene-fused thieno/selenophene[2,3-*b*]quinolines **187** and selenophene-fused thieno/furo[2,3-*c*]acridine **188** was evaluated in the electrophilic cyclization of *ortho*-functionalized 1,3-diynes with SMe/SeMe or 3-thionyl/3-furyl, respectively, using the FeCl_3_·6H_2_O/dialkyl diselenide system (2.5:2 equiv). Under these reaction conditions, several selenophene-fused quinoline/acridine **187** and **188** were obtained in 70–88% after 5–8 h. The cascade cyclization of 1,3,5-triynes **186** was achieved in the presence of Fe(III) (3 equiv) and dibutyl diselenide (2.5 equiv) conditions, and several diselenophene-fused thieno/selenophene-[2,3-*b*]quinolines **188**, and diselenophene-fused thieno/furo[2,3-*c*]acridine **190** were obtained in 68%–84% yields after 8 h.

**Scheme 128:**
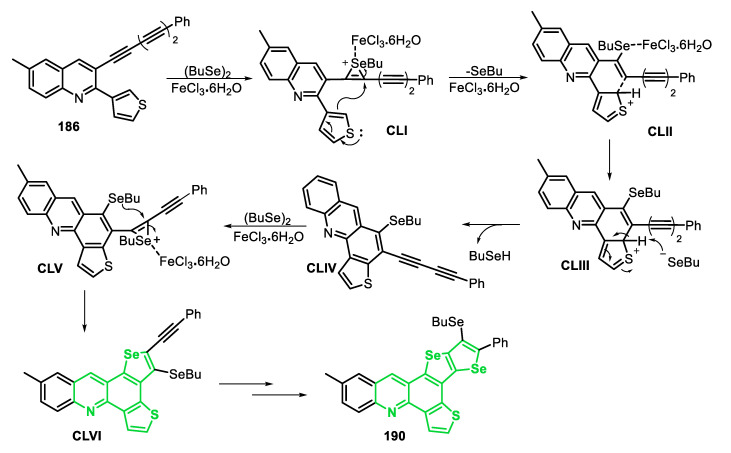
Mechanism of the synthesis of diselenophene-fused thieno[2,3-*c*]acridine **190**.

In this reaction, when 3-furan and 3-thiophene were used as internal nucleophiles, the nucleophilic attack takes place from the *ortho*-position of furan and thiophene, which interestingly resulted in six-membered acridine-core heterocycles **188** and **190**. On the other hand, when sulfur and selenium were used instead, five-membered heterocycles **188** and **189** were obtained (Scheme 127). Based on these results, a mechanism for the synthesis of diselenophene-fused thiene[2,3-*c*]acridine **190** was proposed, which involves, in the first step, the reaction of iron salt with dibutyl diselenide to promote the heterolytic cleavage of the Se-Se bond, producing the butylselanyl cation and butylselenolate anion (Scheme 128). After the Fe(III) coordinates with one selenium atom from dibutyl diselenide, it favors the addition to a triple bond and affording the seleniranium intermediate **CLI**. In the sequence, the intermediate **CLI** undergoes an *anti*-attack by the thiophene nucleophile, resulting in the cyclic intermediate **CLII**. The rearomatization of intermediate **CLIII** is achieved via the S_N_2 displacement by the butylselenolate anion, giving the thieno[2,3-*c*]acridine **CLIV**. The second cyclization step proceeds with the BuSe nucleophilic moiety in **CLV**, which resulted in the intermediate selenophene-fused thieno[2,3-*c*]acridine **CLVI**. On continuation, the cascade cyclization proceeds for the remaining triple bond in **CLVI**, which finally affords the product diselenophene-fused thieno[2,3-*c*]acridine **190**.

**Scheme 129:**
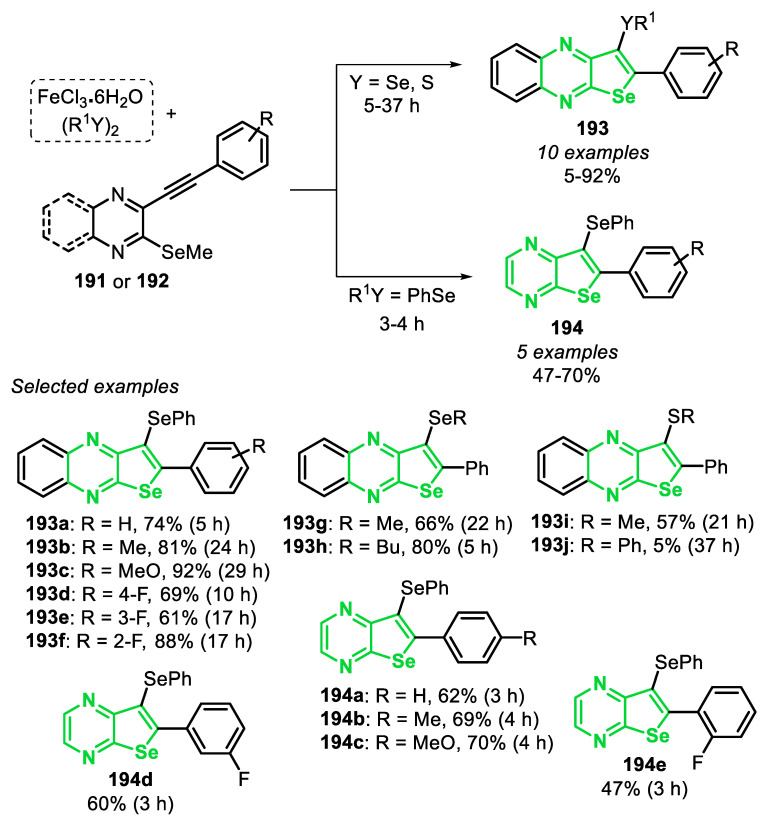
Scope for the synthesis of 3-(arylselanyl/sulfanyl)-2-arylselenopheno[2,3-*b*]quinoxaline **193** and 6-aryl-7-(phenylselanyl)selenopheno[2,3-*b*]pyrazine derivatives **194**.

The same strategy for the selenocyclization of properly substituted alkynes was used this year for the synthesis of 3-(aryl/alkylselanyl)-2-arylselenophene[2,3-*b*]quinoxaline **193** and 6-aryl-7-(phenylselanyl)selenopheno[2,3-*b*]pyrazine **194** [190]. The authors developed a Fe(III)-promoted electrophilic cascade cyclization of 2-(methylselanyl)-3-(arylethynyl)quinoxaline **191** and 2-(methylselanyl)-3-(arylethynyl)pyrazine **192**, respectively, using diorganyl diselenides and diorganyl disulfides as chalcogen sources, in DCM at 45 °C (Scheme 129). Differently substituted 2-(methylselanyl)-3-(arylethynyl)quinoxaline **191** bearing neutral (R = H), electron-donor (R = 4-Me and 4-MeO) and electron-withdrawing (R = 4-F, 3-F and 2-F) groups were suitable substrates in the reaction using FeCl_3_/PhSeSePh, affording the corresponding 3-(phenylselanyl)-2-arylselenopheno[2,3-*b*]quinoxaline **193a**-**f** in moderate to good yields (61–92%). The presence of substituents in the phenyl ring reduced the reactivity of the substrate (**193a** vs. **193b**-**f**), and this decrease was less pronounced in the case of the electron-deficient alkynes. When alkyl diselenides (dibutyl and dimethyl) were employed instead of diphenyl diselenide, the respective selenophenes **193g** and **193h** were obtained in 66% and 80% yields after 22 and 5 h, respectively. Diorganyl disulfides were suitable dichalcogenides in the reaction, despite the low yields compared to the diselenides analogues. The reaction was more efficient using dimethyl disulfide in comparation to diphenyl disulfide, and the sulfides **193i** and **193j** were obtained in 57% and 5% yields, after 21 and 37 h, respectively. The developed strategy was suitable for the cyclization of differently substituted 2-(methylselanyl)-3-(arylethynyl)pyrazines **192** by diphenyl diselenide, giving the respective 6-aryl-7-(phenylselanyl)selenopheno[2,3-*b*]pyrazine derivatives **194a**-**e** in 47%–70% yields after 3–4 h, and there does not seem to be a clear electronic effect from the aromatic ring. The proposed mechanism for the novel seleno-cascade cyclization is similar to that described in Scheme 128.

**Scheme 130:**
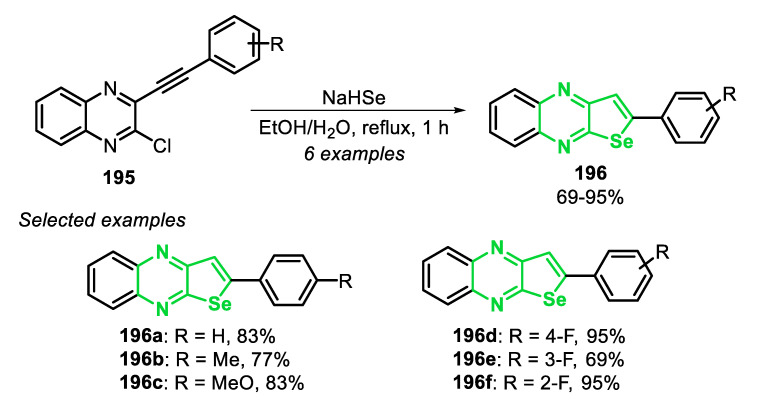
Scope of the synthesis of 2-arylselenopheno[2,3-*b*]quinoxaline derivatives **196**.

In the same work [190], the authors described the synthesis of 2-arylselenopheno[2,3-*b*]quinoxaline derivatives **196** through a S_N_Ar reaction of 2-chloro-3-(arylethynyl)quinoxaline derivatives **195** using NaHSe as a nucleophile (Scheme 130). NaHSe was prepared in situ by the reaction of elemental selenium (1 equiv) with sodium borohydride (2 equiv) in a 1:2 EtOH/H_2_O mixture at 0 °C for 15 min. Once the color of the reaction mixture changed from black to colorless, indicating the formation of NaHSe, a solution of 2-chloro-3-(arylethynyl)quinoxaline **195** (1 equiv) in ethanol was added, and the resulting mixture was stirred under reflux. After 1 h of reaction, the corresponding 2-arylselenopheno[2,3-*b*]quinoxaline derivatives **196a**-**f** were obtained in 69–95% yield. The lower reactivity was observed starting from 3-fluoro-substituted phenyl derivative **195e** (R = 3-F) while the best substrate for the reaction was **195f**, substituted with a fluorine atom at the *ortho*-position (R = 2-F).

**Scheme 131:**
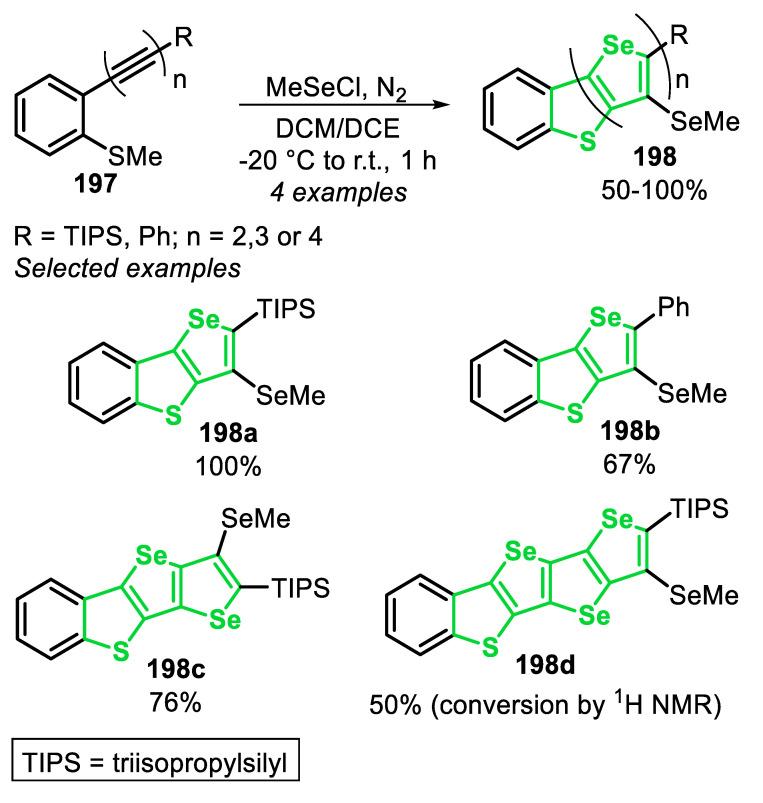
Scope for the synthesis of polyselenophenes fused to benzothiophenes **198**.

Recently, the one-step polyelectrophilic cyclization of polyynes **197** using the ambiphilic reagent MeSeCl for the synthesis of polyselenophenes fused to benzothiophenes **198** in DCM/DCE as solvent under N_2_ atmosphere for 1 h was reported (Scheme 131) [191]. Under these reaction conditions, the fused selenophenes **198a**-**c** were obtained in moderate to excellent yields (67–100%). In the tetracyclization sequence to form the polyselenophene **198d**, the fully annulated product was obtained together with a complex mixture of other byproducts (suspected MeSe/Cl addition products). This drawback was circumvented by heating the reaction mixture in refluxing DCE for 48 h, giving semi-pure **198d** in 50% yield (by ^1^H NMR). Chromatographic separation of **198d** from these byproducts proved to be difficult, and recrystallization afforded pure **198d** in only 10% yield. The ambiphilic reagent MeSeCl was prepared by the reaction of dimethyl diselenide with sulfuryl chloride (SO_2_Cl_2_) in dichloromethane at 0 °C under a nitrogen atmosphere, with a loss of SO_2(g)_.

**Scheme 132:**
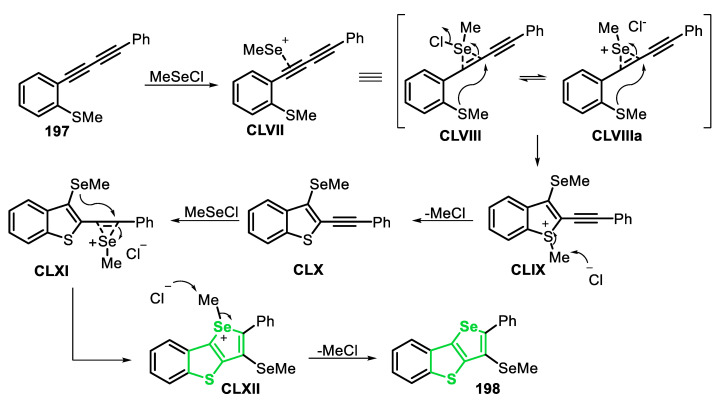
Proposed mechanism for the synthesis of selenophene-fused benzothiophene.

The proposed mechanism of the polyelectrophilic cyclization of polyynes **197** with MeSeCl for the synthesis of selenophenes fused to benzothiophenes **198**, is similar to those previously described selenocyclizations. Firstly, there is the activation of the triple bond by the MeSeCl, forming the complex **CLVII**, which could exist as the equilibrium between **CLVIII** and **CLVIIIa**. Then, the seleniranion **CLVIIIa** undergoes an intramolecular nucleophilic attack by the sulfur atom, producing the benzothiophene cation **CLIX** through a 5-*endo-dig* cyclization (Scheme 132). The removal of the methyl group bonded to the sulfur atom via S_N_2 displacement by the chloride ion affords the intermediate **CLX**. Finally, a second cyclization process occurs with intermediate **CLX** to give the final product **198**.

**Scheme 133:**
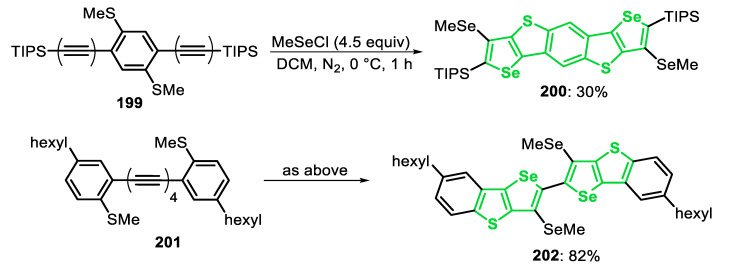
Synthesis of fused selenophenes **200** and **202** by biPEC.

The protocol depicted in Scheme 131 was extended to the bidirectional polyelectrophilic cyclization (biPEC) of tetraynes **199** and **201** using 4.5 equiv of MeSeCl. The diverging biPEC of **199** afforded the fused selenophene **200** in only 30% yield, probably due to the poor solubility of the product. In contrast, the converging biPEC of tetrayne **201** produced the dibenzo[*b*]selenopheno[2,3-*d*]thiophene **202** in 82% yield (Scheme 133).

**Scheme 134:**
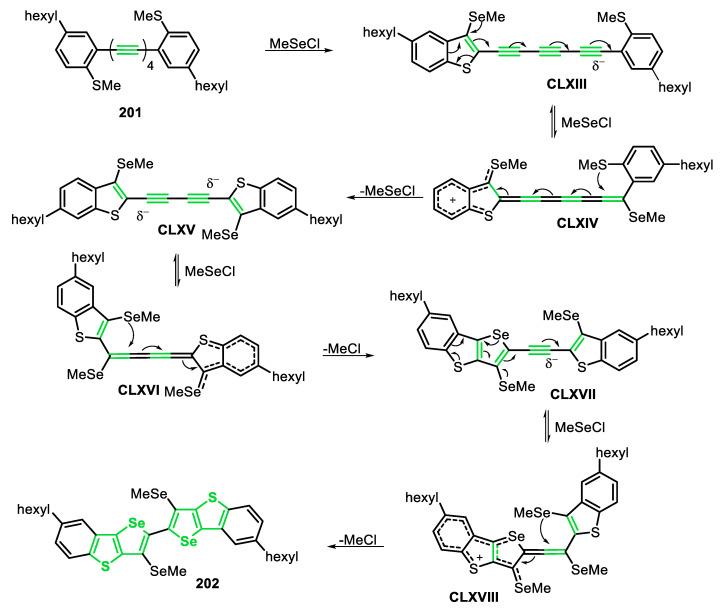
Proposed mechanism for the synthesis of dibenzo[*b*]selenopheno[2,3-*d*]thiophene **202**.

The synthesis of dibenzo[*b*]selenopheno[2,3-*d*]thiophene **202** from tetrayne **201** with MeSeCl occurs by a “seesawing” mechanism, involving alternating shifts in electron density through the polyyne chain (Scheme 134). The reaction initiates by the cyclization of the symmetrical tetrayne **201** with MeSeCl to give the monocyclized product **CLXIII**. Then, the resultant electron-rich 3-(methylseleno)-benzothiophenyl group (it is more electron-rich than the phenyl one) drives the electron density to distal alkynyl carbon (indicated by *δ*^−^), favoring the cyclization via the stabilized cation intermediate **CLXIV**, giving the diyne **CLXV**. In the sequence, the monocylization of symmetrical diyne **CLXV**, via the intermediate **CLXVI**, gives the unsymmetrical alkyne **CLXVII**, that is finally cyclized after attack by MeSeCl to the remote carbon, favoring the cyclization through the more stable cation **CLXVIII**, to give the product **202**.

## 5. Conclusions

This review highlights the protocols that were developed in recent years for the synthesis of selenophenes and their derivatives, as well as the mechanistic insights of the different reactions. Most of the synthetic approaches covered in this review are conducted under relatively mild conditions and tolerate a wide variety of functional groups. However, there is still room to improve existing synthetic methods, as well as to develop new ones. We believe that, in the coming years, there will be a further development of protocols to access the selenophene core, especially its fused derivatives. In addition, we see good prospects in the use of these derivatives as building blocks in the formation of new carbon–carbon bonds, in pharmacological enhancement, in addition to the emergence of new materials based on selenophene for optoelectronic applications. In this sense, we hope that this review can stimulate the research in preparative methods to access this peculiar class of organoselenium compounds, thus providing new alternatives for accessing the selenophene family, through more efficient, robust, and greener methods.
